# Testing gene–environment interactions in the presence of confounders and mismeasured environmental exposures

**DOI:** 10.1093/g3journal/jkab236

**Published:** 2021-07-16

**Authors:** Chao Cheng, Donna Spiegelman, Zuoheng Wang, Molin Wang

**Affiliations:** 1 Department of Biostatistics, Yale School of Public Health, New Haven, CT 06510, USA; 2 Center for Methods in Implementation and Prevention Science, Yale School of Public Health, New Haven, CT 06510, USA; 3 Department of Epidemiology and Biostatistics, Harvard T.H. Chan School of Public Health, Boston, MA 02115, USA; 4 Channing Division of Network Medicine, Department of Medicine, Brigham and Women’s Hospital, Harvard Medical School, Boston, MA 02115, USA

**Keywords:** computational efficiency, confounders, gene–environment interaction, measurement error, relative efficiency, reverse test

## Abstract

Interest in investigating gene–environment (GxE) interactions has rapidly increased over the last decade. Although GxE interactions have been extremely investigated in large studies, few such effects have been identified and replicated, highlighting the need to develop statistical GxE tests with greater statistical power. The reverse test has been proposed for testing the interaction effect between continuous exposure and genetic variants in relation to a binary disease outcome, which leverages the idea of linear discriminant analysis, significantly increasing statistical power comparing to the standard logistic regression approach. However, this reverse approach did not take into consideration adjustment for confounders. Since GxE interaction studies are inherently nonexperimental, adjusting for potential confounding effects is critical for valid evaluation of GxE interactions. In this study, we extend the reverse test to allow for confounders. The proposed reverse test also allows for exposure measurement errors as typically occurs. Extensive simulation experiments demonstrated that the proposed method not only provides greater statistical power under most simulation scenarios but also provides substantive computational efficiency, which achieves a computation time that is more than sevenfold less than that of the standard logistic regression test. In an illustrative example, we applied the proposed approach to the Veterans Aging Cohort Study (VACS) to search for genetic susceptibility loci modifying the smoking-HIV status association.

## Introduction

It is commonly believed that genetic variants can explain a proportion of the risk for most complex human diseases (*e.g.*, cancer, diabetes, and asthma), where additional unexplained risk could be explained, in part, by gene–environment (GxE) interactions (Thomas 2010). Although many studies have attempted to identify genetic susceptibility loci interacting with environmental exposures in samples using logistic regression analysis, few such interactions have been identified and replicated (Aschard *et al.* 2012). Reasons for the failure to detect GxE interactions may include: (1) insufficient power for testing interaction effects compared to testing main effects (Smith and Day 1984); (2) measurement errors in the environmental exposure sacrifice the power of the statistical test; (3) relatively small interaction effect sizes compared to the main effect sizes of genetic variants and environmental exposures, and (4) a large number of interactions to be tested as compared to a relatively small sample size in epidemiological studies. In addition, since GxE research is inherently nonexperimental, it is important to control for potential confounders (Keller 2014). For example, it is important to adjust for population stratification so that the interaction effects detected between genetic variants and exposures are not driven by ethnicity (Witte *et al.* 1999; Kraft and Hunter 2005; Wang *et al.* 2006; Wang and Lee 2008). In an attempt to minimize the number of covariates in the logistic regression model due to the concern about low power, investigators usually enter the confounders as covariates into the regression model, and ignore the potential confounder-environment or confounder-gene interaction effects. Several authors (*e.g.*, VanderWeele *et al.* 2013; Keller 2014) suggested to take into consideration the possible confounder-environment and confounder-gene interactions in order to obtain a more robust analysis result.

In this study, we focus on statistical methods for testing the interaction effect between a genetic variant and a continuous environmental exposure on a binary disease outcome. The genetic variant can be either binary or ordinal. When the environmental exposure is a continuous variable, Aschard *et al.* (2018) proposed a test that “reverses” the role of the disease status and the continuous exposure in the logistic regression model, *i.e.*, treating the disease status as an independent variable and the environmental exposure as the dependent variable. This regression model is now a linear regression including an interaction term between the genetic variant and disease status, encoding the GxE interaction effect. When the error term in this linear regression model follows a constant-variance normal distribution, both the reverse test and the standard logistic regression test evaluate the same null hypothesis (Aschard *et al.* 2018). The reverse test has several advantages over the logistic regression approach. First, the reverse test approach generally uses less computation time, since the reverse test statistic has a closed form, in contrast to the iterative optimization procedure used in logistic regression. Simulation studies and some theoretical analysis that follows indicate that the reverse test tends to achieve a several- to 10-fold reduction in computation time compared to the logistic regression test, as the sample size becomes large. Second, in the reverse test approach, measurement error in the environmental exposure does not cause bias in the point estimates of the regression coefficients, because the measurement error is adsorbed in the error term. Third, the reverse test approach exhibits greater statistical power than the logistic regression approach, especially when the main effect of the exposure and the GxE interaction effect are large.

The reverse test in Aschard *et al.* (2018) did not consider confounders except for a brief discussion on this issue, whereas, as pointed out above, in GxE interaction studies it is usually necessary to control for a set of potential confounders. In this study, we extend the reverse test to adjust for confounders in the evaluation of the GxE interaction. Specifically, we introduce confounder terms into the linear regression model, which include not only the main effect of confounders but also the interaction effects between the confounders and gene/disease. In the scenarios where the confounder-environmental exposure relationship may be nonlinear, spline terms for the confounders can be incorporated in model. We perform simulation studies to evaluate the type I error rate and power of this test and compare it to the standard logistic regression approach. In an illustrative example, we apply the reverse test to the Veterans Aging Cohort Study (VACS) to investigate genetic variants modifying the association of smoking and HIV status.

## Materials and methods

### The logistic regression approach

In genome-wide association studies (GWAS), the logistic regression model has been commonly used to estimate the GxE interaction effects and test for their presence. This model has the following form

(1) $$\text{logit}(Pr(D=1|G,X,Z))=\alpha_{0}+m(Z)+\alpha_{x}X+\alpha_{g}G+\alpha_{gx}GX,$$

where $\text{logit}(\gamma )=\text{log}(\frac{\gamma }{1-\gamma })$ is the logit function, and *X*, *G*, *D*, and $Z={\left[Z_{1},\ldots ,Z_{p}\right]}^{T}$ denote a continuous exposure, a genetic variant, a binary disease status and possible confounders, respectively. We first consider a binary genetic variant, G={0,1}, to denote the risk allele noncarriers and carriers. The ordinal scenario for *G* will be considered at the end of this subsection. The confounders, *Z_j_* (j=1,…,p), can be continuous or binary. Here, $e^{\alpha_{x}}$, denoted as OR(D|X,G=0), is the odds ratio (OR) for the disease for a one unit increase in *X* at the reference genetic level *G *=* *0, $e^{\alpha_{g}}$, denoted as OR(D|G,X=0), is the OR for *G *=* *1 *vs* *G *=* *0 at the reference exposure level *X *=* *0. The parameter of interest is $e^{\alpha_{gx}}=\frac{\text{OR}(D|X,G=1)}{\text{OR}(D|X,G=0)}$, which is the ratio of the ORs (ROR) with respect to *X* for *G *=* *1 *vs* *G *=* *0, representing the effect of the GxE interaction. The null hypothesis, ROR=1, or equivalently $\alpha_{gx}=0$, indicates no interaction between the genetic variant and environmental exposure.

Without loss of generality, in the logistic regression model (1), we assume that the confounders term, m(Z), can be written as $\sum_{j=1}^{p}m_{j}(Z_{j})$. For binary *Z_j_*, $m_{j}(Z_{j})=\beta_{z_{j}}Z_{j}$. For continuous variable *Z_j_*, we may use the restricted cubic spline (Durrleman and Simon 1989) or other spline functions to model *Z_j_*. If using restricted cubic splines, we have $m_{j}(Z_{j})=\sum_{k=1}^{M-1}\alpha_{z_{j},k}B_{j,k}(Z_{j})$, where ${\left\{B_{j,k}(Z_{j})\right\}}_{k=1}^{M-1}$ is the spline basis based on *M* knots (Durrleman and Simon 1989). If *M *=* *2, $m_{j}(Z_{j})$ includes only a linear term, $\alpha_{z_{j},1}Z_{j}$. The knots are often placed at evenly spaced percentiles over the distribution of *Z_j_*. The confounder term, m(Z), can be rewritten as $\alpha_{z}^{T}\tilde{Z}$, where the vector $\tilde{Z}$ denotes all the terms generated by ***Z***, including all the possible spline basis, and $\alpha_{z}$ are the corresponding coefficients. Throughout this study, all vectors are column vectors. Generally, the maximum likelihood estimator of $\alpha ={\left[\alpha_{0},\alpha_{z}^{T},\alpha_{x},\alpha_{g},\alpha_{gx}\right]}^{T}$ can be obtained through an iteratively procedure. A 1-df wald test with respect to the null hypothesis, $H_{0}:\alpha_{gx}=0$, can be used to test the GxE interaction. The wald test statistic is $\tau_{\alpha }^{2}=\frac{{\hat{\alpha }}_{gx}^{2}}{\hat{\text{Var}}({\hat{\alpha }}_{gx})}$, which follows a 1-df $\chi^{2}$-distribution under the null.

In the above, Z-G or Z-X interaction effects have not been included in model (1). Vanderweele *et al.* (2013) and Keller (2014) pointed out that neglecting Z-G interaction terms could bias estimates of the GxE interaction and inflate the type I error of the null hypothesis. A similar argument applies to the Z-X interaction terms. Therefore, the Z-G and Z-X interaction terms must also be included in order to obtain valid GxE interaction estimates and tests. In model (1), it is straightforward to control for all the main effects and interaction effects of ***Z*** by replacing Z in the term m(Z) with $Z^{*}={\left[Z^{T},{\left(ZX\right)}^{T},{\left(ZG\right)}^{T}\right]}^{T}$. Inclusion of these terms will not change the estimation and test procedure for the GxE interaction effect.

The genetic variant, *G*, may also be treated as an ordinal variable with values 0, 1, and 2, corresponding to wild type, heterozygous genotype, and homozygous genotype, respectively. Under this scenario, $e^{\alpha_{g}}$ now represents the OR for one unit increase in *G* at the exposure reference level *X *=* *0, and $e^{\alpha_{gx}}$, the ROR, represents the ratio of ORs in *X* for one unit increase of *G*. The parameter estimation and testing for $H_{0}:\alpha_{gx}=0$, or equivalently $H_{0}:\text{ROR}=1$, are similar to those in the binary genetic variant scenario.

### The reverse test approach

Aschard *et al.* (2018) proposed a reverse test that exchanges the roles of the disease outcome *D* and continuous exposure *X*. Now, taking into account potential confounders, ***Z***, we assume *X*, conditional on *D*, *G*, and ***Z***, follows a normal distribution with constant variance; *i.e.*,

(2) $$X=\beta_{0}+h(Z)+\beta_{g}G+\beta_{d}D+\beta_{gd}GD+\epsilon ,$$

where $\epsilon ∼N(0,\sigma^{2})$. Similar to m(Z) in the logistic regression model, h(Z) can be written as $\sum_{j=1}^{p}h_{j}(Z_{j})$ with each of $h_{j}(Z_{j})$ a linear form $\beta_{z_{j}}Z_{j}$ or a spline function. Estimation of the unknown parameters in (2), denoted by β, can use ordinary least squares (OLS). Unlike with the logistic regression model (1), the parameter estimators for this linear regression model have a closed form. Thus, this linear regression approach is computationally more efficient than the logistic regression approach.

Now, we calculate the ROR based on the linear regression model (2). For notational simplicity, we assume the genetic variant is binary. The approach for ordinal genetic variants will be similar. In Appendix A, we show that $\text{OR}(D|X,G=g)=e^{(\beta_{d}+\beta_{gd}g)/\sigma^{2}}$ for *g *=* *0, 1. Therefore, $\text{ROR}=e^{\beta_{gd}/\sigma^{2}}$, and testing $H_{0}:\text{ROR}=1$ is equivalent to testing $H_{0}:\beta_{gd}=0$ in linear model (2). Also, using the linear regression model, the ROR can be estimated by $\hat{\text{ROR}}=\text{exp}({\hat{\beta }}_{gd}/{\hat{\sigma }}^{2})$, where ${\hat{\beta }}_{gd}$ and ${\hat{\sigma }}^{2}$ are the OLS estimates of *β_gd_* and the estimated variance of the error term, $\sigma^{2}$. The analytic formula for the standard error and estimated confidence interval of $\hat{\text{ROR}}$ are shown in Appendix B. In the linear model, we can also control for possible interaction effects between confounders and gene/disease by replacing ***Z*** in the term h(Z) with $Z^{**}={\left[Z^{T},{\left(ZD\right)}^{T},{\left(ZG\right)}^{T}\right]}^{T}$. In this linear model including the additional interactions, $e^{\beta_{gd}/\sigma^{2}}$ also represents the ROR (see Supplementary MaterialAppendix A for more detail); therefore, testing $\beta_{gd}=0$ is also valid for testing ROR=1. Similar to the logistic regression test, the reverse test can uses a 1-df wald test statistic $\tau_{\beta }^{2}=\frac{{\hat{\beta }}_{gd}^{2}}{\hat{\text{Var}}({\hat{\beta }}_{gd})}$ to check for the null hypothesis $H_{0}:\beta_{gd}=0$. One of major advantages of the logistic regression approach is that it can provide valid statistical inference if the data are a case-control study that is retrospectively sampled from a source population with known disease status. In Appendix C, we prove that the reverse test is also a valid approach under such a case-control sampling design.

Consider the following linear model with Z-*G* interaction terms,

(3) $$\{\begin{array}{cc} X=\beta_{0}+\tilde{h}(Z)+\overset{\approx }{h}(Z)G+\beta_{g}G+\beta_{d}D+\beta_{gd}GD+\epsilon , & \text{if}\;G\;\text{is}\;\text{binary},\\ X=\beta_{0}+\tilde{h}(Z)+\overset{\approx }{h}(Z)G+\overset{∼\approx }{h}(Z)G^{2}+\beta_{g}G+\beta_{d}D+\beta_{gd}GD+\epsilon , & \text{if}\;G\;\text{is}\;\text{ordinal}, \end{array}$$

where $\tilde{h}(Z),\;\overset{\approx }{h}(Z)$ and $\overset{∼\approx }{h}(Z)$ are functions of Z and $\epsilon ∼N(0,\sigma^{2})$ as for (2). Note that model (2) is a special case of (3). As shown in Supplementary MaterialAppendix B, the logistic model (1) and linear model (3) can hold simultaneously, and if both models hold, we can develop the following parametric relationships between the logistic regression and linear models: $\alpha_{x}=\frac{\beta_{d}}{\sigma^{2}}$ and $\alpha_{gx}=\frac{\beta_{gd}}{\sigma^{2}}$. Otherwise, however, the logistic regression model (1) and linear model (2) cannot hold simultaneously. In Supplementary MaterialAppendix B, we show that models (1) and (2) can also hold simultaneously and the parametric relationships above still hold. This occurs under the following two conditions. First, the error term in the linear model (2) is normally distributed with a constant variance. Second, $\text{logit}(Pr(D=1|G,Z))=b_{0}+b_{1}G+b_{2}G^{2}+b_{z}(Z)+b_{zg}(Z,G)$; where *G* can be binary or ordinal, and *b*_0_, *b*_1_, *b*_2_, $b_{z}(Z)$, and $b_{zg}(Z,G)$ are functions of the unknown parameters in models (1) and (2), and the definitions of *b’*s are shown in Supplementary Material Equation (S2).

The linear regression model (2) mainly relies on two assumptions: (i) the conditional normality assumption; *i.e.*, the error term in the linear model follows a normal distribution and (ii) the constant variance assumption; *i.e.*, $\text{Var}(\epsilon )=\sigma^{2}$ is constant. A normality test, such as the Jarque-Bera test (Jarque and Bera 1987) or the Shapiro-Wilk test (Shapiro and Wilk 1965), can be used to check whether regression residuals, *ϵ*, satisfy the conditional normality assumption. If necessary, a transformation on *X* can be applied before performing the GxE analysis using the reverse test approach. Commonly used transformations include logarithm, square root, Box-Cox (Box and Cox 1964), and Yeo-Johnson transformations (Yeo and Johnson 2000).

The constant variance assumption can be evaluated by implementing Levene’s test (Levene 1961) or the White test (White 1980) on the linear regression model’s residuals. When the constant variance assumption does not hold, *i.e.*, Var(ϵ) depends on ***Z***, *D*, and *G*, the OLS estimator of *β_gd_* will be consistent but the variance estimator of ${\hat{\beta }}_{gd}$ is invalid, often inflating the type I error for $H_{0}:\beta_{gd}=0$. Under heteroskedasticity, the sandwich variance should be used for $\hat{\text{Var}}({\hat{\beta }}_{gd})$ in $\tau_{\beta }^{2}={\left(\frac{{\hat{\beta }}_{gd}}{\sqrt{\hat{\text{Var}}({\hat{\beta }}_{gd})}}\right)}^{2}$. This $\tau_{\beta }^{2}$ statistic also follows a 1-df $\chi^{2}$-distribution under the null hypothesis. Sun *et al.* (2018) suggested that the sandwich estimator of variance may be biased downwards in finite samples and cause inflated type I error in hypothesis testing, and provided a bootstrap-based method, referred as BICS, to improve the performance of the $\tau^{2}$ statistic. It should be noted that, in the presence of heteroskedasticity, testing $H_{0}:\beta_{gd}=0$ is no longer equivalent to testing ROR=1. In the next section, we present alternative definitions of the GxE interaction and show that testing $H_{0}:\beta_{gd}=0$ is still a valid test for GxE interaction, even if the constant variance or the conditional normality assumption does not hold.

### Alternative definitions of the GxE interaction

In the previous two sections, the OR was used to represent the exposure-disease association; if the exposure-disease association differs in genetic subgroups, a GxE interaction was identified. In this section, we present several other parameters that also represent the exposure-disease association, leading to alternative definitions for GxE interactions.

The conditional mean difference of *X* between cases and controls (abbreviated as MD), *i.e.*, E(X|D=1,G,Z)−E(X|D=0,G,Z), can be used to represent the exposure-disease association. The difference of MD between the subgroups of *G *=* *1 and *G *=* *0, referred as the *difference of mean difference* (DMD), can represent the GxE interaction effect, and DMD=0 represents no GxE interaction (Aschard *et al.* 2018). In model (2), we can show the MDs at the genetic levels *G *=* *1 and *G *=* *0 are $\beta_{d}+\beta_{gd}$ and *β_d_*, respectively, and therefore $\text{DMD}=\beta_{gd}$. Noticing that the previous derivation of MD and DMD only requires that E(X|D,G,Z) agrees with what is presented in (2), it does not place any restriction on the distribution of the error term in linear model (2). In other words, even if *ϵ* in (2) is nonnormally distributed and heteroskedastic, $H_{0}:\beta_{gd}=0$ is valid for testing for no GxE interaction $H_{0}:\text{DMD}=0$.

Alternatively, Corr(X,D|G,Z) also measures the exposure-disease association, and the *difference in correlation coefficient* (DCC), defined as Corr(X,D|G=1,Z)−Corr(X,D|G=0,Z), also represents GxE interaction. If DCC=0, there is no exposure-disease correlation difference across genetic subgroups, and therefore no GxE interaction. In Supplementary MaterialAppendix C, we show that testing for $H_{0}:\text{DCC}=0$ is also valid through evaluating $H_{0}:\beta_{gd}=0$, under the assumptions of weak X-G and D-G associations, regardless of whether the normality assumption holds or not.

### Measurement errors in the environmental exposure

Many environmental exposures are measured with error, including data from self-administered questionnaires and laboratory measurements. In this section, we consider scenario under the classical additive measurement error model:

(4) $$X^{*}=X+\delta ,$$

where $X^{*}$ is the measurement of *X*, and *δ*, independent from *X*, *G*, ***Z***, and *D*, is the measurement error term following a mean zero normal distribution with variance $\sigma_{\delta }^{2}$. We can use the regression calibration method (Rosner *et al.* 1989, 1990) to obtain the corrected estimator for the interaction coefficient, ${\hat{\alpha }}_{gx}$, in the logistic regression model (1). Using the regression calibration approach, we fit the logistic regression model with *X* replaced by $X^{*}$:

(5) $$\text{logit}(Pr(D=1|G,X^{*},Z))=\alpha_{0}^{*}+m^{*}(Z)+\alpha_{x}^{*}X^{*}+\alpha_{g}^{*}G+\alpha_{gx}^{*}GX^{*}.$$

It follows that ${\hat{\alpha }}_{gx}\approx {\hat{\alpha }}_{gx}^{*}/\hat{\rho }$, where $\hat{\rho }=\frac{\hat{\text{Var}}(X)}{\hat{\text{Var}}(X)+{\hat{\sigma }}_{\delta }^{2}}$ and *ρ* represents the magnitude of the measurement error, when either (i) $Pr(D=1|X,X^{*},G,Z)=Pr(D=1|X,G,Z)$, the disease is rare, and X|G,Z is normal (Rosner *et al.* 1989); or (ii) $Pr(D=1|X,X^{*},G,Z)=Pr(D=1|X,G,Z)$ and $\text{Var}(X|X^{*},G,Z)$ is small (Carroll *et al.* 2006). Under either of the above conditions, testing $H_{0}:\alpha_{gx}^{*}=0$ in model (5) is also a valid test for no GxE interaction $H_{0}:\text{ROR}=1$, although the point estimator ${\hat{\alpha }}_{gx}^{*}$ is attenuated.

It is more straightforward to cope with the measurement errors in the linear regression model (2). Based on the assumption that *δ* is independent from *D*, *G*, and ***Z***, we can show that $E(X^{*}|G,D,Z)=E(X+\delta |G,D,Z)=E(X|G,D,Z)$ and $\text{Var}(X^{*}|G,D,Z)=\text{Var}(X+\delta |G,D,Z)=\sigma^{2}+\sigma_{\delta }^{2}$. Noting that both *δ* and *ϵ* are normally distributed, we have

(6) $$X^{*}=\beta_{0}^{*}+h^{*}(Z)+\beta_{g}^{*}G+\beta_{d}^{*}D+\beta_{gd}^{*}GD+\epsilon^{*},$$

where $\epsilon^{*}∼N(0,\sigma^{2}+\sigma_{\delta }^{2})$. All of the coefficients in the conditional mean (6) equal those in the original linear model (2), including $\beta_{gd}^{*}=\beta_{gd}$. Now, the parametric relationship $\beta_{gd}^{*}=\beta_{gd}$ holds exactly, in contrast to the approximation for logistic regression using the regression calibration approach. Because $\text{ROR}=e^{\beta_{gd}/\sigma^{2}}$, we have $\text{ROR}=e^{\beta_{gd}^{*}/\sigma^{2}}$, and testing $H_{0}:\beta_{gd}^{*}=0$ in linear regression model (6) is valid for testing $H_{0}:\text{ROR}=1$. The $\hat{\text{ROR}}=e^{{\hat{\beta }}_{gd}^{*}/{\hat{\sigma }}^{2}}$, however, will be attenuated, because ${\hat{\sigma }}^{2}$ tends to be overestimated as the variance of the error term, $\sigma^{2}+\sigma_{\delta }^{2}$, is larger than $\sigma^{2}$. If the measurement error term depends on *G*, *D*, or ***Z***, *i.e.*, $\text{Var}(\delta )=V(G,D,Z)\sigma_{\delta }^{2}$, where V(G,D,Z) is an unknown positive function, the linear model (6) with $\beta_{gd}^{*}=\beta_{gd}$ still holds. However, $\text{Var}(\epsilon^{*})=\sigma^{2}+V(G,D,Z)\sigma_{\delta }^{2}$ is no longer a constant. As discussed in the section above, in this heteroskedasticity scenario, we can use the sandwich variance in the test for $H_{0}:\beta_{gd}=0$.

### Simulation studies

To assess the validity of the reverse test approach and its power compared to the standard logistic regression model approach, we conducted simulation studies under a range of scenarios. We describe first the models considered in the simulation study, and then the data generation procedures.

Specifically, we consider the following logistic and linear regression model:

(7) $$\{\begin{array}{c} \text{logit}(Pr(D=1|G,X,Z))=\alpha_{0}+\alpha_{1}Z_{1}+\alpha_{2}Z_{1}^{2}+\alpha_{3}Z_{2}+\alpha_{g}G+\alpha_{x}X+\alpha_{gx}GX,\\ X=\beta_{0}+\beta_{1}Z_{1}+\beta_{2}Z_{1}^{2}+\beta_{3}Z_{2}+\beta_{g}G+\beta_{d}D+\beta_{gd}GD+\epsilon , \end{array}$$

where ϵ∼N(0,1), and *Z*_1_ and *Z*_2_ are potential confounders. A brief summary of the data generation process is shown as below and the detailed information can be found in Supplementary MaterialAppendix D. Specifically, we first generate *Z*_1_ and *Z*_2_, followed by the genetic variant *G*. Next the disease *D* is generated conditional on *Z*_1_, *Z*_2_, and *G*. Finally, the exposure *X* is generated based on the linear regression model in (7). Note that the logistic model in (7) also holds by this generation procedure, since the data generating process for $D|G,Z_{1},Z_{2}$ are carefully manipulated such that the distribution $D|X,G,Z_{1},Z_{2}$ coincides with the linear model in (7). We now specify the parameter values in (7). Noting that $\alpha_{0}=\text{log}(\frac{Pr(D=1|G=X=Z_{1}=Z_{2}=0)}{1-Pr(D=1|G=X=Z_{1}=Z_{2}=0)})$, we choose the values of *α*_0_ such that the baseline disease prevalence ranged from 5% to 50%. In the logistic regression model, we set $e^{\alpha_{1}},\;e^{\alpha_{2}}$ and $e^{\alpha_{3}}$ as 1.2, and specified *α_x_*, *α_g_* and *α_gx_* over realistic values such that OR(D|X,G=0)≤1.5, OR(D|G,X=0)≤1.5 and ROR≤1.5, each corresponding to a one unit increase in *X* and *G*. In the linear regression model, we fixed $\beta_{0}=0,\;\beta_{1}=0.03$ and $\beta_{2}=0.03$, and defined *β*_3_ and *β_g_* for a series of correlations between the outcome *X* and independent variables *G*, *D* and ***Z***, with $\beta_{3}=\frac{\text{Corr}(X,Z_{2}|Z_{1}=G=D=0)\sqrt{\text{Var}(X|Z_{1}=G=D=0)}}{\sqrt{\text{Var}(Z_{2}|Z_{1}=G=D=0)}}$, and $\beta_{g}=\frac{\text{Corr}(X,G|Z_{1}=Z_{2}=D=0)\sqrt{\text{Var}(X|Z_{1}=Z_{2}=D=0)}}{\sqrt{\text{Var}(G|Z_{1}=Z_{2}=D=0)}}$, where $\text{Corr}(X,Z_{2}|Z_{1}=G=D=0)$ and $\text{Corr}(X,G|Z_{1}=Z_{2}=D=0)$ were set at a range from 0.01 to 0.2, the conditional variances of *X* were specified as 1, and the conditional variances of *G* and *Z*_2_ were set to their corresponding unconditional variances. We specified $\beta_{d}=\alpha_{x}$ and $\beta_{gd}=\alpha_{gx}$, noting that $\beta_{d}=\alpha_{x}\sigma^{2},\;\beta_{gd}=\alpha_{gx}\sigma^{2}$, and $\sigma^{2}=1$.

We first simulated a cohort study with 100,000 subjects using the parameters and data generation procedure as above, and then randomly selected 1000 cases and 1000 controls from the cohort to create a 1:1 matched case-control study. Then, we fit the logistic model and linear model to obtain ${\hat{\alpha }}_{gx}$ and ${\hat{\beta }}_{gd}$, where a cubic B-spline approach with 3 interior knots was used for *Z*_1_ in each model. Next, we tested $H_{0}:\alpha_{gx}=0$ and $H_{0}:\beta_{gx}=0$ based on a 1-df wald test. We repeated the above procedure 500,000 times to evaluate the validity and statistical power of the two tests. Under measurement error scenarios, we generated $X^{*}$ based on (4), where the measurement error was set to explain 25–75% of the variance of $X^{*}$; that is, $\rho =\frac{\text{Var}(X)}{\text{Var}(X)+\sigma_{\delta }^{2}}$ from 75 to 25%. Here, ${\hat{\alpha }}_{gx}^{*}$ and ${\hat{\beta }}_{gd}^{*}$ were estimated from $X^{*}$ instead of *X* in two models.

In addition, we compared performance of the two tests when models (1) and (2) do not hold simultaneously. Specifically, we considered two scenarios, corresponding to the two conditions that are required for both models hold simultaneously. In Scenario I, we used a simple logistic regression model with probability $\text{logit}(P(D=1|G,Z))=\alpha_{0}+\alpha_{1}Z_{1}+\alpha_{2}Z_{1}^{2}+\alpha_{3}Z_{2}^{2}+\alpha_{g}G$ to generate the disease outcome, instead of the more complex expression given above to generate *D* in order to ensure both models hold simultaneously. In Scenario II, we considered scenarios where error term in the linear model does not follow normality and set the error term to follow a rectified Gaussian distribution (Socci *et al.* 1998), a right-skewed distribution that resets the negative elements of a normal distribution to 0. In each of these scenarios, we considered two data generation procedures, referred as the *logistic data generation procedure* and *linear data generation procedure*, corresponding to the following two cases: (i) a correctly specified logistic model but a misspecified linear model and (ii) a correctly specified linear model but a misspecified logistic model. More details for the simulation setting-ups in Scenarios I and II are deferred to Supplementary MaterialAppendix E and F, respectively.

### Illustrative example: the VACS

We illustrated the utility of the proposed reverse test by investigating the genome-wide interaction of gene-smoking on HIV status from the VACS. VACS is a multi-center, longitudinal observational study of HIV-infected and -uninfected veterans, whose primary objective is to understand the risk of substance abuse in subjects with HIV infection (Justice *et al.* 2006; Wu *et al.* 2019). As the GxE interaction effects may vary among different ethnic groups, we focused on the subgroup of African Americans in this example. The environmental exposure, smoking, was measured by cigarettes per day (≥0) in patient surveys collected at six clinic visits. Previous literature shows that cigarette smoking is a potential risk factor for HIV acquisition as it may be associated with high-risk sexual behavior (Burns *et al.* 1991; Marshall *et al.* 2009). For each subject, we defined smoking (cigarettes per day) as the average of the smoking data over the six clinic visits. The distribution of smoking is highly right-skewed with the coefficient of skewness 3.54 (Supplementary Figure S2A). To alleviate the skewness, a Yeo-Johnson transformation (Yeo and Johnson 2000) was applied. Specifically, the transformed smoking value is $T(x)=\frac{{\left(x+1\right)}^{\gamma }-1}{\gamma }$, where *x* is the original smoking values and *γ* is a tuning parameter which equals γ=−0.074 here. Because *γ* is close to 0, we can make the following approximation by L’Hopital’s rule: $T(x)\approx {\left(x+1\right)}^{\gamma }\times \text{log}(x+1)\approx \;\text{log}(x+1)$. Therefore, approximately, we can interpret the transformed smoking values as the logarithm of one plus the number of cigarettes per day. The density plots for the transformed exposure are shown in Supplementary Figure S2B, which presents that the transformed smoking variable is barely skewed, with the coefficient of skewness 0.03. In the reverse test, we used the transformed smoking varible as the response. We also used the transformed smoking variable in the logistic regression model.

All samples were genotyped on the Illumina OmniExpress BeadChip, and then imputed using IMPUTE2 (Howie *et al.* 2009) with the 1000 Genomes Phase 3 data as a reference panel, which resulted in a total of 17,092,657 SNPs. In this application study, we excluded subjects whose environmental exposure or HIV status were unavailable, and among that, the proportion of successfully imputed SNPs < 95%. Thus, after data cleaning, 1484 subjects were retained in the analysis, with 1403 males and 81 females, of whom 965 were HIV positive and 519 were HIV negative. The characteristics of the 1484 subjects stratified by the HIV status are provided in Supplementary Table S1, where the HIV negative group has lower proportions of males and smokers, whereas other characteristics are balanced between the HIV positive and negative groups. SNPs with minor allele frequency (MAF) >1% and call rate >95% were included in the analysis, which resulted in 10,079,672 SNPs for analysis of their interaction effects with smoking on the HIV infection. As for the confounders in the logistic and linear model, we considered age at baseline (continuous), and gender, as well as top 10 Principal Components of genotypes to control for population structure. We also consider alcohol intake (binary, drinkers *vs* nondrinkers) as a potential confounder in the smoking-HIV association because evidence shows that alcohol consumption may be associated with cigarette smoking and is also a potential risk factor for the incidence of HIV (Shuper *et al.* 2010). In the analysis, strong nonlinear main effects of age at baseline were observed. Thus, we used the cubic spline with 3 interior knots to adjust for the main effect of age in both models. We also included age-by-smoking and age-by-gene interactions in the logistic model to control for possible interaction effects between age at baseline and smoking/gene on disease outcome. Similarly, age-by-gene and age-by-HIV interaction terms were included in our reverse test.

## Results

### Type I error

We evaluated the validity of the reverse approach and logistic regression approach for testing GxE interaction by calculating the nominal type I error rates at 5% and 0.01% significant levels. Table 1 presents the empirical type I error rates of these tests over a range of scenarios, with and without measurement error in *X* in the circumstance when both the logistic regression and linear models hold. The null models were simulated by setting $\alpha_{gx}=\text{log}(\text{ROR})=0$ in the data simulation procedure. As shown in Table 1, type I error rates were close to the nominal P-value thresholds over the full range of design parameters we studied for both tests, regardless of whether there was a measurement error.

**Table 1:** Type I error rates of the reverse test and logistic regression test (with *ROR* = 1, *i.e.*, no GxE interaction effect)

$P_{0}(D=1)$	OR(D|G,X=0)	${\text{Corr}}_{0}(X,Z_{2})$	${\text{Corr}}_{0}(X,G)$	OR(D|X,G=0) =1.1				OR(D|X,G=0) =1.5			
				*X*		$X^{*}(\rho =0.25)$		*X*		$X^{*}(\rho =0.25)$	
				Logistic	Reverse	Logistic	Reverse	Logistic	Reverse	Logistic	Reverse
Simulation under 5% type I error threshold											
0.05	1.1	0.01	0.01	0.050	0.050	0.05	0.050	0.050	0.050	0.050	0.050
			0.2	0.050	0.050	0.05	0.050	**0.049**	0.050	0.050	0.049
		0.2	0.01	0.050	0.050	0.05	0.050	0.050	0.050	0.050	0.050
			0.2	0.050	0.050	0.05	0.049	0.050	0.050	0.050	0.050
	1.5	0.01	0.01	0.050	**0.051**	0.05	0.050	0.050	0.050	0.050	0.050
			0.2	0.050	0.050	0.05	0.050	0.050	0.050	0.050	0.050
		0.2	0.01	0.050	0.050	0.05	0.050	0.050	0.050	0.050	0.050
			0.2	0.050	0.050	0.05	0.050	0.050	0.050	0.050	0.051
0.20	1.1	0.01	0.01	0.050	0.050	0.05	0.050	0.050	0.050	0.050	0.050
			0.2	0.050	0.050	0.05	0.050	0.050	0.050	0.050	0.050
		0.2	0.01	0.051	0.050	0.05	0.050	0.050	0.050	**0.051**	0.050
			0.2	0.050	0.050	0.05	0.050	0.050	0.050	0.051	0.050
	1.5	0.01	0.01	0.050	0.050	0.05	0.050	0.050	0.050	0.050	0.050
			0.2	0.050	0.050	0.05	0.050	0.050	0.051	0.050	0.050
		0.2	0.01	0.050	0.050	0.05	0.050	**0.051**	0.051	0.050	**0.051**
			0.2	0.051	0.050	0.05	0.050	0.050	0.049	0.051	0.050
0.50	1.1	0.01	0.01	0.050	0.050	0.050	0.050	0.050	0.050	0.050	0.050
			0.2	0.050	0.049	0.050	0.050	0.050	0.050	0.050	0.050
		0.2	0.01	0.050	0.050	0.050	0.050	0.050	0.050	0.050	0.050
			0.2	0.050	0.050	0.050	0.050	0.050	**0.049**	0.050	0.050
	1.5	0.01	0.01	0.050	0.050	0.050	0.050	0.050	**0.049**	0.050	0.050
			0.2	0.050	0.050	0.050	0.050	0.050	0.050	0.050	0.050
		0.2	0.01	0.050	0.050	0.050	0.050	0.050	**0.049**	0.050	0.050
			0.2	0.051	0.051	0.050	0.050	0.050	0.050	0.050	0.050
Simulation under 0.01% type I error threshold											
0.05	1.1	0.01	0.01	9.8e-05	1.1e-04	1.0e-04	1.1e-04	8.8e-05	1.2e-04	7.8e-05	8.4e-05
			0.2	**5.8e-05**	9.0e-05	9.2e-05	8.8e-05	8.4e-05	8.4e-05	1.0e-04	1.2e-04
		0.2	0.01	7.8e-05	1.0e-04	7.4e-05	9.2e-05	7.6e-05	**1.5e-04**	**6.0e-05**	**7.0e-05**
			0.2	9.8e-05	9.8e-05	8.4e-05	1.1e-04	8.0e-05	9.8e-05	1.0e-04	**1.4e-04**
	1.5	0.01	0.01	8.2e-05	8.4e-05	9.8e-05	1.2e-04	1.1e-04	1.1e-04	8.6e-05	1.0e-04
			0.2	9.2e-05	1.2e-04	7.6e-05	1.1e-04	9.0e-05	8.4e-05	8.6e-05	1.0e-04
		0.2	0.01	1.0e-04	1.0e-04	8.0e-05	1.2e-04	1.0e-04	1.1e-04	9.0e-05	1.2e-04
			0.2	1.0e-04	8.6e-05	**5.8e-05**	**6.4e-05**	9.6e-05	1.1e-04	8.4e-05	8.0e-05
0.20	1.1	0.01	0.01	9.0e-05	1.2e-04	8.8e-05	1.2e-04	**7.2e-05**	8.4e-05	9.0e-05	1.1e-04
			0.2	9.4e-05	1.1e-04	7.8e-05	1.2e-04	1.2e-04	1.2e-04	1.0e-04	1.2e-04
		0.2	0.01	8.6e-05	9.6e-05	8.4e-05	1.0e-04	9.6e-05	1.1e-04	**7.0e-05**	1.1e-04
			0.2	9.6e-05	1.2e-04	8.2e-05	9.4e-05	8.6e-05	1.2e-04	7.4e-05	1.1e-04
	1.5	0.01	0.01	8.2e-05	7.8e-05	7.6e-05	9.0e-05	1.0e-04	9.8e-05	**7.2e-05**	8.6e-05
			0.2	8.6e-05	9.8e-05	**7.2e-05**	8.0e-05	7.8e-05	1.3e-04	1.1e-04	1.1e-04
		0.2	0.01	9.4e-05	9.2e-05	7.8e-05	1.0e-04	1.0e-04	1.2e-04	1.0e-04	1.2e-04
			0.2	8.0e-05	7.6e-05	8.2e-05	1.1e-04	8.4e-05	1.0e-04	9.2e-05	9.8e-05
0.50	1.1	0.01	0.01	**6.8e-05**	8.8e-05	7.8e-05	8.4e-05	9.0e-05	9.0e-05	**7.2e-05**	9.6e-05
			0.2	**6.0e-05**	7.4e-05	7.6e-05	1.0e-04	**6.2e-05**	9.6e-05	7.8e-05	1.0e-04
		0.2	0.01	8.2e-05	9.2e-05	7.8e-05	1.0e-04	1.0e-04	1.1e-04	1.2e-04	1.1e-04
			0.2	8.4e-05	1.0e-04	9.8e-05	1.2e-04	1.0e-04	1.0e-04	**6.8e-05**	9.0e-05
	1.5	0.01	0.01	8.2e-05	1.1e-04	8.2e-05	1.1e-04	8.0e-05	8.2e-05	**7.2e-05**	9.2e-05
			0.2	8.0e-05	9.2e-05	8.0e-05	9.0e-05	1.1e-04	1.0e-04	8.2e-05	1.1e-04
		0.2	0.01	8.8e-05	9.0e-05	1.0e-04	1.1e-04	1.0e-04	1.1e-04	8.2e-05	8.0e-05
			0.2	8.2e-05	9.6e-05	9.8e-05	1.1e-04	1.1e-04	1.1e-04	9.2e-05	1.2e-04

In this table, “logistic” and “reverse” represent the logistic regression test and reverse test respectively. $P_{0}(D=1),\;{\text{Corr}}_{0}(X,Z_{2})$, and ${\text{Corr}}_{0}(X,G)$ denote $Pr(D=1|Z_{1}=Z_{2}=G=D=0),\;\text{Corr}(X,Z_{2}|Z_{1}=G=D=0)$ and $\text{Corr}(X,G|Z_{1}=Z_{2}=G=D=0)$ respectively. Here, $\rho =\frac{\text{Var}(X)}{\text{Var}(X^{*})}$ denotes the magnitude of the measurement error, where *X* is the true exposure and $X^{*}$ is the observed exposure measured with error. The empirical type I error rates were calculated across 500,000 simulations for each scenario, where the empirical type I error rates outside the 95% confidence boundary, *i.e.*, $p\pm 1.96\times \sqrt{\frac{p(1-p)}{B}}$, were highlighted in bold. Here, *p* denotes the nominal threshold (5 or 0.01%) and *B* denotes the number of replication (500,000).

We evaluated the performance of both tests in Scenarios I and II where the logistic regression model (1) and linear model (2) do not hold simultaneously. Table 2 shows the type I error rates at a 0.01% significant level for both tests in Scenario I where P(D|G,Z) follows a simple logistic regression model. The reverse test approach had well-controlled type I error rates under both the logistic and the linear data generation procedures, whereas the logistic regression test exhibited very slight type I error deflation under the linear data generation procedure. At the 5% significant level, both approaches always presented satisfactory type I error rates when either the linear or the logistic regression model did not hold simultaneously (Supplementary Table S2).

**Table 2:** ** Type I error rates at 0.01% significant level of the reverse test and logistic regression test, in Scenario I that** P(D=1|G,Z) **follows a simple logistic regression model and the logistic regression model and the linear model do not hold simultaneously**

$P_{0}(D=1)$	OR(D|G,X=0)	${\text{Corr}}_{0}(X,Z_{2})$	${\text{Corr}}_{0}(X,G)$	OR(D|,X,G=0) =1.1				OR(D|X,G=0) =1.5			
				*X*		$X^{*}(\rho =0.25)$		*X*		$X^{*}(\rho =0.25)$	
				Logistic	Reverse	Logistic	Reverse	Logistic	Reverse	Logistic	Reverse
Linear data generation procedure											
0.05	1.1	0.01	0.01	7.8e-05	9.6e-05	**7.2e-05**	9.8e-05	**6.2e-05**	8.8e-05	7.8e-05	1.1e-04
			0.2	9.6e-05	1.1e-04	**6.6e-05**	1.0e-04	8.2e-05	1.0e-04	8.6e-05	8.2e-05
		0.2	0.01	9.6e-05	1.2e-04	8.6e-05	1.2e-04	8.4e-05	9.6e-05	8.4e-05	1.0e-04
			0.2	8.6e-05	7.4e-05	7.4e-05	1.1e-04	1.1e-04	1.2e-04	**7.2e-05**	9.2e-05
	1.5	0.01	0.01	**5.8e-05**	8.4e-05	**7.0e-05**	8.6e-05	9.4e-05	1.2e-04	9.0e-05	1.0e-04
			0.2	8.2e-05	8.8e-05	8.2e-05	**1.4e-04**	1.1e-04	**1.4e-04**	1.1e-04	1.2e-04
		0.2	0.01	7.8e-05	9.2e-05	**6.0e-05**	7.8e-05	9.8e-05	1.0e-04	8.2e-05	8.0e-05
			0.2	7.8e-05	1.0e-04	1.0e-04	1.0e-04	1.0e-04	1.1e-04	8.4e-05	8.6e-05
0.2	1.1	0.01	0.01	7.6e-05	1.0e-04	9.0e-05	1.1e-04	8.0e-05	1.0e-04	8.0e-05	9.0e-05
			0.2	1.1e-04	1.1e-04	9.0e-05	9.0e-05	1.0e-04	1.1e-04	1.1e-04	1.0e-04
		0.2	0.01	**7.2e-05**	1.2e-04	1.1e-04	1.1e-04	1.1e-04	1.0e-04	1.0e-04	1.1e-04
			0.2	7.8e-05	9.6e-05	**6.4e-05**	1.0e-04	**7.2e-05**	8.8e-05	8.4e-05	8.4e-05
	1.5	0.01	0.01	7.6e-05	9.6e-05	9.8e-05	**1.3e-04**	7.6e-05	9.6e-05	1.2e-04	**1.3e-04**
			0.2	7.8e-05	9.0e-05	8.6e-05	1.0e-04	8.4e-05	9.4e-05	7.6e-05	1.2e-04
		0.2	0.01	8.4e-05	9.0e-05	7.6e-05	8.8e-05	9.0e-05	9.8e-05	8.6e-05	1.1e-04
			0.2	7.8e-05	1.1e-04	**5.4e-05**	7.6e-05	8.2e-05	1.2e-04	9.8e-05	1.2e-04
0.5	1.1	0.01	0.01	1.1e-04	1.0e-04	**6.8e-05**	9.0e-05	**6.8e-05**	8.4e-05	**7.0e-05**	9.0e-05
			0.2	8.6e-05	1.0e-04	9.0e-05	9.4e-05	8.0e-05	1.2e-04	8.0e-05	9.6e-05
		0.2	0.01	8.0e-05	1.1e-04	8.0e-05	**7.0e-05**	8.4e-05	9.8e-05	**6.8e-05**	1.0e-04
			0.2	8.8e-05	9.6e-05	8.6e-05	8.8e-05	**7.0e-05**	1.1e-04	7.4e-05	9.0e-05
	1.5	0.01	0.01	7.8e-05	1.1e-04	7.4e-05	9.0e-05	8.6e-05	1.0e-04	8.6e-05	1.1e-04
			0.2	8.8e-05	9.0e-05	**7.2e-05**	9.6e-05	**6.0e-05**	1.1e-04	1.0e-04	1.0e-04
		0.2	0.01	8.2e-05	8.4e-05	1.0e-04	9.0e-05	8.2e-05	9.4e-05	8.0e-05	1.1e-04
			0.2	9.2e-05	1.1e-04	**5.4e-05**	8.4e-05	9.0e-05	1.0e-04	9.2e-05	1.0e-04
Logistic data generation procedure											
0.05	1.1	0.01	0.01	7.6e-05	9.4e-05	9.2e-05	1.0e-04	7.6e-05	1.0e-04	9.6e-05	9.8e-05
			0.2	8.2e-05	9.2e-05	8.0e-05	9.6e-05	8.2e-05	1.1e-04	7.6e-05	8.4e-05
		0.2	0.01	**7.2e-05**	1.1e-04	**6.2e-05**	7.8e-05	8.6e-05	1.1e-04	**6.6e-05**	9.8e-05
			0.2	7.6e-05	1.1e-04	8.8e-05	1.0e-04	7.6e-05	9.8e-05	8.4e-05	8.8e-05
	1.5	0.01	0.01	7.8e-05	1.1e-04	7.4e-05	8.4e-05	**6.0e-05**	9.0e-05	9.2e-05	**7.2e-05**
			0.2	9.4e-05	1.2e-04	8.8e-05	1.0e-04	7.8e-05	9.6e-05	8.2e-05	1.0e-04
		0.2	0.01	7.4e-05	9.6e-05	8.0e-05	1.1e-04	**6.2e-05**	9.2e-05	8.8e-05	1.1e-04
			0.2	9.0e-05	1.2e-04	1.1e-04	1.1e-04	8.0e-05	9.6e-05	1.0e-04	1.1e-04
0.2	1.1	0.01	0.01	8.0e-05	1.2e-04	7.4e-05	9.4e-05	9.8e-05	1.0e-04	7.4e-05	8.2e-05
			0.2	1.0e-04	1.2e-04	8.2e-05	9.4e-05	8.6e-05	1.0e-04	9.0e-05	**1.3e-04**
		0.2	0.01	7.6e-05	9.8e-05	7.6e-05	1.1e-04	8.6e-05	1.0e-04	8.0e-05	8.6e-05
			0.2	8.6e-05	1.1e-04	9.0e-05	9.2e-05	8.8e-05	1.0e-04	**5.2e-05**	9.4e-05
	1.5	0.01	0.01	8.8e-05	1.1e-04	8.8e-05	1.1e-04	**6.4e-05**	8.8e-05	8.2e-05	8.8e-05
			0.2	1.0e-04	1.0e-04	**7.0e-05**	9.4e-05	**5.8e-05**	**6.6e-05**	7.8e-05	**1.4e-04**
		0.2	0.01	**7.0e-05**	8.8e-05	7.6e-05	1.0e-04	9.2e-05	1.0e-04	1.1e-04	9.6e-05
			0.2	**6.8e-05**	1.0e-04	8.2e-05	1.0e-04	8.4e-05	1.1e-04	8.8e-05	1.2e-04
0.5	1.1	0.01	0.01	8.4e-05	9.0e-05	8.2e-05	1.0e-04	1.0e-04	8.8e-05	8.4e-05	9.4e-05
			0.2	9.4e-05	1.1e-04	7.4e-05	9.2e-05	7.6e-05	**1.4e-04**	8.2e-05	1.2e-04
		0.2	0.01	8.8e-05	1.1e-04	7.6e-05	9.2e-05	1.0e-04	1.0e-04	**6.8e-05**	8.4e-05
			0.2	9.4e-05	1.1e-04	8.0e-05	9.2e-05	7.6e-05	9.4e-05	**6.0e-05**	**6.4e-05**
	1.5	0.01	0.01	8.6e-05	1.0e-04	8.6e-05	1.0e-04	**6.0e-05**	9.2e-05	7.6e-05	1.1e-04
			0.2	9.0e-05	9.2e-05	8.0e-05	9.2e-05	**7.2e-05**	8.6e-05	9.8e-05	8.4e-05
		0.2	0.01	**7.2e-05**	1.0e-04	**7.2e-05**	**6.6e-05**	7.6e-05	8.4e-05	7.6e-05	8.6e-05
			0.2	8.8e-05	1.1e-04	8.6e-05	1.0e-04	8.8e-05	1.2e-04	8.4e-05	9.8e-05

In this table, “logistic” and “reverse” represent the logistic regression test and reverse test respectively. $P_{0}(D=1),\;{\text{Corr}}_{0}(X,Z_{2})$, and ${\text{Corr}}_{0}(X,G)$ denote $Pr(D=1|Z_{1}=Z_{2}=G=D=0),\;\text{Corr}(X,Z_{2}|Z_{1}=G=D=0)$ and $\text{Corr}(X,G|Z_{1}=Z_{2}=G=D=0)$ respectively. Here, $\rho =\frac{\text{Var}(X)}{\text{Var}(X^{*})}$ denotes the magnitude of the measurement error, where *X* is the true exposure and $X^{*}$ is the observed exposure measured with error. The empirical type I error rates were calculated across 500,000 simulations for each scenario, where the empirical type I error rates outside the 95% confidence boundary, *i.e.*, $p\pm 1.96\times \sqrt{\frac{p(1-p)}{B}}$, were highlighted in bold. Here, *p* denotes the significance level (0.01%) and *B* denotes number of replication (500,000).

Supplementary Tables S3 and S4 provided empirical type I error rates in Scenario II where the error term in the linear model follows a rectified Gaussian distribution. Under the linear data generation procedure, the reverse test exhibited no inflation or deflation at any of the nominal threshold considered; in contrast, the logistic regression test had inflated type I error under most settings considered, especially when the OR of *X* was large without measurement error. Under the logistic data generation procedure, the logistic regression test had well-controlled empirical type I error rates; for the reverse test, while the type I error rates of the reverse test were almost always below the nominal levels with occasionally a slight deflation.

### Power comparison

We compared the statistical power of the logistic regression test and reverse test by calculating the ratio $\frac{{\bar{\chi }}_{\text{rev}}^{2}}{{\bar{\chi }}_{\;\text{log}\;}^{2}}$, simply referring as $\chi^{2}$ ratio henceforth, where ${\bar{\chi }}_{\text{rev}}^{2}$ and ${\bar{\chi }}_{\;\text{log}\;}^{2}$ are the average $\chi^{2}$ test statistics of the reverse test and logistic regression test over 500,000 repetitions for each simulation study scenario. A $\chi^{2}$ ratio greater than 1 indicates that the reverse test obtained statistical power than the logistic regression test.

**Figure 1:**
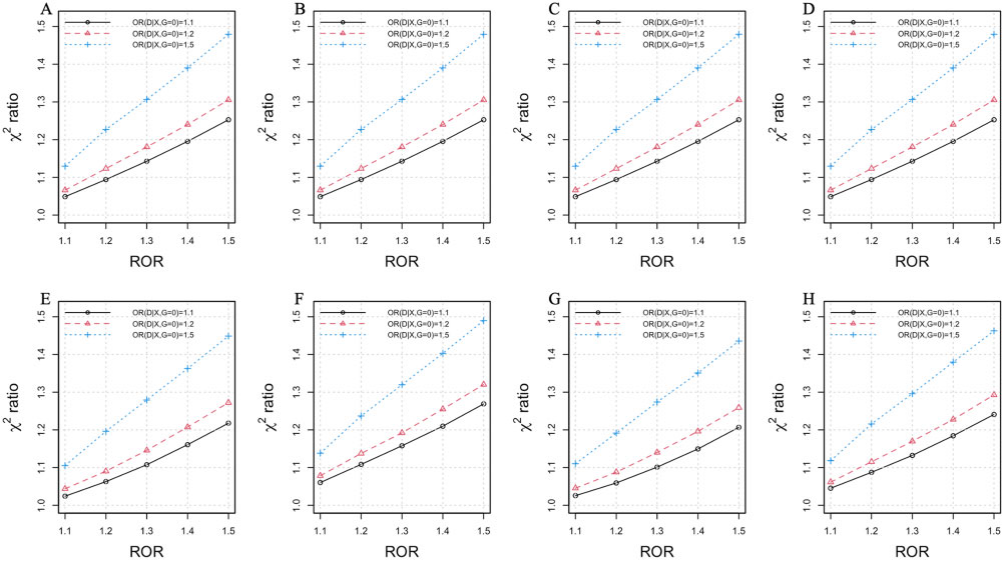
Power comparison between the reverse test and logistic regression test, where the *x*-axis is the ROR representing the magnitude of the GxE interaction, and the *y*-axis is the average $\chi^{2}$ ratio of the reverse test against the logistic regression test. The results were calculated through 500,000 simulations, with increasing the ROR and main effect of *X* (*i.e.*, OR(X|G=0)) from 1.1 to 1.5. Left column: weak *vs* strong exposure-confounder association, of $\text{Corr}(X,Z_{2}|Z_{1}=D=G=0)$ = 0.01 (A) and 0.2 (E); Second column: weak *vs* strong disease-gene association, OR(G|X=0)= 1.1 (B) or 1.5 (F); Third column: weak *vs* strong gene-exposure correlation, of $\text{Corr}(X,G|Z_{1}=Z_{2}=D=0)$=0.01 (C) or 0.2 (G); Right column: rare *vs* common disease prevalence, of $Pr(D=1|G=X=Z_{1}=Z_{2}=0)$=0.05 (D) or 0.2 (H).

Figure 1 provides the $\chi^{2}$ ratio of the reverse test against logistic regression test in the absence of measurement error in *X*. We considered the exposure main effects, *i.e.*, OR(D|X,G=0), from 1.1 to 1.5, and GxE effects, *i.e.*, ROR, from 1.1 to 1.5, while considering weak and strong exposure-confounder correlation ($\text{Corr}(X,Z_{2}|Z_{1}=G=D=0)=0.01$, 0.2), weak and strong genetic variant main effects (OR(D|G,X=0)=1.1, 1.5), rare and common disease prevalence ($Pr(D=1|G=X=Z_{1}=Z_{2}=0)$=0.05, 0.2), and weak and strong gene-exposure correlation ($\text{Corr}(X,G|Z_{1}=Z_{2}=D=0)$=0.01, 0.2). As shown in Figure 1, all the $\chi^{2}$ ratios were greater than 1, indicating the reverse test provided higher statistical power than the logistic regression test. The power advantage of the reverse test against logistic regression improved with the increase of the main effect of exposure; for example, the $\chi^{2}$ ratios were generally below 1.25 for OR(D|X,G=0)=1.1 and ROR=1.5 and were greater than 1.45 for OR(D|X,G=0)=1.5 and ROR=1.5. As the ROR increased, the reverse test also tended to have higher statistical power than the logistic regression test. However, we observed a slight decrease in the relative efficiency as the association between *X* and *Z*_2_ and the exposure-gene association increased. The disease prevalence and disease-gene association had minimal influence on the relative efficiency of the two tests.

More detailed results for the power comparison between the reverse and logistic regression test are provided in Table 3, in which we investigate the impact of exposure measurement error on the relative efficiency. Typically, the magnitude of the measurement error resulted in a decrease in the relative efficiency of the reverse test *vs* the logistic regression test, but the reverse test still provided a power advantage even when ρ=0.25. For example, when OR(D|X,G=0)=1.5, the $\chi^{2}$ ratio decreased from over 1.4 in the absence of measurement error (*ρ*  =  1) to below 1.15 for ρ=0.25. Supplementary Figure S5 presents the rejection rates (*i.e.*, power) under the alternative hypothesis that ROR = 1.5 across 50,000 replications for the logistic regression approach and the reverse test at significance levels of 0.01 and 5% when the logistic and linear regression models hold simultaneously. As expected, the rejection rate raises when measurement error decreases or significance level increases. The reverse test exhibits higher rejection rates under nearly all settings in the absence of measurement error and also provides power advantage under most simulation settings with a large measurement error (ρ=0.25).

**Table 3:** Relative power of the reverse test against logistic regression test, where ROR = 1.5

$P_{0}(D=1)$	OR(D|G,X=0)	${\text{Corr}}_{0}(X,Z_{2})$	${\text{Corr}}_{0}(X,G)$	$\chi^{2}$ ratio							
				OR(D|X,G=0) =1.1				OR(D|X,G=0) =1.5			
				*ρ *= 1	ρ=0.75	ρ=0.5	ρ=0.25	*ρ *= 1	ρ=0.75	ρ=0.5	ρ=0.25
0.05	1.1	0.01	0.01	1.25	1.19	1.14	1.08	1.48	1.36	1.25	1.14
			0.2	1.21	1.13	1.06	0.99	1.43	1.30	1.18	1.07
		0.2	0.01	1.22	1.15	1.08	1.02	1.45	1.32	1.20	1.09
			0.2	1.17	1.09	1.01	0.94	1.40	1.27	1.14	1.02
	1.5	0.01	0.01	1.27	1.21	1.16	1.10	1.49	1.38	1.27	1.16
			0.2	1.23	1.16	1.09	1.02	1.47	1.34	1.22	1.11
		0.2	0.01	1.23	1.17	1.11	1.04	1.46	1.34	1.22	1.12
			0.2	1.21	1.12	1.04	0.97	1.44	1.31	1.18	1.07
0.20	1.1	0.01	0.01	1.24	1.18	1.13	1.07	1.46	1.35	1.24	1.13
			0.2	1.19	1.12	1.05	0.98	1.42	1.29	1.17	1.06
		0.2	0.01	1.21	1.14	1.08	1.02	1.44	1.31	1.19	1.08
			0.2	1.17	1.09	1.01	0.94	1.39	1.26	1.14	1.02
	1.5	0.01	0.01	1.25	1.20	1.14	1.09	1.47	1.36	1.25	1.15
			0.2	1.22	1.15	1.08	1.02	1.44	1.32	1.20	1.10
		0.2	0.01	1.22	1.16	1.10	1.04	1.44	1.32	1.21	1.11
			0.2	1.19	1.12	1.04	0.97	1.42	1.29	1.17	1.06
0.50	1.1	0.01	0.01	1.23	1.17	1.12	1.06	1.45	1.34	1.23	1.12
			0.2	1.19	1.12	1.05	0.98	1.41	1.28	1.17	1.06
		0.2	0.01	1.20	1.14	1.07	1.01	1.43	1.30	1.19	1.08
			0.2	1.16	1.08	1.01	0.94	1.39	1.25	1.13	1.03
	1.5	0.01	0.01	1.24	1.19	1.14	1.08	1.45	1.34	1.24	1.14
			0.2	1.21	1.14	1.08	1.02	1.42	1.30	1.19	1.09
		0.2	0.01	1.21	1.15	1.09	1.03	1.42	1.31	1.20	1.10
			0.2	1.18	1.11	1.04	0.98	1.39	1.27	1.16	1.06

$P_{0}(D=1),\;{\text{Corr}}_{0}(X,Z_{2})$ , and ${\text{Corr}}_{0}(X,G)$ denote $Pr(D=1|Z_{1}=Z_{2}=G=D=0),\;\text{Corr}(X,Z_{2}|Z_{1}=G=D=0)$ and $\text{Corr}(X,G|Z_{1}=Z_{2}=G=D=0)$, respectively. Here, $\rho =\frac{\text{Var}(X)}{\text{Var}(X^{*})}$ denotes the magnitude of the measurement error, where *X* is the true exposure and $X^{*}$ is the observed exposure measured with error. The relative power was calculated by $\frac{{\bar{\chi }}_{\text{rev}}^{2}}{{\bar{\chi }}_{\;\text{log}\;}^{2}}$ where ${\bar{\chi }}_{\text{rev}}^{2}$ and ${\bar{\chi }}_{\text{rev}}^{2}$ are the average $\chi^{2}$ statistics for the logistic regression test and reverse test based on 500,000 simulation repetitions for each scenario.

We also investigated relative efficiency when the linear and logistic regression models did not hold simultaneously. The results for Scenario I are shown in Supplementary Table S6. As expected, the reverse test exhibited a significant power advantage under the linear model data generation procedure. Under the logistic data generation procedure, the reverse test still provided power advantage among 62.5% of the simulation scenarios under a weak D-X association (OR(D|X,G=0)=1.1) and 50%, when OR(D|X,G=0)=1.5. Among the simulation scenarios where $\chi^{2}$ ration <1, only 5 out of 84 (6.0%) had $\chi^{2}$ ratio below 0.9. Supplementary Table S7 presents the relative efficiency for Scenario II where the error term in the linear model followed a rectified Gaussian distribution. Under the linear data generation procedure, the reverse test generally outperformed the logistic regression test, the $\chi^{2}$ ratio was less than 1 in only 12 out of 192 (6.3%) simulation scenarios. Under the logistic data generation procedure, the reverse test still provided satisfactory results; the $\chi^{2}$ ratio was less than 0.9 in 49 out of 192 (25.6%) simulation scenarios. In addition, we also observed that the reverse test provided greater statistical power against the logistic regression test for small main effects of *X* and weak correlations between exposure and genetic variant.

### Computational time

The reverse test is more computationally efficient than the logistic regression test, since it is based on a closed form test statistics, in contrast to the iterative procedure used by the logistic regression test. Table 4 displays the computation time of the two tests using R software on a single desktop computer (3.7 GHz CPU and 16 GB RAM) for GxE analysis of 1,000,000 SNPs with the sample sizes from 1000 to 200,000. We can see that the reverse test outperformed the logistic regression test in all scenarios. Furthermore, as the sample size increased, the ratio of the computation time of the logistic regression test comparing to the reverse test increased. With a sample size of 200,000, an approximately 7.5-fold reduction in computation time was observed for the reverse test.

**Table 4:** Comparison of computation time

Sample size	Computation time in hours		
(Cases+Controls)	Reverse test	Logistic regression test	Ratio
1,000	0.34	1.19	3.54
2,000	0.44	2.10	4.78
10,000	2.76	20.64	7.47
40,000	5.47	41.18	7.53
200,000	30.79	236.58	7.68

We compared the computation time for the reverse and logistic regression test for GxE interaction, in hours for 1,000,000 SNPs.

### Illustrative example: gene-smoking interactions in VACS

**Figure 2:**
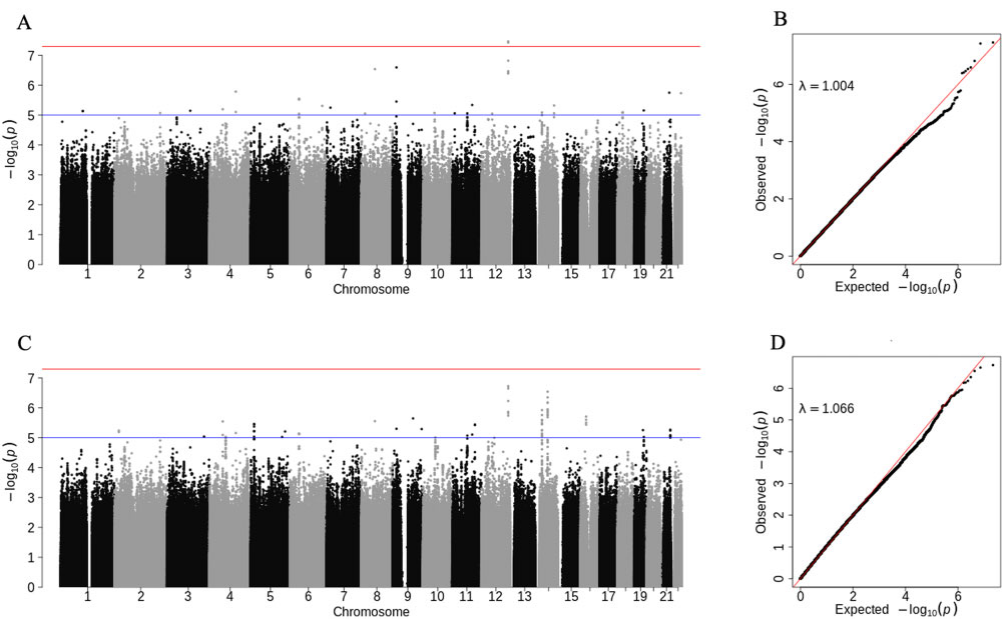
Manhattan plots and Quantile-Quantile plots for the test of interaction effect between 10,079,672 common SNPs and smoking (cigarettes per day) using the reverse test (upper panel) and standard logistic regression test (lower panel). In the Quantile-Quantile plots, *λ* denotes the genomic inflation factor. Red line: genome-wide significance level (*P*-value$=5\times 10^{-8}$). Blue line: suggestive level (*P*-value$=1\times 10^{-5}$).

Interaction effects between genetic variants and smoking in relation to HIV status was considered among 10,079,672 SNPs in 1484 African Americans in VACS, using both the reverse test and logistic regression approach, adjusting for age, sex, alcohol intake, and the top 10 Principal Components. Quantile–Quantile (Q–Q) plots and Manhattan plots for both tests are presented in Figure 2. The reverse test displayed no evidence of *P*-value inflation in the QQ plot (Figure 2B), with a genomic inflation factor, *λ*, close to 1, while the logistic regression test showed some evidence of inflation in the QQ plot (Figure 2D), with λ=1.07 We observed a strong correlation between the *P*-values of the logistic and reverse tests, with the correlation coefficient 0.82.

Table 5 presents the results for SNPs where at least one of the reverse and logistic regression tests provided a *P*-value below $1\times 10^{-6}$. We can see the top-ranked SNPs largely overlapped between the two approaches. No SNPs showed genome-wide significance (*P*-value $<5\times 10^{-8}$) for the interaction under the logistic regression approach. However, the reverse test presents that two SNPs, rs10744166 and rs10773060, have significant interaction effects (*P*-value = $3.71\times 10^{-8}$ and $3.42\times 10^{-8}$). Both SNPs are located at gene *ZNF664* in chromosome 12, which was previously identified to be associated with clubfoot (Zhang *et al.* 2014). The minor allele frequencies (MAFs) of the two SNPs were 45.6 and 45.4% in the study sample, and were slightly higher as compared to the 1000 Genomes (with MAF 37.5 and 37.4%, respectively). In order to illustrate interactions across genotypes, averages of the smoking values (cigarettes per day) between the HIV-infected and -uninfected veterans stratified by different genotypes of rs10744166 are visualized in Supplementary Figure S3A. As can be seen from the figure, an increase in the number of minor alleles of rs10744166 indicates a larger mean difference between the smoking values in the HIV-infected and -uninfected individuals, such that the mean difference for the genotype with two minor alleles is at nearly 3.5 times the mean difference for the genotype with no minor alleles. In other words, the minor allele in rs10744166 is associated with a stronger smoking-HIV infection relationship. A similar pattern between genotypes and mean differences were also found in rs10773060, as visualized in Supplementary Figure S3B. A linkage disequilibrium analysis shows evidence that rs10744166 and rs10773060, along with the other four SNPs located at *ZNF664* shown in Table 5, are in linkage disequilibria spanning a 1.5 kb length (Supplementary Figure S3C), where the squared allele frequency correlations *R*^2^ for each pair of SNPs are ranged between 0.95 and 0.99. It is of note that there was limited information on the detected SNPs given the small sample size of this illustrative example. Replication in an independent dataset is needed to confirm those associations.

**Table 5:** ** SNPs with *P*-value** $<1\times 10^{-6}$ **in at least one of the reverse and logistic regression test in the analysis gene-smoking interaction effects in relation to HIV infection (VACS African Americans, *n* = 1484)**

Chromosome	Gene	SNP name	Major allele	Minor allele	MAF	Logistic test	Reverse test
8	*AC018953.1*	rs72649207	C	G	0.08	2.79e-06	**2.88e-07**
9	*SH3GL2*	rs77236711	G	A	0.05	5.01e-06	**2.54e-07**
12	*ZNF664*	rs10773059	T	C	0.44	1.36e-06	**3.40e-07**
		rs10744166	T	C	0.46	2.25e-07	**3.71e-08**
		rs10773060	G	A	0.45	1.88e-07	**3.42e-08**
		rs10744167	C	T	0.44	5.87e-07	**1.51e-07**
		rs7315555	G	A	0.45	1.52e-06	**3.84e-07**
		rs7303161	T	C	0.45	1.82e-06	**4.03e-07**
14	*SLC35F4*	rs10145503	G	A	0.25	**4.47e-07**	4.08e-05
		rs10148287	G	A	0.25	**2.87e-07**	3.09e-05
		rs12432123	G	A	0.27	**6.60e-07**	4.46e-05
		rs7146231	T	C	0.27	**6.74e-07**	4.66e-05

The smaller *P*-value between the two tests was highlighted in bold.

We further performed a separate analysis for gene-smoking interactions in the subgroup of smokers (*n* = 905). The QQ and Manhattan plots of both tests are visualized in Supplementary Figure S4. We observed no SNPs exhibiting genome-wide significance based on either logistic regression test or reverse test. This is not surprising as the sample size is relatively small. Although rs10744166 and rs10773060 show genome-wide significance previously, both SNPs do not appear to be significant in the smokers subgroup by either the reverse test (*P*-value = 0.08 and 0.09) and the logistic regression test (*P*-value = 0.02 and 0.02).

## Discussion

In this study, we propose a reverse test for interaction between an environmental exposure and a genetic variant on a binary disease status, adjusting for confounding. Comparing with the standard logistic regression test for interaction with a continuous or binary environmental exposure, the reverse test only applies to a continuous environmental exposure. This reverse test leverages the spirit of linear discriminant analysis by reversing the roles of the environmental exposure and disease status, and obtains a closed form of the GxE test statistic. Our analysis shows that when the error term in the linear model follows a normal distribution with constant variance, both the reverse and standard logistic regression approaches are valid for testing $H_{0}:\mathrm{ROR}=1$. Compared to the logistic regression approach, the reverse test has a larger statistical power. As a trade-off, the reverse test approach additionally assumes that the exposure is continuous. The logistic regression approach has a wider range of applications, in which the exposure can be continuous, binary, count, and categorical variables.

The reverse approach can be extended to test for interactions between a genetic marker set and a continuous environmental exposure with a binary disease outcome. The standard approach is based upon the logistic regression model $\text{logit}[Pr(D=1|X,G,Z)]=\alpha_{0}+\alpha_{z}^{T}Z+\alpha_{g}^{T}G+\alpha_{x}X+\alpha_{gx}^{T}S$, where $G={\left[G_{1},G_{2},\ldots ,G_{p}\right]}^{T}$ denotes a genetic marker set containing *p* genetic variants and $S={\left[G_{1}X,G_{2}X,\ldots ,G_{p}X\right]}^{T}$ denotes the interaction terms between the genetic marker set and exposure. The null hypothesis for the interaction is $H_{0}:\alpha_{gx}=0$. Following reverse approach, we assume a linear model for *X*, $X=\beta_{0}+\beta_{z}^{T}Z+\beta_{g}^{T}G+\beta_{d}D+\beta_{gd}^{T}U+\epsilon$, where $\epsilon ∼N(0,\sigma^{2})$ and $U={\left[G_{1}D,G_{2}D,\ldots ,G_{p}D\right]}^{T}$ is the interaction terms between the genetic set and disease status. We can also build a parametric relationship such that $\alpha_{gx}=\frac{\beta_{gd}}{\sigma^{2}}$. Then, testing for $H_{0}:\alpha_{gx}=0$ in the logistic model can be evaluated through testing for $H_{0}:\beta_{gd}=0$ in the linear model. Based on the theory of linear discriminant analysis (Efron 1975), it is expected that the reverse approach outperforms the reverse test with respect to the statistical power if linear model is correctly specified and the error term follows a homoskedastic normal distribution.

We conducted simulation studies across a wide range of scenarios to evaluate the performance of the proposed approach. Several observations followed from our simulation experiments. First, the reverse test produces correct type I error rates, both for standard or very small *P*-value threshold of 5 and 0.01%, whether or not there is exposure measurement error. Second, the reverse test generally exhibited greater statistical power than the standard logistic approach, and its relative statistical power improved when the magnitude of the GxE interaction effect or the main effect of exposure increased. Although measurement error in the exposure tended to diminish the power advantage of the reverse test, the reverse test still provided some power gain even under very severe measurement error of ρ=0.25. Third, the reverse test is substantially more computationally efficient. It achieves a computation time that is more than sevenfold less than that of the logistic regression test, a great advantage in coping with large-scale genomic studies with millions of SNPs. Fourth, the proposed approach performed reasonably well when the error term is not normal. In summary, the reverse test provided a valid, powerful, and computationally efficient alternative for investigating GxE interactions in large-scale genomic research.

Does the relative computational advantage of the reverse test change as sample size increases? In fact, the ratio of the computation time of the logistic regression test and the reverse test is likely bounded by a constant when the sample size is sufficiently large. Specifically, fitting a linear model with an OLS algorithm has $O(np^{2})$ computational complexity in general (Drineas *et al.* 2011; Iyer 2015), where *n* is the sample size and *p* is the number of unknown coefficients . The computational complexity of logistic regression model depends on the optimization algorithm used. One popular algorithm is iteratively reweighted least squares (IRLS), which is also the default algorithm for fitting generalized linear models in many statistical softwares (such as R). As an iterative algorithm, the IRLS solves a weighted least squares (WLS) subproblem at each iteration, and the running time of this WLS subproblem is comparable with the OLS algorithm if they share same number of sample size and unknown coefficients (see Supplementary MaterialAppendix G for more details). In other words, the additional computational burden of the logistic regression test compared to the reverse approach mainly depends on the number of the IRLS iterations, when both tests adjusted for same number of covariates. Although the number of IRLS iterations is affected by initial values, as discussed in Komarek (2004), it is typically around 5 or 10 in most of the scenarios and barely larger than 30. It follows that the computation time of the logistic regression test based on IRLS tends to be several to 10 times longer than the reverse test when the sample size is sufficient large. These sorts of gains in computational efficiency can become quite important when whole-genome scanning, involving 6.4 billion SNPs, are to be undertaken.

In conclusion, given its power advantages and substantial benefits in computing time, the reverse test can be quite a useful tool in investigating GxE interactions, permitting whole-genome scans over many exposures simultaneously, measured with or without measurement error.

## Supplementary Material

jkab236_Supplementary_Data

## Data Availability

The authors state that all data necessary for confirming the conclusions of the article are present within the article. A tutorial for implementing the proposed methods in R software is available at https://github.com/chaochengstat/GxE2020. The R codes for replicating the simulation studies and analysis of the VACS illustrative example are available at https://github.com/chaochengstat/GxE2020/tree/main/Rcode_Genetics. Supplementary Material for this manuscript is attached at the end of the article, which includes Supplementary MaterialAppendix A–G, Supplementary Figures S1–S3, and Supplementary Tables S1–S7. Supplementary material is available at *G3* online.

## Appendix

### Appendix A: Derivation of OR (D|X,G=g) based on the linear regression model

First, by definition,

$$\text{OR}(D|X,G=g,\{\{Z=z)\}\}=\frac{Pr(D=1|X=x+1,G=g,Z=z)/Pr(D=0|X=x+1,G=g,Z=z)}{Pr(D=1|X=x,G=g,Z=z)/Pr(D=0|X=x,G=g,Z=z)},\;g=0,1.$$

Using the Bayes’ theorem,

Pr(D|X,G=g,Z=z)=f(X|D,G=g,Z=z)Pr(D|G=g,Z=z)/f(X|G=g,Z=z),

where f(.) denotes the probability density functions (p.d.f’s). It follows that

(8) $$\text{OR}(D|X,G=g,Z=z)\{\{=\frac{f(X=x+1|D=1,G=g,Z=z)/f(X=x|D=1,G=g,Z=z)}{f(X=x+1|D=0,G=g,Z=z)/f(X=x|D=0,G=g,Z=z)}.$$

Since X|D,G,Z follows a normal distribution with mean E(X|D,G,Z) and variance $\sigma^{2}$, we have

$$\begin{array}{cc} \text{OR}(D|X,G=g,Z=z)=\frac{\frac{1}{\sqrt{2\pi }\sigma }e^{-\frac{{\left(x+1-(\beta_{0}+h(z)+\beta_{g}g+\beta_{d}+\beta_{gd}g)\right)}^{2}}{2\sigma^{2}}}/\{\frac{1}{\sqrt{2\pi }\sigma }e^{-\frac{{\left(x-(\beta_{0}+h(z)+\beta_{g}g+\beta_{d}+\beta_{gd}g)\right)}^{2}}{2\sigma^{2}}}\}}{\frac{1}{\sqrt{2\pi }\sigma }e^{-\frac{{\left(x+1-(\beta_{0}+h(z)+\beta_{g}g)\right)}^{2}}{2\sigma^{2}}}/\{\frac{1}{\sqrt{2\pi }\sigma }e^{-\frac{{\left(x-(\beta_{0}+h(z)+\beta_{g}g)\right)}^{2}}{2\sigma^{2}}}\}}\\ & =e^{(\beta_{d}+\beta_{gd}g)/\sigma^{2}}. \end{array}$$

As result, $\text{ROR}=\frac{\text{OR}(D|X,G=1,Z=z)}{\text{OR}(D|X,G=0,Z=z)}=e^{\frac{\beta_{gd}}{\sigma^{2}}}.$

### Appendix B: Inference about the ROR based on linear model (2)

From Appendix A and linear model (2), the log(ROR) can be estimated by ${\hat{\beta }}_{gd}/{\hat{\sigma }}^{2}$, where ${\hat{\beta }}_{gd}$ is obtained by the standard OLS theory and ${\hat{\sigma }}^{2}$ by the empirical variance of the residuals in model (2). We now use subscript *i* to index the *i*th subject. Specifically, $h(Z_{i})$ can be represented as ${\tilde{Z}}_{i}^{T}\beta_{z}$, where ${\tilde{Z}}_{i}$ is a vector containing all the variables generated from $Z_{i}$, including all spline terms. With *n* subjects, linear model (2) can be rewritten as X=Mβ+ϵ, where ***X*** is a *n*-column vector containing all subjects’ environmental variables, ***M*** is a *n *×* p* design matrix with the *i*th row ${\left[1,{\tilde{Z}}_{i}^{T},G_{i},D_{i},G_{i}D_{i}\right]}^{T},\;\beta ={\left[\beta_{0},\beta_{z},\beta_{g},\beta_{d},\beta_{gd}\right]}^{T}$ is the unknown p×1 parameters. Then, ${\hat{\beta }}_{gd}$ is the last element of $\hat{\beta }={\left(M^{T}M\right)}^{-1}M^{T}X$, and ${\hat{\sigma }}^{2}=\frac{S^{2}}{n-p}$, where *S*^2^ is the sum of the squared error, *i.e.*, S2=||X−Mβ^||2. The variance of ${\hat{\beta }}_{gd}/{\hat{\sigma }}^{2}$ can be derived based on the delta method; that is, $\text{var}({\hat{\beta }}_{gd}/{\hat{\sigma }}^{2})=[\frac{1}{{\hat{\sigma }}^{2}},-\frac{{\hat{\beta }}_{gd}}{{\hat{\sigma }}^{4}}]\hat{\Sigma }{\left[\frac{1}{{\hat{\sigma }}^{2}},-\frac{{\hat{\beta }}_{gd}}{{\hat{\sigma }}^{4}}\right]}^{T}$, where Σ is the variance-covariance matrix of ${\left[{\hat{\beta }}_{gd},{\hat{\sigma }}^{2}\right]}^{T}$.

We can use the sandwich method to estimate variance-covariance matrix Σ, based on the following system of estimating equation:

$$\{\begin{array}{c} M^{T}(X-\mathrm{M\beta})=0,\\ \frac{{\left(X-\mathrm{M\beta}\right)}^{T}(X-\mathrm{M\beta})}{n-p}-\sigma^{2}=0. \end{array}$$

The point estimate and 95% confidence interval of ROR can be obtained by $\text{exp}({\hat{\beta }}_{gd}/{\hat{\sigma }}^{2})$ and $\text{exp}({\hat{\beta }}_{gd}/{\hat{\sigma }}^{2}\pm 1.96\times \sqrt{\hat{\text{var}}({\hat{\beta }}_{gd}/{\hat{\sigma }}^{2})})$ respectively.

### Appendix C: Validity of the reverse test under a case-control study design

Consider a source population for which we can use the reverse approach to specify the conditional distribution f(X|D,G,Z), and a case-control study is formed based on this source population. The sampling probability for selecting a subject into a case-control study does not depend on the exposure *X*, mathematically, we have that Pr(I=1|D,X,G,Z)=Pr(I=1|D,G,Z), where *I* is an indicator that equals 1 if this subject is selected into the case-control study and 0 otherwise. In the derived case-control study, the reverse test specifies the following distribution f(X|D,G,Z,I=1). By Bayes’ formula, we have that

$$\begin{array}{c} f(X|D,G,Z,I=1)=\frac{f(X,I=1|D,G,Z)}{P(I=1|D,G,Z)},\\ =\frac{P(I=1|X,D,G,Z)f(X|D,G,Z)}{P(I=1|D,G,Z)},\\ =\frac{P(I=1|D,G,Z)f(X|D,G,Z)}{P(I=1|D,G,Z)},\\ =f(X|D,G,Z). \end{array}$$

This indicates f(X|D,G,Z,I=1)=f(X|D,G,Z) and therefore the case-control sampling scheme does not affect the validity of the reverse test.
