# Supplementary material for: Testing gene–environment interactions in the presence of confounders and mismeasured environmental exposures
Source: G3 (Bethesda). 2021 Jul 16;11(10):jkab236. doi: 10.1093/g3journal/jkab236 (PMC8473983; doi:10.1093/g3journal/jkab236)
Supplement: jkab236_Supplementary_Data [file jkab236_supplementary_data.zip › jkab236-suppl_data/GENETICS-G3-2021-402584-s01.zip!/LaTeX Source Files/main.pdf]

Testing Gene-Environment Interactions in the Presence of Confounders and Mismeasured Environmental Exposures

Chao Cheng[†,‡], Donna Spiegelman[†,‡], Zuoheng Wang[†], and Molin Wang[*§,††]

[†]Department of Biostatistics, Yale School of Public Health, New Haven, Connecticut, 06510, United States of America

[‡]Center for Methods in Implementation and Prevention Science, Yale School of Public Health, New Haven, Connecticut, 06510, United States of America

[§]Departments of Epidemiology and Biostatistics, Harvard T.H. Chan School of Public Health, Boston, Massachusetts, 02115, United States of America

[††]Channing Division of Network Medicine, Department of Medicine, Brigham and Women's Hospital, Harvard Medical School, Boston, Massachusetts, 02115, United States of America

[*]Corresponding to: Departments of Biostatistics and Epidemiology, Harvard T.H. Chan School of Public Health, Boston, MA, 02115, USA. E-mail: stmow@channing.harvard.edu

Running title: Testing Gene-Environment Interactions

Key words: computational efficiency; confounders; gene-environment interaction; measurement error; relative efficiency; reverse test.

Corresponding author:
Molin Wang, PhD
Departments of Epidemiology and Biostatistics,
Harvard T.H. Chan School of Public Health,
Boston, MA 02115.
Phone: 617-432-1412
E-mail: stmow@channing.harvard.edu

Abstract

Interest in investigating gene-environment (GxE) interactions has rapidly increased over the last decade. Although GxE interactions have been extremely investigated in large studies, few such effects have been identified and replicated, highlighting the need to develop statistical GxE tests with greater statistical power. The reverse test has been proposed for testing the interaction effect between a continuous exposure and genetic variants in relation to a binary disease outcome, which leverages the idea of linear discriminant analysis, significantly increasing statistical power comparing to the standard logistic regression approach. However, this reverse approach did not take into consideration adjustment for confounders. Since GxE interaction studies are inherently non-experimental, adjusting for potential confounding effects is critical for valid evaluation of GxE interactions. In this paper, we extend the reverse test to allow for confounders. The proposed reverse test also allows for exposure measurement errors as typically occurs. Extensive simulation experiments demonstrated that the proposed method not only provides greater statistical power under most simulation scenarios but also provides substantive computational efficiency, which achieves a computation time that is more than sevenfold less than that of the standard logistic regression test. In an illustrative example, we applied the proposed approach to the Veterans Aging Cohort Study (VACS) to search for genetic susceptibility loci modifying the smoking–HIV status association.

Keywords: computational efficiency; confounders; gene-environment interaction; measurement error; relative efficiency; reverse test.

INTRODUCTION

It is commonly believed that genetic variants can explain a proportion of the risk for most complex human diseases (e.g., cancer, diabetes, asthma), where additional unexplained risk could be explained, in part, by gene-environment (GxE) interactions (Thomas 2010). Although many studies have attempted to identify genetic susceptibility loci interacting with environmental exposures in samples using logistic regression analysis, few such interactions have been identified and replicated (Aschard *et al.* 2012). Reasons for the failure to detect GxE interactions may include: 1) insufficient power for testing interaction effects compared to testing main effects (Smith and Day, 1984); 2) measurement errors in the environmental exposure sacrifice the power of the statistical test; 3) relatively small interaction effect sizes compared to the main effect sizes of genetic variants and environmental exposures, and 4) a large number of interactions to be tested as compared to a relatively small sample size in epidemiological studies. In addition, since GxE research is inherently non-experimental, it is important to control for potential confounders (Keller, 2014). For example, it is important to adjust for population stratification so that the interaction effects detected between genetic variants and exposures are not driven by ethnicity (Wang *et al.* 2006; Wang and Lee, 2008; Witte *et al.* 1999; Kraft and Hunter, 2005). In an attempt to minimize the number of covariates in the logistic regression model due to the concern about low power, investigators usually enter the confounders as covariates into the regression model, and ignore the potential confounder-environment or confounder-gene interaction effects. Several authors (e.g., Keller, 2014; VanderWeele *et al.* 2013) suggested to take into consideration the possible confounder-environment and confounder-gene interactions in order to obtain a more robust analysis result.

In this article, we focus on statistical methods for testing the interaction effect between

a genetic variant and a continuous environmental exposure on a binary disease outcome. The genetic variant can be either binary or ordinal. When the environmental exposure is a continuous variable, Aschard *et al.* (2018) proposed a test that “reverses" the role of the disease status and the continuous exposure in the logistic regression model, i.e., treating the disease status as an independent variable and the environmental exposure as the dependent variable. This regression model is now a linear regression including an interaction term between the genetic variant and disease status, encoding the GxE interaction effect. When the error term in this linear regression model follows a constant-variance normal distribution, both the reverse test and the standard logistic regression test evaluate the same null hypothesis (Aschard *et al.* 2018). The reverse test has several advantages over the logistic regression approach. First, the reverse test approach generally uses less computation time, since the reverse test statistic has a closed form, in contrast to the iterative optimization procedure used in logistic regression. Simulation studies and some theoretical analysis that follows indicate that the reverse test tends to achieve a several- to ten-fold reduction in computation time compared to the logistic regression test, as the sample size becomes large. Second, in the reverse test approach, measurement error in the environmental exposure does not cause bias in the point estimates of the regression coefficients, because the measurement error is adsorbed in the error term. Third, the reverse test approach exhibits greater statistical power than the logistic regression approach, especially when the main effect of the exposure and the GxE interaction effect are large.

The reverse test in Aschard *et al.* (2018) did not consider confounders except for a brief discussion on this issue, whereas, as pointed out above, in GxE interaction studies it is usually necessary to control for a set of potential confounders. In this paper, we extend the reverse test

to adjust for confounders in the evaluation of the GxE interaction. Specifically, we introduce confounder terms into the linear regression model, which include not only the main effect of confounders but also the interaction effects between the confounders and gene/disease. In the scenarios where the confounder-environmental exposure relationship may be nonlinear, spline terms for the confounders can be incorporated in model. We perform simulation studies to evaluate the type I error rate and power of this test and compare it to the standard logistic regression approach. In an illustrative example, we apply the reverse test to the Veterans Aging Cohort Study (VACS) to investigate genetic variants modifying the association of smoking and HIV status.

MATERIALS AND METHODS

The logistic regression approach

In genome-wide association studies (GWAS), the logistic regression model has been commonly used to estimate the GxE interaction effects and test for their presence. This model has the following form

$$\text{logit}\big(Pr(D=1|G,X,\boldsymbol{Z})\big)=\alpha_0+m(\boldsymbol{Z})+\alpha_x X+\alpha_g G+\alpha_{gx}GX, \tag{1}$$

where $\text{logit}(\gamma)=\log\left(\frac{\gamma}{1-\gamma}\right)$ is the logit function, and X, G, D and $\boldsymbol{Z}=[Z_1,\ldots,Z_p]^T$ denote a continuous exposure, a genetic variant, a binary disease status and possible confounders, respectively. We first consider a binary genetic variant, $G=\{0,1\}$, to denote the risk allele non-carriers and carriers. The ordinal scenario for G will be considered at the end of this subsection. The confounders, Z_j $(j=1,\ldots,p)$, can be continuous or binary. Here, e^{α_x},

denoted as $\text{OR}(D|X, G = 0)$, is the odds ratio (OR) for the disease for a one unit increase in X at the reference genetic level $G = 0$, e^{α_g}, denoted as $\text{OR}(D|G, X = 0)$, is the OR for $G = 1$ versus $G = 0$ at the reference exposure level $X = 0$. The parameter of interest is $e^{\alpha_{gx}} = \frac{\text{OR}(D|X,G=1)}{\text{OR}(D|X,G=0)}$, which is the ratio of the ORs (ROR) with respect to X for $G = 1$ versus $G = 0$, representing the effect of the GxE interaction. The null hypothesis, ROR $= 1$, or equivalently $\alpha_{gx} = 0$, indicates no interaction between the genetic variant and environmental exposure.

Without loss of generality, in the logistic regression model (1), we assume that the confounders term, $m(\boldsymbol{Z})$, can be written as $\sum_{j=1}^{p} m_j(Z_j)$. For binary Z_j, $m_j(Z_j) = \beta_{z_j} Z_j$. For continuous variable Z_j, we may use the restricted cubic spline (Durrleman and Simon, 1989) or other spline functions to model Z_j. If using restricted cubic splines, we have $m_j(Z_j) = \sum_{k=1}^{M-1} \alpha_{z_j,k} B_{j,k}(Z_j)$, where $\{B_{j,k}(Z_j)\}_{k=1}^{M-1}$ is the spline basis based on M knots (Durrleman and Simon, 1989). If $M = 2$, $m_j(Z_j)$ includes only a linear term, $\alpha_{z_j,1} Z_j$. The knots are often placed at evenly-spaced percentiles over the distribution of Z_j. The confounder term, $m(\boldsymbol{Z})$, can be rewritten as $\boldsymbol{\alpha}_z^T \tilde{\boldsymbol{Z}}$, where the vector $\tilde{\boldsymbol{Z}}$ denotes all the terms generated by $\boldsymbol{Z}$, including all the possible spline basis, and $\boldsymbol{\alpha}_z$ are the corresponding coefficients. Throughout this paper, all vectors are column vectors. Generally, the maximum likelihood estimator of $\boldsymbol{\alpha} = [\alpha_0, \boldsymbol{\alpha}_{\boldsymbol{z}}^T, \alpha_x, \alpha_g, \alpha_{gx}]^T$ can be obtained through an iteratively procedure. A 1-df wald test with respect to the null hypothesis, $H_0 : \alpha_{gx} = 0$, can be used to test the GxE interaction. The wald test statistic is $\tau_\alpha^2 = \frac{\hat{\alpha}_{gx}^2}{\widehat{\text{Var}}(\hat{\alpha}_{gx})}$, which follows a 1-df χ^2-distribution under the null.

In the above, Z-G or Z-X interaction effects have not been included in model (1). Vanderweele *et al.* (2013) and Keller (2014) pointed out that neglecting Z-G interaction terms could bias estimates of the GxE interaction and inflate the type I error of the null hypothesis. A

similar argument applies to the Z-X interaction terms. Therefore, the Z-G and Z-X interaction terms must also be included in order to obtain valid GxE interaction estimates and tests. In model (1), it is straightforward to control for all the main effects and interaction effects of $\boldsymbol{Z}$ by replacing $\boldsymbol{Z}$ in the term $m(\boldsymbol{Z})$ with $\boldsymbol{Z}^* = [\boldsymbol{Z}^T, (\boldsymbol{Z}X)^T, (\boldsymbol{Z}G)^T]^T$. Inclusion of these terms will not change the estimation and test procedure for the GxE interaction effect.

The genetic variant, G, may also be treated as an ordinal variable with values 0, 1 and 2, corresponding to wild type, heterozygous genotype, and homozygous genotype. Under this scenario, e^{α_g} now represents the OR for one unit increase in G at the exposure reference level $X = 0$, and $e^{\alpha_{gx}}$, the ROR, represents the ratio of ORs in X for one unit increase of G. The parameter estimation and testing for $H_0 : \alpha_{gx} = 0$, or equivalently $H_0 : \text{ROR} = 1$, are similar to those in the binary genetic variant scenario.

The reverse test approach

Aschard *et al.* (2018) proposed a reverse test that exchanges the roles of the disease outcome D and continuous exposure X. Now, taking into account potential confounders, $\boldsymbol{Z}$, we assume X, conditional on D, G and $\boldsymbol{Z}$, follows a normal distribution with constant variance; i.e.,

$$X = \beta_0 + h(\boldsymbol{Z}) + \beta_g G + \beta_d D + \beta_{gd} GD + \epsilon, \tag{2}$$

where $\epsilon \sim N(0, \sigma^2)$. Similar to $m(\boldsymbol{Z})$ in the logistic regression model, $h(\boldsymbol{Z})$ can be written as $\sum_{j=1}^{p} h_j(Z_j)$ with each of $h_j(Z_j)$ a linear form $\beta_{z_j} Z_j$ or a spline function. Estimation of the unknown parameters in (2), denoted by $\boldsymbol{\beta}$, can use ordinary least squares (OLS). Unlike with the logistic regression model (1), the parameter estimators for this linear regression model have a closed form. Thus, this linear regression approach is computationally more efficient

than the logistic regression approach.

Now, we calculate the ROR based on the linear regression model (2). For notational simplicity, we assume the genetic variant is binary. The approach for ordinal genetic variants will be similar. In Appendix A, we show that $\text{OR}(D|X, G = g) = e^{(\beta_d + \beta_{gd} g)/\sigma^2}$ for $g = 0, 1$. Therefore, $\text{ROR} = e^{\beta_{gd}/\sigma^2}$, and testing $H_0 : \text{ROR} = 1$ is equivalent to testing $H_0 : \beta_{gd} = 0$ in linear model (2). Also, using the linear regression model, the ROR can be estimated by $\widehat{\text{ROR}} = \exp(\hat{\beta}_{gd}/\hat{\sigma}^2)$, where $\hat{\beta}_{gd}$ and $\hat{\sigma}^2$ are the OLS estimates of β_{gd} and the estimated variance of the error term, σ^2. The analytic formula for the standard error and estimated confidence interval of $\widehat{\text{ROR}}$ are shown in Appendix B. In the linear model, we can also control for possible interaction effects between confounders and gene/disease by replacing $\boldsymbol{Z}$ in the term $h(\boldsymbol{Z})$ with $\boldsymbol{Z}^{**} = [\boldsymbol{Z}^T, (\boldsymbol{Z}D)^T, (\boldsymbol{Z}G)^T]^T$. In this linear model including the additional interactions, e^{β_{gd}/σ^2} also represents the ROR (see Supplementary Material Appendix A for more detail); therefore, testing $\beta_{gd} = 0$ is also valid for testing $\text{ROR} = 1$. Similar to the logistic regression test, the reverse test can uses a 1-df wald test statistic $\tau_\beta^2 = \frac{\hat{\beta}_{gd}^2}{\widehat{\text{Var}}(\hat{\beta}_{gd})}$ to check for the null hypothesis $H_0 : \beta_{gd} = 0$. One of major advantages of the logistic regression approach is that it can provide valid statistical inference if the data is a case-control study that is retrospectively sampled from a source population with known disease status. In Appendix C, we prove that the reverse test is also a valid approach under such a case-control sampling design.

Consider the following linear model with $\boldsymbol{Z}$-G interaction terms,

$$\begin{cases} X = \beta_0 + \tilde{h}(\boldsymbol{Z}) + \tilde{\tilde{h}}(\boldsymbol{Z})G + \beta_g G + \beta_d D + \beta_{gd} GD + \epsilon, & \text{if } G \text{ is binary}, \\ X = \beta_0 + \tilde{h}(\boldsymbol{Z}) + \tilde{\tilde{h}}(\boldsymbol{Z})G + \tilde{\tilde{\tilde{h}}}(\boldsymbol{Z})G^2 + \beta_g G + \beta_d D + \beta_{gd} GD + \epsilon, & \text{if } G \text{ is ordinal}, \end{cases} \tag{3}$$

where $\tilde{h}(\boldsymbol{Z})$, $\tilde{\tilde{h}}(\boldsymbol{Z})$ and $\tilde{\tilde{\tilde{h}}}(\boldsymbol{Z})$ are functions of $\boldsymbol{Z}$ and $\epsilon \sim N(0, \sigma^2)$ as for (2). Note that

model (2) is a special case of (3). As shown in Supplementary Material Appendix B, the logistic model (1) and linear model (3) can hold simultaneously, and if both models hold, we can develop the following parametric relationships between the logistic regression and linear models: $\alpha_x = \frac{\beta_d}{\sigma^2}$ and $\alpha_{gx} = \frac{\beta_{gd}}{\sigma^2}$. Otherwise, however, the logistic regression model (1) and linear model (2) cannot hold simultaneously. In Supplementary Material Appendix B we show that models (1) and (2) can also hold simultaneously and the parametric relationships above still hold. This occurs under the following two conditions. First, the error term in the linear model (2) is normally distributed with a constant variance. Second, $\text{logit}(Pr(D=1|G,\boldsymbol{Z})) = b_0+b_1G+b_2G^2+b_z(\boldsymbol{Z})+b_{zg}(\boldsymbol{Z},G)$; where G can be binary or ordinal, and b_0, b_1, b_2, $b_z(\boldsymbol{Z})$, and $b_{zg}(\boldsymbol{Z},G)$ are functions of the unknown parameters in models (1) and (2), and the definitions of b's are shown in Supplementary Material formula (S2).

The linear regression model (2) mainly relies on two assumptions: i) the conditional normality assumption; i.e., the error term in the linear model follows a normal distribution and ii) the constant variance assumption; i.e., $\text{Var}(\epsilon) = \sigma^2$ is constant. A normality test, such as the Jarque-Bera test (Jarque and Bera 1987) or the Shapiro-Wilk test (Shapiro and Wilk 1965), can be used to check whether regression residuals, ϵ, satisfy the conditional normality assumption. If necessary, a transformation on X can be applied before performing the GxE analysis using the reverse test approach. Commonly used transformations include logarithm, square root, Box-Cox (Box and Cox 1964), and Yeo-Johnson transformations (Yeo and Johnson 2000).

The constant variance assumption can be evaluated by implementing Levene's test (Levene 1961) or the White test (White 1980) on the linear regression model's residuals. When the constant variance assumption does not hold, i.e., $\text{Var}(\epsilon)$ depends on $\boldsymbol{Z}$, D and G, the OLS

estimator of β_{gd} will be consistent but the variance estimator of $\hat{\beta}_{gd}$ is invalid, often inflating the type I error for $H_0 : \beta_{gd} = 0$. Under heteroskedasticity, the sandwich variance should be used for $\widehat{\text{Var}}(\hat{\beta}_{gd})$ in $\tau_{\beta}^2 = (\frac{\hat{\beta}_{gd}}{\sqrt{\widehat{\text{Var}}(\hat{\beta}_{gd})}})^2$. This τ_{β}^2 statistic also follows a 1-df χ^2-distribution under the null hypothesis. Sun *et al.* (2018) suggested that the sandwich estimator of variance may be biased downwards in finite samples and cause inflated type I error in hypothesis testing, and provided a bootstrap-based method, referred as BICS, to improve the performance of the τ^2 statistic. It should be noted that, in the presence of heteroskedasticity, testing $H_0 : \beta_{gd} = 0$ is no longer equivalent to testing ROR $= 1$. In the next section, we present alternative definitions of the GxE interaction and show that testing $H_0 : \beta_{gd} = 0$ is still a valid test for GxE interaction, even if the constant variance or the conditional normality assumption does not hold.

Alternative definitions of the GxE interaction

In the previous two sections, the odds ratio was used to represent the exposure-disease association; if the exposure-disease association differs in genetic subgroups, a GxE interaction was identified. In this section, we present several other parameters that also represent the exposure-disease association, leading to alternative definitions for GxE interactions.

The conditional mean difference of X between cases and controls (abbreviated as MD), i.e., $E(X|D = 1, G, \boldsymbol{Z}) - E(X|D = 0, G, \boldsymbol{Z})$, can be used to represent the exposure-disease association. The difference of MD between the subgroups of $G = 1$ and $G = 0$, referred as the *difference of mean difference* (DMD), can represent the GxE interaction effect, and DMD $= 0$ represents no GxE interaction (Aschard *et al.* 2018). In model (2), we can show the MDs at the genetic levels $G = 1$ and $G = 0$ are $\beta_d + \beta_{gd}$ and β_d, respectively, and therefore DMD $= \beta_{gd}$.

Noticing that the previous derivation of MD and DMD only requires that $E(X|D, G, \boldsymbol{Z})$ agrees with what is presented in (2), it does not place any restriction on the distribution of the error term in linear model (2). In other words, even if ϵ in (2) is non-normally distributed and heteroskedastic, $H_0 : \beta_{gd} = 0$ is valid for testing for no GxE interaction $H_0 : \text{DMD} = 0$.

Alternatively, $\text{Corr}(X, D|G, \boldsymbol{Z})$ also measures the exposure-disease association, and the *difference in correlation coefficient* (DCC), defined as $\text{Corr}(X, D|G = 1, \boldsymbol{Z}) - \text{Corr}(X, D|G = 0, \boldsymbol{Z})$, also represents GxE interaction. If DCC $= 0$, there is no exposure-disease correlation difference across genetic subgroups, and therefore no GxE interaction. In Supplementary Material Appendix C, we show that testing for $H_0 : \text{DCC} = 0$ is also valid through evaluating $H_0 : \beta_{gd} = 0$, under the assumptions of weak X-G and D-G associations, regardless of whether the normality assumption holds or not.

Measurement errors in the environmental exposure

Many environmental exposures are measured with error, including data from self-administered questionnaires and laboratory measurements. In this section, we consider scenario under the classical additive measurement error model:

$$X^* = X + \delta, \tag{4}$$

where X^* is the measurement of X, and δ, independent from X, G, $\boldsymbol{Z}$ and D, is the measurement error term following a mean zero normal distribution with variance σ_δ^2. We can use the regression calibration method (Rosner, Willett and Spiegelman, 1989; Rosner, Spiegelman and Willett, 1990) to obtain the corrected estimator for the interaction coefficient, $\hat{\alpha}_{gx}$, in the logistic regression model (1). Using the regression calibration approach, we fit the logistic

regression model with X replaced by X^*:

$$\text{logit}\big(Pr(D=1|G,X^*,\boldsymbol{Z})\big) = \alpha_0^* + m^*(\boldsymbol{Z}) + \alpha_x^* X^* + \alpha_g^* G + \alpha_{gx}^* G X^*. \tag{5}$$

It follows that $\hat{\alpha}_{gx} \approx \hat{\alpha}_{gx}^*/\hat{\rho}$, where $\hat{\rho} = \frac{\widehat{\text{Var}}(X)}{\widehat{\text{Var}}(X)+\hat{\sigma}_\delta^2}$ and ρ represents the magnitude of the measurement error, when either i) $Pr(D=1|X,X^*,G,\boldsymbol{Z}) = Pr(D=1|X,G,\boldsymbol{Z})$, the disease is rare, and $X|G,\boldsymbol{Z}$ is normal (Rosner, Willett and Spiegelman, 1989); or ii) $Pr(D=1|X,X^*,G,\boldsymbol{Z}) = Pr(D=1|X,G,\boldsymbol{Z})$ and $\text{Var}(X|X^*,G,\boldsymbol{Z})$ is small (Carroll *et al.*, 2006). Under either of the above conditions, testing $H_0 : \alpha_{gx}^* = 0$ in model (5) is also a valid test for no GxE interaction $H_0 : \text{ROR} = 1$, although the point estimator $\hat{\alpha}_{gx}^*$ is attenuated.

It is more straightforward to cope with the measurement errors in the linear regression model (2). Based on the assumption that δ is independent from D, G and $\boldsymbol{Z}$, we can show that $E(X^*|G,D,\boldsymbol{Z}) = E(X+\delta|G,D,\boldsymbol{Z}) = E(X|G,D,\boldsymbol{Z})$ and $\text{Var}(X^*|G,D,\boldsymbol{Z}) = \text{Var}(X+\delta|G,D,\boldsymbol{Z}) = \sigma^2+\sigma_\delta^2$. Noting that both δ and ϵ are normally distributed, we have

$$X^* = \beta_0^* + h^*(\boldsymbol{Z}) + \beta_g^* G + \beta_d^* D + \beta_{gd}^* G D + \epsilon^*, \tag{6}$$

where $\epsilon^* \sim N(0, \sigma^2+\sigma_\delta^2)$. All of the coefficients in the conditional mean (6) equal those in the original linear model (2), including $\beta_{gd}^* = \beta_{gd}$. Now, the parametric relationship $\beta_{gd}^* = \beta_{gd}$ holds exactly, in contrast to the approximation for logistic regression using the regression calibration approach. Since $\text{ROR} = e^{\beta_{gd}/\sigma^2}$, we have $\text{ROR} = e^{\beta_{gd}^*/\sigma^2}$, and testing $H_0 : \beta_{gd}^* = 0$ in linear regression model (6) is valid for testing $H_0 : \text{ROR} = 1$. The $\widehat{\text{ROR}} = e^{\hat{\beta}_{gd}^*/\hat{\sigma}^2}$, however, will be attenuated, because $\hat{\sigma}^2$ tends to be overestimated as the variance of the error term, $\sigma^2+\sigma_\delta^2$, is larger than σ^2. If the measurement error term depends on G, D or $\boldsymbol{Z}$, i.e.,

$\text{Var}(\delta) = V(G, D, \boldsymbol{Z})\sigma_\delta^2$, where $V(G, D, \boldsymbol{Z})$ is an unknown positive function, the linear model (6) with $\beta_{gd}^* = \beta_{gd}$ still holds. However, $\text{Var}(\epsilon^*) = \sigma^2 + V(G, D, \boldsymbol{Z})\sigma_\delta^2$ is no longer a constant. As discussed in the section above, in this heteroskedasticity scenario, we can use the sandwich variance in the test for $H_0 : \beta_{gd} = 0$.

Simulation studies

To assess the validity of the reverse test approach and its power compared to the standard logistic regression model approach, we conducted simulation studies under a range of scenarios. We describe first the models considered in the simulation study, and then the data generation procedures.

Specifically, we consider the following logistic and linear regression model:

$$\begin{cases} \text{logit}\Big(Pr(D = 1|G, X, \boldsymbol{Z})\Big) = \alpha_0 + \alpha_1 Z_1 + \alpha_2 Z_1^2 + \alpha_3 Z_2 + \alpha_g G + \alpha_x X + \alpha_{gx} GX, \\ X = \beta_0 + \beta_1 Z_1 + \beta_2 Z_1^2 + \beta_3 Z_2 + \beta_g G + \beta_d D + \beta_{gd} GD + \epsilon, \end{cases} \tag{7}$$

where $\epsilon \sim N(0, 1)$, and Z_1 and Z_2 are potential confounders. A brief summary of the data generation process is shown as below and the detailed information can be found in Supplementary Material Appendix D. Specifically, we first generate Z_1 and Z_2, followed by the genetic variant G. Next the disease D is generated conditional on Z_1, Z_2, and G. Finally, the exposure X is generated based on the linear regression model in (7). Note that the logistic model in (7) also holds by this generation procedure, since the data generating process for $D|G, Z_1, Z_2$ are carefully manipulated such that the distribution $D|X, G, Z_1, Z_2$ coincides with the linear model in (7). We now specify the parameter values in (7). Noting that $\alpha_0 = \log(\frac{Pr(D=1|G=X=Z_1=Z_2=0)}{1-Pr(D=1|G=X=Z_1=Z_2=0)})$, we choose the values of α_0 such that the baseline disease

prevalence ranged from 5% to 50%. In the logistic regression model, we set e^{α_1}, e^{α_2} and e^{α_3} as 1.2, and specified α_x, α_g and α_{gx} over realistic values such that $\text{OR}(D|X, G = 0) \leq 1.5$, $\text{OR}(D|G, X = 0) \leq 1.5$ and $\text{ROR} \leq 1.5$, each corresponding to a one unit increase in X and G. In the linear regression model, we fixed $\beta_0 = 0$, $\beta_1 = 0.03$ and $\beta_2 = 0.03$, and defined β_3 and β_g for a series of correlations between the outcome X and independent variables G, D and $\boldsymbol{Z}$, with $\beta_3 = \frac{\text{Corr}(X,Z_2|Z_1=G=D=0)\sqrt{\text{Var}(X|Z_1=G=D=0)}}{\sqrt{\text{Var}(Z_2|Z_1=G=D=0)}}$, and $\beta_g = \frac{\text{Corr}(X,G|Z_1=Z_2=D=0)\sqrt{\text{Var}(X|Z_1=Z_2=D=0)}}{\sqrt{\text{Var}(G|Z_1=Z_2=D=0)}}$, where $\text{Corr}(X, Z_2|Z_1 = G = D = 0)$ and $\text{Corr}(X, G|Z_1 = Z_2 = D = 0)$ were set at a range from 0.01 to 0.2, the conditional variances of X were specified as 1, and the conditional variances of G and Z_2 were set to their corresponding unconditional variances. We specified $\beta_d = \alpha_x$ and $\beta_{gd} = \alpha_{gx}$, noting that $\beta_d = \alpha_x\sigma^2$, $\beta_{gd} = \alpha_{gx}\sigma^2$, and $\sigma^2 = 1$.

We first simulated a cohort study with 100,000 subjects using the parameters and data generation procedure as above, and then randomly selected 1,000 cases and 1,000 controls from the cohort to create a 1:1 matched case-control study. Then, we fit the logistic model and linear model to obtain $\hat{\alpha}_{gx}$ and $\hat{\beta}_{gd}$, where a cubic B-spline approach with 3 interior knots was used for Z_1 in each model. Next, we tested $H_0 : \alpha_{gx} = 0$ and $H_0 : \beta_{gx} = 0$ based on a 1-df wald test. We repeated the above procedure 500,000 times to evaluate the validity and statistical power of the two tests. Under measurement error scenarios, we generated X^* based on (4), where the measurement error was set to explain 25%–75% of the variance of X^*; that is, $\rho = \frac{\text{Var}(X)}{\text{Var}(X)+\sigma_\delta^2}$ from 75% to 25%. Here, $\hat{\alpha}^*_{gx}$ and $\hat{\beta}^*_{gd}$ were estimated from X^* instead of X in two models.

In addition, we compared performance of the two tests when models (1) and (2) do not hold simultaneously. Specifically, we considered two scenarios, corresponding to the two conditions that are required for both models hold simultaneously. In Scenario I, we used a simple logistic

regression model with probability $\text{logit}(P(D=1|G,\boldsymbol{Z})) = \alpha_0 + \alpha_1 Z_1 + \alpha_2 Z_1^2 + \alpha_3 Z_2^2 + \alpha_g G$ to generate the disease outcome, instead of the more complex expression given above to generate D in order to ensure both models hold simultaneously. In Scenario II, we considered scenarios where error term in the linear model does not follow normality and set the error term to follow a rectified Gaussian distribution (Socci *et al.* 1998), a right-skewed distribution that resets the negative elements of a normal distribution to 0. In each of these scenarios, we considered two data generation procedures, referred as the *logistic data generation procedure* and *linear data generation procedure*, corresponding to the following two cases: (i) a correctly specified logistic model but a misspecified linear model and (ii) a correctly specified linear model but a misspecified logistic model. More details for the the simulation setting-ups in Scenarios I and II are deferred to Supplementary Material Appendix E and F, respectively.

Illustrative example: the Veterans Aging Cohort Study

We illustrated the utility of the proposed reverse test by investigating the genome-wide interaction of gene-smoking on HIV status from the Veterans Aging Cohort Study (VACS). VACS is a multi-center, longitudinal observational study of HIV-infected and -uninfected veterans, whose primary objective is to understand the risk of substance abuse in subjects with HIV infection (Justice *et al.* 2006; Wu *et al.* 2019). As the GxE interaction effects may vary among different ethnic groups, we focused on the subgroup of African Americans in this example. The environmental exposure, smoking, was measured by cigarettes per day (≥ 0) in patient surveys collected at six clinic visits. Previous literature shows that cigarette smoking is a potential risk factor for HIV acquisition as it may be associated with high-risk sexual behavior (Marshall et al., 2009; Burns et al., 1991). For each subject, we defined smoking (cigarettes per

day) as the average of the smoking data over the six clinic visits. The distribution of smoking is highly right-skewed with the coefficient of skewness 3.54 (Supplementary Material Figure S2 (a)). To alleviate the skewness, a Yeo-Johnson transformation (Yeo and Johnson 2000) was applied. Specifically, the transformed smoking value is $T(x) = \frac{(x+1)^{\gamma}-1}{\gamma}$, where x is the original smoking values and γ is a tuning parameter which equals $\gamma = -0.074$ here. Because γ is close to 0, we can make the following approximation by L'Hopital's rule: $T(x) \approx (x+1)^{\gamma} \times \log(x+1) \approx \log(x+1)$. Therefore, approximately, we can interpret the transformed smoking values as the logarithm of one plus the number of cigarettes per day. The density plots for the transformed exposure are shown in Supplementary Material Figure S2 (b), which presents that the transformed smoking variable is barely skewed, with the coefficient of skewness 0.03. In the reverse test, we used the transformed smoking varible as the response. We also used the transformed smoking variable in the logistic regression model.

All samples were genotyped on the Illumina OmniExpress BeadChip, and then imputed using IMPUTE2 (Howie *et al.* 2009) with the 1000 Genomes Phase 3 data as a reference panel, which resulted in a total of 17,092,657 SNPs. In this application study, we excluded subjects whose environmental exposure or HIV status were unavailable, and among that, the proportion of successfully imputed SNPs $< 95\%$. Thus, after data cleaning, 1,484 subjects were retained in the analysis, with 1,403 males and 81 females, of whom 965 were HIV positive and 519 were HIV negative. The characteristics of the 1,484 subjects stratified by the HIV status are provided in Supplementary Material Table S1, where the HIV negative group has lower proportions of males and smokers, whereas other characteristics are balanced between the HIV positive and negative groups. SNPs with minor allele frequency (MAF) $> 1\%$ and call rate $> 95\%$ were included in the analysis, which resulted in 10,079,672 SNPs for analysis

of their interaction effects with smoking on the HIV infection. As for the confounders in the logistic and linear model, we considered age at baseline (continuous), and gender, as well as top 10 Principal Components of genotypes to control for population structure. We also consider alcohol intake (binary, drinkers versus non-drinkers) as a potential confounder in the smoking-HIV association because evidence shows that alcohol consumption may be associated with cigarette smoking and is also a potential risk factor for the incidence of HIV (Shuper *et al.*, 2010). In the analysis, strong non-linear main effects of age at baseline were observed. Thus, we used the cubic spline with 3 interior knots to adjust for the main effect of age in both models. We also included age-by-smoking and age-by-gene interactions in the logistic model to control for possible interaction effects between age at baseline and smoking/gene on disease outcome. Similarly, age-by-gene and age-by-HIV interaction terms were included in our reverse test.

RESULTS

Type I error

We evaluated the validity of the reverse approach and logistic regression approach for testing GxE interaction by calculating the nominal type I error rates at 5% and 0.01% significant levels. Table 1 presents the empirical type I error rates of these tests over a range of scenarios, with and without measurement error in X in the circumstance when both the logistic regression and linear models hold. The null models were simulated by setting $\alpha_{gx} = \log(\text{ROR}) = 0$ in the data simulation procedure. As shown in Table 1, type I error rates were close to the nominal P-value thresholds over the full range of design parameters we studied for both tests,

regardless of whether there was measurement error.

We evaluated the performance of both tests in Scenarios I and II where the logistic regression model (1) and linear model (2) do not hold simultaneously. Table 2 shows the type I error rates at a 0.01% significant level for both tests in Scenario I where $P(D|G, \boldsymbol{Z})$ follows a simple logistic regression model. The reverse test approach had well controlled type I error rates under both the logistic and the linear data generation procedures, whereas the logistic regression test exhibited very slight type I error deflation under the linear data generation procedure. At the 5% significant level, both approaches always presented satisfactory type I error rates when either the linear or the logistic regression model did not hold simultaneously (Supplementary Material Table S2).

Supplementary Material Tables S3 and S4 provided empirical type I error rates in Scenario II where the error term in the linear model follows a rectified gaussian distribution. Under the linear data generation procedure, the reverse test exhibited no inflation or deflation at any of the nominal threshold considered; in contrast, the logistic regression test had inflated type I error under most settings considered, especially when the odds ratio of X was large without measurement error. Under the logistic data generation procedure, the logistic regression test had well controlled empirical type I error rates; for the reverse test, while the type I error rates of the reverse test were almost always below the nominal levels with occasionally a slight deflation.

Power comparison

We compared the statistical power of the logistic regression test and reverse test by calculating the ratio $\frac{\bar{\chi}^2_{\text{rev}}}{\bar{\chi}^2_{\text{log}}}$, simply referring as χ^2 ratio henceforth, where $\bar{\chi}^2_{\text{rev}}$ and $\bar{\chi}^2_{\text{log}}$ are the average

χ^2 test statistics of the reverse test and logistic regression test over 500,000 repetitions for each simulation study scenario. A χ^2 ratio greater than 1 indicates that the reverse test obtained statistical power than the logistic regression test.

Figure 1 provides the χ^2 ratio of the reverse test against logistic regression test in the absence of measurement error in X. We considered the exposure main effects, i.e., $\text{OR}(D|X, G = 0)$, from 1.1 to 1.5, and GxE effects, i.e., ROR, from 1.1 to 1.5, while considering weak and strong exposure-confounder correlation ($\text{Corr}(X, Z_2|Z_1 = G = D = 0) = 0.01$, 0.2), weak and strong genetic variant main effects ($\text{OR}(D|G, X = 0) = 1.1$, 1.5), rare and common disease prevalence ($Pr(D = 1|G = X = Z_1 = Z_2 = 0)$=0.05, 0.2), and weak and strong gene-exposure correlation ($\text{Corr}(X, G|Z_1 = Z_2 = D = 0)$=0.01, 0.2). As shown in Figure 1, all the χ^2 ratios were greater than 1, indicating the reverse test provided higher statistical power than the logistic regression test. The power advantage of the reverse test against logistic regression improved with the increase of the main effect of exposure; for example, the χ^2 ratios were generally below 1.25 for $\text{OR}(D|X, G = 0) = 1.1$ and $\text{ROR} = 1.5$ and were greater than 1.45 for $\text{OR}(D|X, G = 0) = 1.5$ and $\text{ROR} = 1.5$. As the ROR increased, the reverse test also tended to have higher statistical power than the logistic regression test. However, we observed a slight decrease in the relative efficiency as the association between X and Z_2 and the exposure-gene association increased. The disease prevalence and disease-gene association had minimal influence on the relative efficiency of the two tests.

More detailed results for the power comparison between the reverse and logistic regression test are provided in Table 3, in which we investigate the impact of exposure measurement error on the relative efficiency. Typically, the magnitude of the measurement error resulted in a decrease in the relative efficiency of the reverse test versus the logistic regression test,

but the reverse test still provided a power advantage even when $\rho = 0.25$. For example, when $\text{OR}(D|X, G = 0) = 1.5$, the χ^2 ratio decreased from over 1.4 in the absence of measurement error ($\rho = 1$) to below 1.15 for $\rho = 0.25$. Supplementary Material Figure S5 presents the rejection rates (i.e., power) under the alternative hypothesis that ROR=1.5 across 50,000 replications for the logistic regression approach and the reverse test at significance levels of 0.01% and 5% when the logistic and linear regression models hold simultaneously. As expected, the rejection rate raises when measurement error decreases or significance level increases. The reverse test exhibits higher rejection rates under nearly all settings in the absence of measurement error and also provides power advantage under most simulation settings with a large measurement error ($\rho = 0.25$).

We also investigated relative efficiency when the linear and logistic regression models did not hold simultaneously. The results for Scenario I are shown in Supplementary Material Table S6. As expected, the reverse test exhibited a significant power advantage under the linear model data generation procedure. Under the logistic data generation procedure, the reverse test still provided power advantage among 62.5% of the simulation scenarios under a weak D-X association ($\text{OR}(D|X, G = 0) = 1.1$) and 50%, when $\text{OR}(D|X, G = 0) = 1.5$. Among the simulation scenarios where χ^2 ration < 1, only 5 out of 84 (6.0%) had χ^2 ratio below 0.9. Supplementary Material Table S7 presents the relative efficiency for Scenario II where the error term in the linear model followed a rectified gaussian distribution. Under the linear data generation procedure, the reverse test generally outperformed the logistic regression test, the χ^2 ratio was less than 1 in only 12 out of 192 (6.3%) simulation scenarios. Under the logistic data generation procedure, the reverse test still provided satisfactory results; the χ^2 ratio was less than 0.9 in 49 out of 192 (25.6%) simulation scenarios. In addition, we also

observed that the reverse test provided greater statistical power against the logistic regression test for small main effects of X and weak correlations between exposure and genetic variant.

Computational time

The reverse test is more computationally efficient than the logistic regression test, since it is based on a closed form test statistics, in contrast to the iterative procedure used by the logistic regression test. Table 4 displays the computation time of the two tests using R software on a single desktop computer (3.7 GHz CPU and 16 GB RAM) for GxE analysis of 1,000,000 SNPs with the sample sizes from 1,000 to 200,000. We can see that the reverse test outperformed the logistic regression test in all scenarios. Furthermore, as the sample size increased, the ratio of the computation time of the logistic regression test comparing to the reverse test increased. With a sample size of 200,000, an approximately 7.5-fold reduction in computation time was observed for the reverse test.

Illustrative example: gene-smoking interactions in VACS

Interaction effects between genetic variants and smoking in relation to HIV status was considered among 10,079,672 SNPs in 1,484 African Americans in VACS, using both the reverse test and logistic regression approach, adjusting for age, sex, alcohol intake, and the top ten Principal Components. Quantile-Quantile (QQ) plots and Manhattan plots for both tests are presented in Figure 2. The reverse test displayed no evidence of P-value inflation in the QQ plot (Figure 2 Panel B), with a genomic inflation factor, λ, close to 1, while the logistic regression test showed some evidence of inflation in the QQ plot (Figure 2 Panel D), with $\lambda = 1.07$ We observed a strong correlation between the P-values of the logistic and reverse

tests, with the correlation coefficient 0.82.

Table 5 presents the results for SNPs where at least one of the reverse and logistic regression tests provided a P-value below 1×10^{-6}. We can see the top ranked SNPs largely overlapped between the two approaches. No SNPs showed genome-wide significance (P-value $< 5 \times 10^{-8}$) for the interaction under the logistic regression approach. However, the reverse test presents that two SNPs, rs10744166 and rs10773060, have significant interaction effects (P-value = 3.71×10^{-8} and 3.42×10^{-8}). Both SNPs are located at gene *ZNF664* in chromosome 12, which was previously identified to be associated with clubfoot (Zhang *et al.*, 2014). The minor allele frequencies (MAFs) of the two SNPs were 45.6% and 45.4% in the study sample, and were slightly higher as compared to the 1000 Genomes (with MAF 37.5% and 37.4% respectively). In order to illustrate interactions across genotypes, averages of the smoking values (cigarettes per day) between the HIV-infected and -uninfected veterans stratified by different genotypes of rs10744166 are visualized in Figure 3 Panel A. As can be seen from the figure, an increase in the number of minor alleles of rs10744166 indicates a larger mean difference between the smoking values in the HIV-infected and -uninfected individuals, such that the mean difference for the genotype with two minor alleles is at nearly 3.5 times the mean difference for the genotype with no minor alleles. In other words, the minor allele in rs10744166 is associated with a stronger smoking-HIV infection relationship. A similar pattern between genotypes and mean differences were also found in rs10773060, as visualized in Figure 3 Panel B. A linkage disequilibrium analysis shows evidence that rs10744166 and rs10773060, along with the other four SNPs located at ZNF664 shown in Table 5, are in linkage disequilibria spanning a 1.5kb length (Figure 3 Panel C), where the squared allele frequency correlations R^2 for each pair of SNPs are ranged between 0.95 and 0.99. It is of note that there was limited information on

the detected SNPs given the small sample size of this illustrative example. Replication in an independent dataset is needed to confirm those associations.

We further performed separate analysis for gene-smoking interactions in the subgroup of smokers (n=905). The QQ and Manhattan plots of both tests are visualized in Supplementary Material Figure S3. We observed no SNPs exhibiting genome-wide significance based on either logistic regression test or reverse test. This is not surprising as the sample size is relatively small. Although rs10744166 and rs10773060 show genome-wide significance previously, both SNPs do not appear to be significant in the smokers subgroup by either the reverse test (P-value=0.08 and 0.09) and the logistic regression test (P-value=0.02 and 0.02).

DISCUSSION

In this paper, we propose a reverse test for interaction between an environmental exposure and a genetic variant on a binary disease status, adjusting for confounding. Comparing with the standard logistic regression test for interaction with a continuous or binary environmental exposure, the reverse test only applies to a continuous environmental exposure. This reverse test leverages the spirit of linear discriminant analysis by reversing the roles of the environmental exposure and disease status, and obtains a closed form of the GxE test statistic. Our analysis shows that when the error term in the linear model follows a normal distribution with constant variance, both the reverse and standard logistic regression approaches are valid for testing $H_0 : ROR = 1$. Compared to the logistic regression approach, the reverse test has a larger statistical power. As a trade-off, the reverse test approach additionally assumes that the exposure is continuous. The logistic regression approach has a wider range of applications, in which the exposure can be continuous, binary, count, and categorical variables.

The reverse approach can be extended to test for interactions between a genetic marker set and a continuous environmental exposure with a binary disease outcome. The standard approach is based upon the logistic regression model logit$\big[Pr(D=1|X,\boldsymbol{G},\boldsymbol{Z})\big]=\alpha_0+\boldsymbol{\alpha}_z^T\boldsymbol{Z}+\boldsymbol{\alpha}_g^T\boldsymbol{G}+\alpha_x X+\boldsymbol{\alpha}_{gx}^T\boldsymbol{S}$, where $\boldsymbol{G}=[G_1,G_2,\ldots,G_p]^T$ denotes a genetic marker set containing p genetic variants and $\boldsymbol{S}=[G_1X,G_2X,\ldots,G_pX]^T$ denotes the interaction terms between the genetic marker set and exposure. The null hypothesis for the interaction is $H_0:\boldsymbol{\alpha}_{gx}=0$. Following reverse approach, we assume a linear model for X, $X=\beta_0+\boldsymbol{\beta}_z^T\boldsymbol{Z}+\boldsymbol{\beta}_g^T\boldsymbol{G}+\beta_d D+\boldsymbol{\beta}_{gd}^T\boldsymbol{U}+\epsilon$, where $\epsilon\sim N(0,\sigma^2)$ and $\boldsymbol{U}=[G_1D,G_2D,\ldots,G_pD]^T$ is the interaction terms between the genetic set and disease status. We can also build a parametric relationship such that $\boldsymbol{\alpha}_{gx}=\frac{\boldsymbol{\beta}_{gd}}{\sigma^2}$. Then, testing for $H_0:\boldsymbol{\alpha}_{gx}=0$ in the logistic model can be evaluated through testing for $H_0:\boldsymbol{\beta}_{gd}=0$ in the linear model. Based on theory of linear discriminant analysis (Efron, 1975), it is expected that the reverse approach outperforms the reverse test with respective to the statistical power if linear model is correctly specified and the error term follows a homoskedastic normal distribution.

We conducted simulation studies across a wide range of scenarios to evaluate the performance of the proposed approach. Several observations followed from our simulation experiments. First, the reverse test produces correct type I error rates, both for standard or very small P-value threshold of 5% and 0.01%, whether or not there is exposure measurement error. Second, the reverse test generally exhibited greater statistical power than the standard logistic approach, and its relative statistical power improved when the magnitude of the GxE interaction effect or the main effect of exposure increased. Although measurement error in the exposure tended to diminish the power advantage of the reverse test, the reverse test still provided some power gain even under very severe measurement error of $\rho=0.25$. Third, the

reverse test is substantially more computationally efficient. It achieves a computation time that is more than sevenfold less than that of the logistic regression test, a great advantage in coping with large-scale genomic studies with millions of SNPs. Fourth, the proposed approach performed reasonably well when the error term is not normal. In summary, the reverse test provided a valid, powerful and computationally efficient alternative for investigating GxE interactions in large-scale genomic research.

Does the relative computational advantage of the reverse test change as sample size increases? In fact, the ratio of the computation time of the logistic regression test and reverse test is likely bounded by a constant when the sample size is sufficient large. Specifically, fitting a linear model with an ordinary least squares (OLS) algorithm has $O(np^2)$ computational complexity in general (Drineas *et al.* 2010; Iyer 2015), where n is the sample size and p is the number of unknown coefficients. The computational complexity of logistic regression model depends on the optimization algorithm used. One popular algorithm is iteratively reweighted least squares (IRLS), which is also the default algorithm for fitting generalized linear models in many statistical softwares (such as R). As an iterative algorithm, the IRLS solves a weighted least squares (WLS) subproblem at each iteration, and the running time of this WLS subproblem is comparable with the OLS algorithm if they share same number of sample size and unknown coefficients (see Supplementary Material Appendix G for more details). In other words, the additional computational burden of the logistic regression test compared to the reverse approach mainly depends on the number of the IRLS iterations, when both tests adjusted for same number of covariates. Although the number of IRLS iterations is affected by initial values, as discussed in Komarek (2014), it is typically around 5 or 10 in most of the scenarios and barely larger than 30. It follows that the computation time of the logistic

regression test based on IRLS tends to be several to ten times longer than the reverse test when the sample size is sufficient large. These sorts of gains in computational efficiency can become quite important when whole genome scanning, involving 6.4 billion SNPs, are to be undertaken.

In conclusion, given its power advantages and substantial benefits in computing time, the reverse test can be quite a useful tool in investigating GxE interactions, permitting whole genome scans over many exposures simultaneously, measured with or without measurement error.

DATA AVAILABILITY

The authors state that all data necessary for confirming the conclusions of the article are present within the article. A tutorial for implementing the proposed methods in R software is available at `https://github.com/chaochengstat/GxE2020`. The R codes for replicating the simulation studies and analysis of the VACS illustrative example are available at `https://github.com/chaochengstat/GxE2020/tree/main/Rcode_Genetics`. Supplementary Material for this manuscript is attached at the end of the article, which includes Supplementary Material Appendix A–G, Supplementary Material Figures S1–S3, and Supplementary Material Tables S1–S7.

ACKNOWLEDGEMENTS

The authors are thankful for Dr. Ke Xu and Weimiao Wu's help to access the Veterans Aging Cohort Study dataset.

FUNDING

The Veterans Aging Cohort Study is supported by NIH U10 AA013566, U24 AA020794, and U01 AA020790. This work was supported by NIH R01ES026246. Zuoheng Wang's research was supported in part by NIH K01AA023321 and NSF DMS1916246.

References

Aschard, H., Lutz, S., Maus, B., Duell, E. J., Fingerlin, T. E., Chatterjee, N., Kraft, P., and Van Steen, K. Challenges and opportunities in genome-wide environmental interaction (gwei) studies. Human Genetics, 131(10):1591–1613, 2012.

Aschard, H., Spiegelman, D., Laville, V., Kraft, P., and Wang, M. A test for gene–environment interaction in the presence of measurement error in the environmental variable. Genetic Epidemiology, 42(3):250–264, 2018.

Box, G. E. and Cox, D. R. An analysis of transformations. Journal of the Royal Statistical Society: Series B (Methodological), 26(2):211–243, 1964.

Burns, D. N., Krämer, A., Yellin, F., Fuchs, D., Wachter, H., DiGioia, R. A., Sanchez, W. C., Grossman, R. J., Gordin, F. M., Biggar, R. J., et al. Cigarette smoking: a modifier of human immunodeficiency virus type 1 infection? Journal of Acquired Immune Deficiency Syndromes, 4(1), 1991.

Carroll, R. J., Ruppert, D., Stefanski, L. A., and Crainiceanu, C. M. Measurement error in nonlinear models: a modern perspective. Chapman and Hall/CRC, 2006.

Drineas, P., Mahoney, M. W., Muthukrishnan, S., and Sarlós, T. Faster least squares approximation. Numerische Mathematik, 117(2):219–249, 2011.

Durrleman, S. and Simon, R. Flexible regression models with cubic splines. Statistics in medicine, 8(5):551–561, 1989.

Efron, B. The efficiency of logistic regression compared to normal discriminant analysis. Journal of the American Statistical Association, 70(352):892–898, 1975.

Howie, B. N., Donnelly, P., and Marchini, J. A flexible and accurate genotype imputation method for the next generation of genome-wide association studies. PLoS genetics, 5(6): e1000529, 2009.

Iyer, K. T. Computational complexity of data mining algorithms used in fraud detection. 2015.

Jarque, C. M. and Bera, A. K. A test for normality of observations and regression residuals. International Statistical Review/Revue Internationale de Statistique, pages 163–172, 1987.

Justice, A. C., Dombrowski, E., Conigliaro, J., Fultz, S. L., Gibson, D., Madenwald, T., Goulet, J., Simberkoff, M., Butt, A. A., Rimland, D., et al. Veterans aging cohort study (vacs): overview and description. Medical Care, 44(8 Suppl 2):S13, 2006.

Keller, M. C. Gene× environment interaction studies have not properly controlled for potential confounders: the problem and the (simple) solution. Biological Psychiatry, 75(1):18–24, 2014.

Komarek, P. Logistic regression for data mining and high-dimensional classification. Carnegie Mellon University, 2004.

Kraft, P. and Hunter, D. Integrating epidemiology and genetic association: the challenge of gene–environment interaction. Philosophical Transactions of the Royal Society B: Biological Sciences, 360(1460):1609–1616, 2005.

Levene, H. Robust tests for equality of variances. Contributions to Probability and Statistics. Essays in Honor of Harold Hotelling, pages 279–292, 1961.

Marshall, M. M., McCormack, M. C., and Kirk, G. D. Effect of cigarette smoking on hiv acquisition, progression, and mortality. AIDS Education and Prevention, 21(3_supplement): 28–39, 2009.

Rosner, B., Willett, W., and Spiegelman, D. Correction of logistic regression relative risk estimates and confidence intervals for systematic within-person measurement error. Statistics in Medicine, 8(9):1051–1069, 1989.

Rosner, B., Spiegelman, D., and Willett, W. C. Correction of logistic regression relative risk estimates and confidence intervals for measurement error: the case of multiple covariates measured with error. American Journal of Epidemiology, 132(4):734–745, 1990.

Shapiro, S. S. and Wilk, M. B. An analysis of variance test for normality (complete samples). Biometrika, 52(3/4):591–611, 1965.

Shuper, P. A., Neuman, M., Kanteres, F., Baliunas, D., Joharchi, N., and Rehm, J. Causal considerations on alcohol and hiv/aids—a systematic review. Alcohol & Alcoholism, 45(2): 159–166, 2010.

Smith, P. and Day, N. The design of case-control studies: the influence of confounding and interaction effects. International Journal of Epidemiology, 13(3):356–365, 1984.

Socci, N. D., Lee, D. D., and Sebastian Seung, H. The rectified gaussian distribution. Advances in Neural Information Processing Systems, pages 350–356, 1998.

Sun, R., Carroll, R. J., Christiani, D. C., and Lin, X. Testing for gene–environment interaction under exposure misspecification. Biometrics, 74(2):653–662, 2018.

Thomas, D. Gene–environment-wide association studies: emerging approaches. Nature Reviews Genetics, 11(4):259–272, 2010.

VanderWeele, T. J., Ko, Y.-A., and Mukherjee, B. Environmental confounding in gene-environment interaction studies. American Journal of Epidemiology, 178(1):144–152, 2013.

Wang, L.-Y. and Lee, W.-C. Population stratification bias in the case-only study for gene-environment interactions. American Journal of Epidemiology, 168(2):197–201, 2008.

Wang, Y., Localio, R., and Rebbeck, T. R. Evaluating bias due to population stratification in epidemiologic studies of gene-gene or gene-environment interactions. Cancer Epidemiology and Prevention Biomarkers, 15(1):124–132, 2006.

White, H. A heteroskedasticity-consistent covariance matrix estimator and a direct test for heteroskedasticity. Econometrica: Journal of the Econometric Society, pages 817–838, 1980.

Witte, J. S., Gauderman, W. J., and Thomas, D. C. Asymptotic bias and efficiency in case-control studies of candidate genes and gene-environment interactions: basic family designs. American Journal of Epidemiology, 149(8):693–705, 1999.

Wu, W., Wang, Z., Xu, K., Zhang, X., Amei, A., Gelernter, J., Zhao, H., Justice, A. C., and Wang, Z. Retrospective association analysis of longitudinal binary traits identifies important loci and pathways in cocaine use. Genetics, 213(4):1225–1236, 2019.

Yeo, I.-K. and Johnson, R. A. A new family of power transformations to improve normality or symmetry. Biometrika, 87(4):954–959, 2000.

Zhang, T.-X., Haller, G., Lin, P., Alvarado, D. M., Hecht, J. T., Blanton, S. H., Richards, B. S., Rice, J. P., Dobbs, M. B., and Gurnett, C. A. Genome-wide association study identifies new disease loci for isolated clubfoot. Journal of Medical Genetics, 51(5):334–339, 2014.

APPENDIX

Appendix A: Derivation of OR($D|X, G = g$) based on the linear regression model

First, by definition,

$$\text{OR}(D|X, G = g, \boldsymbol{Z} = \boldsymbol{z}) = \frac{Pr(D = 1|X = x + 1, G = g, \boldsymbol{Z} = \boldsymbol{z})/Pr(D = 0|X = x + 1, G = g, \boldsymbol{Z} = \boldsymbol{z})}{Pr(D = 1|X = x, G = g, \boldsymbol{Z} = \boldsymbol{z})/Pr(D = 0|X = x, G = g, \boldsymbol{Z} = \boldsymbol{z})},$$

Using the Bayes' theorem,

$$Pr(D|X, G = g, \boldsymbol{Z} = \boldsymbol{z}) = f(X|D, G = g, \boldsymbol{Z} = \boldsymbol{z})Pr(D|G = g, \boldsymbol{Z} = \boldsymbol{z})/f(X|G = g, \boldsymbol{Z} = \boldsymbol{z}),$$

where $f(.)$ denotes the probability density functions (p.d.f's). It follows that

$$\text{OR}(D|X, G = g, \boldsymbol{Z} = \boldsymbol{z}) = \frac{f(X = x + 1|D = 1, G = g, \boldsymbol{Z} = \boldsymbol{z})/f(X = x|D = 1, G = g, \boldsymbol{Z} = \boldsymbol{z})}{f(X = x + 1|D = 0, G = g, \boldsymbol{Z} = \boldsymbol{z})/f(X = x|D = 0, G = g, \boldsymbol{Z} = \boldsymbol{z})}. \tag{8}$$

Since $X|D,G,\boldsymbol{Z}$ follows a normal distribution with mean $E(X|D,G,\boldsymbol{Z})$ and variance σ^2, we have

$$\begin{aligned}
\mathrm{OR}(D|X,G=g,\boldsymbol{Z}=z) &= \frac{\frac{1}{\sqrt{2\pi}\sigma}e^{-\frac{\left(x+1-(\beta_0+h(\boldsymbol{z})+\beta_g g+\beta_d+\beta_{gd}g)\right)^2}{2\sigma^2}} \Big/ \left\{\frac{1}{\sqrt{2\pi}\sigma}e^{-\frac{\left(x-(\beta_0+h(\boldsymbol{z})+\beta_g g+\beta_d+\beta_{gd}g)\right)^2}{2\sigma^2}}\right\}}{\frac{1}{\sqrt{2\pi}\sigma}e^{-\frac{\left(x+1-(\beta_0+h(\boldsymbol{z})+\beta_g g)\right)^2}{2\sigma^2}} \Big/ \left\{\frac{1}{\sqrt{2\pi}\sigma}e^{-\frac{\left(x-(\beta_0+h(\boldsymbol{z})+\beta_g g)\right)^2}{2\sigma^2}}\right\}} \\
&= e^{(\beta_d+\beta_{gd}g)/\sigma^2}.
\end{aligned}$$

As a result, $\mathrm{ROR} = \frac{\mathrm{OR}(D|X,G=1,\boldsymbol{Z}=z)}{\mathrm{OR}(D|X,G=0,\boldsymbol{Z}=z)} = e^{\frac{\beta_{gd}}{\sigma^2}}$.

Appendix B: Inference about the ROR based on linear model (2)

From Appendix A and linear model (2), the log(ROR) can be estimated by $\hat{\beta}_{gd}/\hat{\sigma}^2$, where $\hat{\beta}_{gd}$ is obtained by the standard OLS theory and $\hat{\sigma}^2$ by the empirical variance of the residuals in model (2). We now use subscript i to index the ith subject. Specifically, $h(\boldsymbol{Z}_i)$ can be represented as $\tilde{\boldsymbol{Z}}_i^T\boldsymbol{\beta_z}$, where $\tilde{\boldsymbol{Z}}_i$ is a vector containing all the variables generated from $\boldsymbol{Z}_i$, including all spline terms. With n subjects, linear model (2) can be rewritten as $\boldsymbol{X} = \boldsymbol{M}\boldsymbol{\beta}+\boldsymbol{\epsilon}$, where $\boldsymbol{X}$ is a n-column vector containing all subjects' environmental variables, $\boldsymbol{M}$ is a $n \times p$ design matrix with the ith row $[1, \tilde{\boldsymbol{Z}}_i^T, G_i, D_i, G_iD_i]^T$, $\boldsymbol{\beta} = [\beta_0, \boldsymbol{\beta_z}, \boldsymbol{\beta}_g, \beta_d, \beta_{gd}]^T$ is the unknown $p \times 1$ parameters. Then, $\hat{\beta}_{gd}$ is the last element of $\hat{\boldsymbol{\beta}} = (\boldsymbol{M}^T\boldsymbol{M})^{-1}\boldsymbol{M}^T\boldsymbol{X}$, and $\hat{\sigma}^2 = \frac{S^2}{n-p}$, where S^2 is the sum of the squared error, i.e., $S^2 = ||\boldsymbol{X} - \boldsymbol{M}\hat{\boldsymbol{\beta}}||^2$. The variance of $\hat{\beta}_{gd}/\hat{\sigma}^2$ can be derived based on the delta method; that is, $\mathrm{var}(\hat{\beta}_{gd}/\hat{\sigma}^2) = [\frac{1}{\hat{\sigma}^2}, -\frac{\hat{\beta}_{gd}}{\hat{\sigma}^4}]\widehat{\boldsymbol{\Sigma}}[\frac{1}{\hat{\sigma}^2}, -\frac{\hat{\beta}_{gd}}{\hat{\sigma}^4}]^T$, where $\boldsymbol{\Sigma}$ is the variance-covariance matrix of $[\hat{\beta}_{gd}, \hat{\sigma}^2]^T$.

We can use the sandwich method to estimate variance-covariance matrix $\boldsymbol{\Sigma}$, based on the

following system of estimating equation:

$$\begin{cases} \boldsymbol{M}^T(\boldsymbol{X} - \boldsymbol{M}\boldsymbol{\beta}) = \boldsymbol{0}, \\ \frac{(\boldsymbol{X}-\boldsymbol{M}\boldsymbol{\beta})^T(\boldsymbol{X}-\boldsymbol{M}\boldsymbol{\beta})}{n-p} - \sigma^2 = 0. \end{cases}$$

The point estimate and 95% confidence interval of ROR can be obtained by $\exp(\hat{\beta}_{gd}/\hat{\sigma}^2)$ and $\exp\left(\hat{\beta}_{gd}/\hat{\sigma}^2 \pm 1.96 \times \sqrt{\widehat{\text{var}}(\hat{\beta}_{gd}/\hat{\sigma}^2)}\right)$ respectively.

Appendix C: Validity of the reverse test under a case-control study design.

Consider a source population for which we can use the reverse approach to specify the conditional distribution $f(X|D, G, \boldsymbol{Z})$, and a case-control study is formed based on this source population. The sampling probability for selecting a subject into a case-control study does not depend on the exposure X, mathematically, we have that $Pr(I = 1|D, X, G, \boldsymbol{Z}) = Pr(I = 1|D, G, \boldsymbol{Z})$, where I is an indicator that equals 1 if this subject is selected into the case-control study and 0 otherwise. In the derived case-control study, the reverse test specifies the following distribution $f(X|D, G, \boldsymbol{Z}, I = 1)$. By Bayes' formula, we have that

$$\begin{aligned} f(X|D, G, \boldsymbol{Z}, I = 1) &= \frac{f(X, I = 1|D, G, \boldsymbol{Z})}{P(I = 1|D, G, \boldsymbol{Z})}, \\ &= \frac{P(I = 1|X, D, G, \boldsymbol{Z}) f(X|D, G, \boldsymbol{Z})}{P(I = 1|D, G, \boldsymbol{Z})}, \\ &= \frac{P(I = 1|D, G, \boldsymbol{Z}) f(X|D, G, \boldsymbol{Z})}{P(I = 1|D, G, \boldsymbol{Z})}, \\ &= f(X|D, G, \boldsymbol{Z}). \end{aligned}$$

This indicates $f(X|D, G, \boldsymbol{Z}, I = 1) = f(X|D, G, \boldsymbol{Z})$ and therefore the the case-control sampling scheme does not affect the validity of the reverse test.

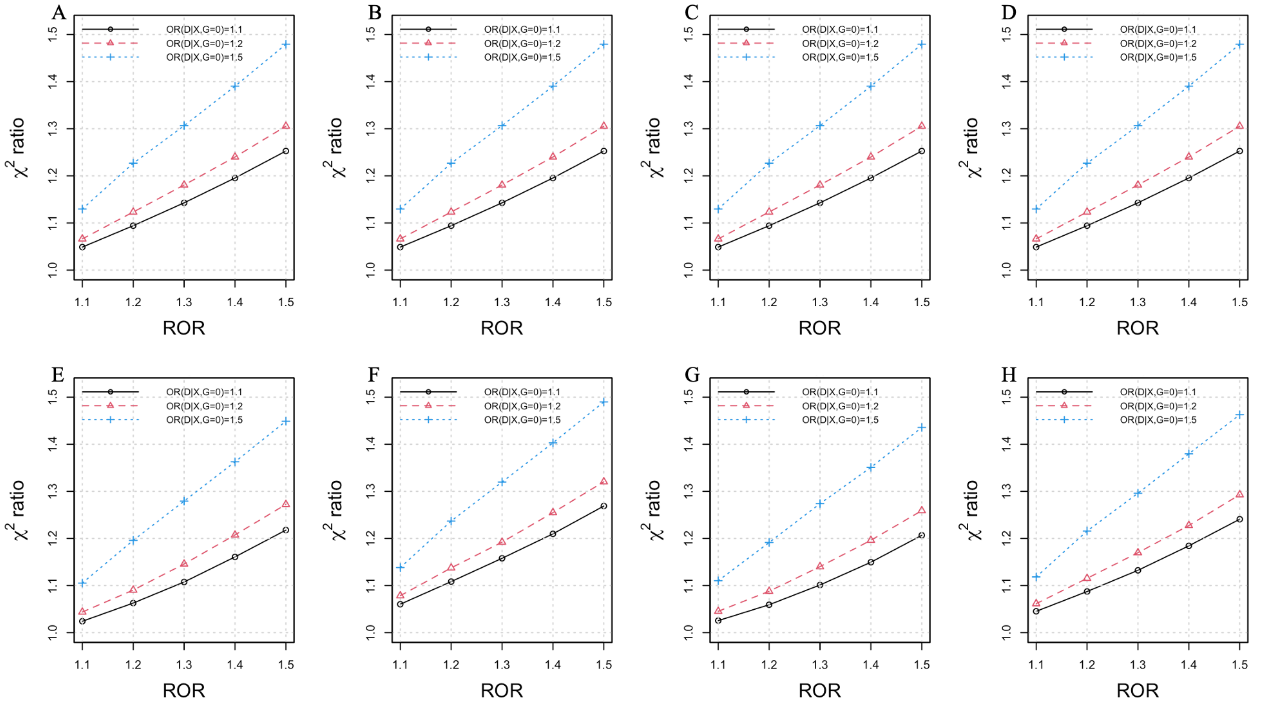

Figure 1: Power comparison between the reverse test and logistic regression test, where the x-axis is the ROR representing the magnitude of the GxE interaction, and the y-axis is the average χ^2 ratio of the reverse test against the logistic regression test. The results were calculated through 500,000 simulations, with increasing the ROR and main effect of X (i.e OR($X|G=0$)) from 1.1 to 1.5. Left column: weak versus strong exposure-confounder association, of Corr$(X, Z_2|Z_1 = D = G = 0) = 0.01$ (panel A) and 0.2 (panel E); Second column: weak versus strong disease-gene association, OR$(G|X = 0)$= 1.1 (panel B) or 1.5 (panel F); Third column: weak versus strong gene-exposure correlation, of Corr$(X, G|Z_1 = Z_2 = D = 0)$=0.01 (panel C) or 0.2 (panel G); Right column: rare versus common disease prevalence, of $Pr(D = 1|G = X = Z_1 = Z_2 = 0)$=0.05 (panel D) or 0.2 (panel H).

Table 1: Type I error rates of the reverse test and logistic regression test (with $ROR = 1$, i.e., no GxE interaction effect)

$P_0(D=1)$	$\mathrm{OR}(D\|G,X=0)$	$\mathrm{Corr}_0(X,Z_2)$	$\mathrm{Corr}_0(X,G)$	$\mathrm{OR}(D\|X,G=0)$=1.1				$\mathrm{OR}(D\|X,G=0)$=1.5			
				X		$X^*(\rho=0.25)$		X		$X^*(\rho=0.25)$	
				logistic	reverse	logistic	reverse	logistic	reverse	logistic	reverse
Simulation under 5% type I error threshold											
0.05	1.1	0.01	0.01	0.050	0.050	0.05	0.050	0.050	0.050	0.050	0.050
			0.2	0.050	0.050	0.05	0.050	**0.049**	0.050	0.050	0.049
		0.2	0.01	0.050	0.050	0.05	0.050	0.050	0.050	0.050	0.050
			0.2	0.050	0.050	0.05	0.049	0.050	0.050	0.050	0.050
	1.5	0.01	0.01	0.050	**0.051**	0.05	0.050	0.050	0.050	0.050	0.050
			0.2	0.050	0.050	0.05	0.050	0.050	0.050	0.050	0.050
		0.2	0.01	0.050	0.050	0.05	0.050	0.050	0.050	0.050	0.050
			0.2	0.050	0.050	0.05	0.050	0.050	0.050	0.050	0.051
0.20	1.1	0.01	0.01	0.050	0.050	0.05	0.050	0.050	0.050	0.050	0.050
			0.2	0.050	0.050	0.05	0.050	0.050	0.050	0.050	0.050
		0.2	0.01	0.051	0.050	0.05	0.050	0.050	0.050	**0.051**	0.050
			0.2	0.050	0.050	0.05	0.050	0.050	0.050	0.051	0.050
	1.5	0.01	0.01	0.050	0.050	0.05	0.050	0.050	0.050	0.050	0.050
			0.2	0.050	0.050	0.05	0.050	0.050	0.051	0.050	0.050
		0.2	0.01	0.050	0.050	0.05	0.050	**0.051**	0.051	0.050	**0.051**
			0.2	0.051	0.050	0.05	0.050	0.050	0.049	0.051	0.050
0.50	1.1	0.01	0.01	0.050	0.050	0.050	0.050	0.050	0.050	0.050	0.050
			0.2	0.050	0.049	0.050	0.050	0.050	0.050	0.050	0.050
		0.2	0.01	0.050	0.050	0.050	0.050	0.050	0.050	0.050	0.050
			0.2	0.050	0.050	0.050	0.050	0.050	**0.049**	0.050	0.050
	1.5	0.01	0.01	0.050	0.050	0.050	0.050	0.050	**0.049**	0.050	0.050
			0.2	0.050	0.050	0.050	0.050	0.050	0.050	0.050	0.050
		0.2	0.01	0.050	0.050	0.050	0.050	0.050	**0.049**	0.050	0.050
			0.2	0.051	0.051	0.050	0.050	0.050	0.050	0.050	0.050
Simulation under 0.01% type I error threshold											
0.05	1.1	0.01	0.01	9.8e-05	1.1e-04	1.0e-04	1.1e-04	8.8e-05	1.2e-04	7.8e-05	8.4e-05
			0.2	**5.8e-05**	9.0e-05	9.2e-05	8.8e-05	8.4e-05	8.4e-05	1.0e-04	1.2e-04
		0.2	0.01	7.8e-05	1.0e-04	7.4e-05	9.2e-05	7.6e-05	**1.5e-04**	**6.0e-05**	**7.0e-05**
			0.2	9.8e-05	9.8e-05	8.4e-05	1.1e-04	8.0e-05	9.8e-05	1.0e-04	**1.4e-04**
	1.5	0.01	0.01	8.2e-05	8.4e-05	9.8e-05	1.2e-04	1.1e-04	1.1e-04	8.6e-05	1.0e-04
			0.2	9.2e-05	1.2e-04	7.6e-05	1.1e-04	9.0e-05	8.4e-05	8.6e-05	1.0e-04
		0.2	0.01	1.0e-04	1.0e-04	8.0e-05	1.2e-04	1.0e-04	1.1e-04	9.0e-05	1.2e-04
			0.2	1.0e-04	8.6e-05	**5.8e-05**	**6.4e-05**	9.6e-05	1.1e-04	8.4e-05	8.0e-05
0.20	1.1	0.01	0.01	9.0e-05	1.2e-04	8.8e-05	1.2e-04	**7.2e-05**	8.4e-05	9.0e-05	1.1e-04
			0.2	9.4e-05	1.1e-04	7.8e-05	1.2e-04	1.2e-04	1.2e-04	1.0e-04	1.2e-04
		0.2	0.01	8.6e-05	9.6e-05	8.4e-05	1.0e-04	9.6e-05	1.1e-04	**7.0e-05**	1.1e-04
			0.2	9.6e-05	1.2e-04	8.2e-05	9.4e-05	8.6e-05	1.2e-04	7.4e-05	1.1e-04
	1.5	0.01	0.01	8.2e-05	7.8e-05	7.6e-05	9.0e-05	1.0e-04	9.8e-05	**7.2e-05**	8.6e-05
			0.2	8.6e-05	9.8e-05	**7.2e-05**	8.0e-05	7.8e-05	1.3e-04	1.1e-04	1.1e-04
		0.2	0.01	9.4e-05	9.2e-05	7.8e-05	1.0e-04	1.0e-04	1.2e-04	1.0e-04	1.2e-04
			0.2	8.0e-05	7.6e-05	8.2e-05	1.1e-04	8.4e-05	1.0e-04	9.2e-05	9.8e-05
0.50	1.1	0.01	0.01	**6.8e-05**	8.8e-05	7.8e-05	8.4e-05	9.0e-05	9.0e-05	**7.2e-05**	9.6e-05
			0.2	**6.0e-05**	7.4e-05	7.6e-05	1.0e-04	**6.2e-05**	9.6e-05	7.8e-05	1.0e-04
		0.2	0.01	8.2e-05	9.2e-05	7.8e-05	1.0e-04	1.0e-04	1.1e-04	1.2e-04	1.1e-04
			0.2	8.4e-05	1.0e-04	9.8e-05	1.2e-04	1.0e-04	1.0e-04	**6.8e-05**	9.0e-05
	1.5	0.01	0.01	8.2e-05	1.1e-04	8.2e-05	1.1e-04	8.0e-05	8.2e-05	**7.2e-05**	9.2e-05
			0.2	8.0e-05	9.2e-05	8.0e-05	9.0e-05	1.1e-04	1.0e-04	8.2e-05	1.1e-04
		0.2	0.01	8.8e-05	9.0e-05	1.0e-04	1.1e-04	1.0e-04	1.1e-04	8.2e-05	8.0e-05
			0.2	8.2e-05	9.6e-05	9.8e-05	1.1e-04	1.1e-04	1.1e-04	9.2e-05	1.2e-04

NOTE: In this table, “logistic" and “reverse" represent the logistic regression test and reverse test respectively. $P_0(D=1)$, $\mathrm{Corr}_0(X,Z_2)$, and $\mathrm{Corr}_0(X,G)$ denote $Pr(D=1|Z_1=Z_2=G=D=0)$, $\mathrm{Corr}(X,Z_2|Z_1=G=D=0)$ and $\mathrm{Corr}(X,G|Z_1=Z_2=G=D=0)$ respectively. Here, $\rho=\frac{\mathrm{Var}(X)}{\mathrm{Var}(X^*)}$ denotes the magnitude of the measurement error, where X is the true exposure and X^* is the observed exposure measured with error. The empirical type I error rates were calculated across 500,000 simulations for each scenario, where the empirical type I error rates outside the 95% confidence boundary, i.e., $p \pm 1.96 \times \sqrt{\frac{p(1-p)}{B}}$, were highlighted in bold. Here, p denotes the nominal threshold (5% or 0.01%) and B denotes number of replication (500,000).

Table 2: Type I error rates at 0.01% significant level of the reverse test and logistic regression test, in Scenario I that $P(D = 1|G, \boldsymbol{Z})$ follows a simple logistic regression model and the logistic regression model and the linear model do not hold simultaneously.

$P_0(D=1)$	$\text{OR}(D\|G,X=0)$	$\text{Corr}_0(X,Z_2)$	$\text{Corr}_0(X,G)$	$\text{OR}(D\|,X,G=0)$=1.1				$\text{OR}(D\|X,G=0)$=1.5			
				X		$X^*(\rho=0.25)$		X		$X^*(\rho=0.25)$	
				logistic	reverse	logistic	reverse	logistic	reverse	logistic	reverse
Linear data generation procedure											
0.05	1.1	0.01	0.01	7.8e-05	9.6e-05	**7.2e-05**	9.8e-05	**6.2e-05**	8.8e-05	7.8e-05	1.1e-04
			0.2	9.6e-05	1.1e-04	**6.6e-05**	1.0e-04	8.2e-05	1.0e-04	8.6e-05	8.2e-05
		0.2	0.01	9.6e-05	1.2e-04	8.6e-05	1.2e-04	8.4e-05	9.6e-05	8.4e-05	1.0e-04
			0.2	8.6e-05	7.4e-05	7.4e-05	1.1e-04	1.1e-04	1.2e-04	**7.2e-05**	9.2e-05
	1.5	0.01	0.01	**5.8e-05**	8.4e-05	**7.0e-05**	8.6e-05	9.4e-05	1.2e-04	9.0e-05	1.0e-04
			0.2	8.2e-05	8.8e-05	8.2e-05	**1.4e-04**	1.1e-04	**1.4e-04**	1.1e-04	1.2e-04
		0.2	0.01	7.8e-05	9.2e-05	**6.0e-05**	7.8e-05	9.8e-05	1.0e-04	8.2e-05	8.0e-05
			0.2	7.8e-05	1.0e-04	1.0e-04	1.0e-04	1.0e-04	1.1e-04	8.4e-05	8.6e-05
0.2	1.1	0.01	0.01	7.6e-05	1.0e-04	9.0e-05	1.1e-04	8.0e-05	1.0e-04	8.0e-05	9.0e-05
			0.2	1.1e-04	1.1e-04	9.0e-05	9.0e-05	1.0e-04	1.1e-04	1.1e-04	1.0e-04
		0.2	0.01	**7.2e-05**	1.2e-04	1.1e-04	1.1e-04	1.1e-04	1.0e-04	1.0e-04	1.1e-04
			0.2	7.8e-05	9.6e-05	**6.4e-05**	1.0e-04	**7.2e-05**	8.8e-05	8.4e-05	8.4e-05
	1.5	0.01	0.01	7.6e-05	9.6e-05	9.8e-05	**1.3e-04**	7.6e-05	9.6e-05	1.2e-04	**1.3e-04**
			0.2	7.8e-05	9.0e-05	8.6e-05	1.0e-04	8.4e-05	9.4e-05	7.6e-05	1.2e-04
		0.2	0.01	8.4e-05	9.0e-05	7.6e-05	8.8e-05	9.0e-05	9.8e-05	8.6e-05	1.1e-04
			0.2	7.8e-05	1.1e-04	**5.4e-05**	7.6e-05	8.2e-05	1.2e-04	9.8e-05	1.2e-04
0.5	1.1	0.01	0.01	1.1e-04	1.0e-04	**6.8e-05**	9.0e-05	**6.8e-05**	8.4e-05	**7.0e-05**	9.0e-05
			0.2	8.6e-05	1.0e-04	9.0e-05	9.4e-05	8.0e-05	1.2e-04	8.0e-05	9.6e-05
		0.2	0.01	8.0e-05	1.1e-04	8.0e-05	**7.0e-05**	8.4e-05	9.8e-05	**6.8e-05**	1.0e-04
			0.2	8.8e-05	9.6e-05	8.6e-05	8.8e-05	**7.0e-05**	1.1e-04	7.4e-05	9.0e-05
	1.5	0.01	0.01	7.8e-05	1.1e-04	7.4e-05	9.0e-05	8.6e-05	1.0e-04	8.6e-05	1.1e-04
			0.2	8.8e-05	9.0e-05	**7.2e-05**	9.6e-05	**6.0e-05**	1.1e-04	1.0e-04	1.0e-04
		0.2	0.01	8.2e-05	8.4e-05	1.0e-04	9.0e-05	8.2e-05	9.4e-05	8.0e-05	1.1e-04
			0.2	9.2e-05	1.1e-04	**5.4e-05**	8.4e-05	9.0e-05	1.0e-04	9.2e-05	1.0e-04
Logistic data generation procedure											
0.05	1.1	0.01	0.01	7.6e-05	9.4e-05	9.2e-05	1.0e-04	7.6e-05	1.0e-04	9.6e-05	9.8e-05
			0.2	8.2e-05	9.2e-05	8.0e-05	9.6e-05	8.2e-05	1.1e-04	7.6e-05	8.4e-05
		0.2	0.01	**7.2e-05**	1.1e-04	**6.2e-05**	7.8e-05	8.6e-05	1.1e-04	**6.6e-05**	9.8e-05
			0.2	7.6e-05	1.1e-04	8.8e-05	1.0e-04	7.6e-05	9.8e-05	8.4e-05	8.8e-05
	1.5	0.01	0.01	7.8e-05	1.1e-04	7.4e-05	8.4e-05	**6.0e-05**	9.0e-05	9.2e-05	**7.2e-05**
			0.2	9.4e-05	1.2e-04	8.8e-05	1.0e-04	7.8e-05	9.6e-05	8.2e-05	1.0e-04
		0.2	0.01	7.4e-05	9.6e-05	8.0e-05	1.1e-04	**6.2e-05**	9.2e-05	8.8e-05	1.1e-04
			0.2	9.0e-05	1.2e-04	1.1e-04	1.1e-04	8.0e-05	9.6e-05	1.0e-04	1.1e-04
0.2	1.1	0.01	0.01	8.0e-05	1.2e-04	7.4e-05	9.4e-05	9.8e-05	1.0e-04	7.4e-05	8.2e-05
			0.2	1.0e-04	1.2e-04	8.2e-05	9.4e-05	8.6e-05	1.0e-04	9.0e-05	**1.3e-04**
		0.2	0.01	7.6e-05	9.8e-05	7.6e-05	1.1e-04	8.6e-05	1.0e-04	8.0e-05	8.6e-05
			0.2	8.6e-05	1.1e-04	9.0e-05	9.2e-05	8.8e-05	1.0e-04	**5.2e-05**	9.4e-05
	1.5	0.01	0.01	8.8e-05	1.1e-04	8.8e-05	1.1e-04	**6.4e-05**	8.8e-05	8.2e-05	8.8e-05
			0.2	1.0e-04	1.0e-04	**7.0e-05**	9.4e-05	**5.8e-05**	**6.6e-05**	7.8e-05	**1.4e-04**
		0.2	0.01	**7.0e-05**	8.8e-05	7.6e-05	1.0e-04	9.2e-05	1.0e-04	1.1e-04	9.6e-05
			0.2	**6.8e-05**	1.0e-04	8.2e-05	1.0e-04	8.4e-05	1.1e-04	8.8e-05	1.2e-04
0.5	1.1	0.01	0.01	8.4e-05	9.0e-05	8.2e-05	1.0e-04	1.0e-04	8.8e-05	8.4e-05	9.4e-05
			0.2	9.4e-05	1.1e-04	7.4e-05	9.2e-05	7.6e-05	**1.4e-04**	8.2e-05	1.2e-04
		0.2	0.01	8.8e-05	1.1e-04	7.6e-05	9.2e-05	1.0e-04	1.0e-04	**6.8e-05**	8.4e-05
			0.2	9.4e-05	1.1e-04	8.0e-05	9.2e-05	7.6e-05	9.4e-05	**6.0e-05**	**6.4e-05**
	1.5	0.01	0.01	8.6e-05	1.0e-04	8.6e-05	1.0e-04	**6.0e-05**	9.2e-05	7.6e-05	1.1e-04
			0.2	9.0e-05	9.2e-05	8.0e-05	9.2e-05	**7.2e-05**	8.6e-05	9.8e-05	8.4e-05
		0.2	0.01	**7.2e-05**	1.0e-04	**7.2e-05**	**6.6e-05**	7.6e-05	8.4e-05	7.6e-05	8.6e-05
			0.2	8.8e-05	1.1e-04	8.6e-05	1.0e-04	8.8e-05	1.2e-04	8.4e-05	9.8e-05

NOTE: In this table, "logistic" and "reverse" represent the logistic regression test and reverse test respectively. $P_0(D=1)$, $\text{Corr}_0(X,Z_2)$, and $\text{Corr}_0(X,G)$ denote $Pr(D=1|Z_1=Z_2=G=D=0)$, $\text{Corr}(X,Z_2|Z_1=G=D=0)$ and $\text{Corr}(X,G|Z_1=Z_2=G=D=0)$ respectively. Here, $\rho = \frac{\text{Var}(X)}{\text{Var}(X^*)}$ denotes the magnitude of the measurement error, where X is the true exposure and X^* is the observed exposure measured with error. The empirical type I error rates were calculated across 500,000 simulations for each scenario, where the empirical type I error rates outside the 95% confidence boundary, i.e., $p \pm 1.96 \times \sqrt{\frac{p(1-p)}{B}}$, were highlighted in bold. Here, p denotes the significance level (0.01%) and B denotes number of replication (500,000).

Table 3: Relative power of the reverse test against logistic regression test, where ROR=1.5.

				χ^2 ratio							
$P_0(D=1)$	$\text{OR}(D \mid G, X=0)$	$\text{Corr}_0(X, Z_2)$	$\text{Corr}_0(X, G)$	$\text{OR}(D \mid X, G=0)$=1.1				$\text{OR}(D \mid X, G=0)$=1.5			
				$\rho=1$	$\rho=0.75$	$\rho=0.5$	$\rho=0.25$	$\rho=1$	$\rho=0.75$	$\rho=0.5$	$\rho=0.25$
0.05	1.1	0.01	0.01	1.25	1.19	1.14	1.08	1.48	1.36	1.25	1.14
			0.2	1.21	1.13	1.06	0.99	1.43	1.30	1.18	1.07
		0.2	0.01	1.22	1.15	1.08	1.02	1.45	1.32	1.20	1.09
			0.2	1.17	1.09	1.01	0.94	1.40	1.27	1.14	1.02
	1.5	0.01	0.01	1.27	1.21	1.16	1.10	1.49	1.38	1.27	1.16
			0.2	1.23	1.16	1.09	1.02	1.47	1.34	1.22	1.11
		0.2	0.01	1.23	1.17	1.11	1.04	1.46	1.34	1.22	1.12
			0.2	1.21	1.12	1.04	0.97	1.44	1.31	1.18	1.07
0.20	1.1	0.01	0.01	1.24	1.18	1.13	1.07	1.46	1.35	1.24	1.13
			0.2	1.19	1.12	1.05	0.98	1.42	1.29	1.17	1.06
		0.2	0.01	1.21	1.14	1.08	1.02	1.44	1.31	1.19	1.08
			0.2	1.17	1.09	1.01	0.94	1.39	1.26	1.14	1.02
	1.5	0.01	0.01	1.25	1.20	1.14	1.09	1.47	1.36	1.25	1.15
			0.2	1.22	1.15	1.08	1.02	1.44	1.32	1.20	1.10
		0.2	0.01	1.22	1.16	1.10	1.04	1.44	1.32	1.21	1.11
			0.2	1.19	1.12	1.04	0.97	1.42	1.29	1.17	1.06
0.50	1.1	0.01	0.01	1.23	1.17	1.12	1.06	1.45	1.34	1.23	1.12
			0.2	1.19	1.12	1.05	0.98	1.41	1.28	1.17	1.06
		0.2	0.01	1.20	1.14	1.07	1.01	1.43	1.30	1.19	1.08
			0.2	1.16	1.08	1.01	0.94	1.39	1.25	1.13	1.03
	1.5	0.01	0.01	1.24	1.19	1.14	1.08	1.45	1.34	1.24	1.14
			0.2	1.21	1.14	1.08	1.02	1.42	1.30	1.19	1.09
		0.2	0.01	1.21	1.15	1.09	1.03	1.42	1.31	1.20	1.10
			0.2	1.18	1.11	1.04	0.98	1.39	1.27	1.16	1.06

NOTE: $P_0(D=1)$, $\text{Corr}_0(X, Z_2)$, and $\text{Corr}_0(X, G)$ denote $Pr(D=1 \mid Z_1 = Z_2 = G = D = 0)$, $\text{Corr}(X, Z_2 \mid Z_1 = G = D = 0)$ and $\text{Corr}(X, G \mid Z_1 = Z_2 = G = D = 0)$ respectively. Here, $\rho = \frac{\text{Var}(X)}{\text{Var}(X^*)}$ denotes the magnitude of the measurement error, where X is the true exposure and X^* is the observed exposure measured with error. The relative power was calculated by $\frac{\bar{\chi}^2_{\text{rev}}}{\bar{\chi}^2_{\text{log}}}$ where $\bar{\chi}^2_{\text{rev}}$ and $\bar{\chi}^2_{\text{rev}}$ are the average χ^2 statistics for the logistic regression test and reverse test based on 500,000 simulation repetitions for each scenario.

Table 4: Comparison of computation time

Sample Size	Computation time in hours		
(Cases+Controls)	reverse test	logistic regression test	ratio
1,000	0.34	1.19	3.54
2,000	0.44	2.10	4.78
10,000	2.76	20.64	7.47
40,000	5.47	41.18	7.53
200,000	30.79	236.58	7.68

Note: We compared the computation time for the reverse and logistic regression test for GxE interaction, in hours for 1,000,000 SNPs.

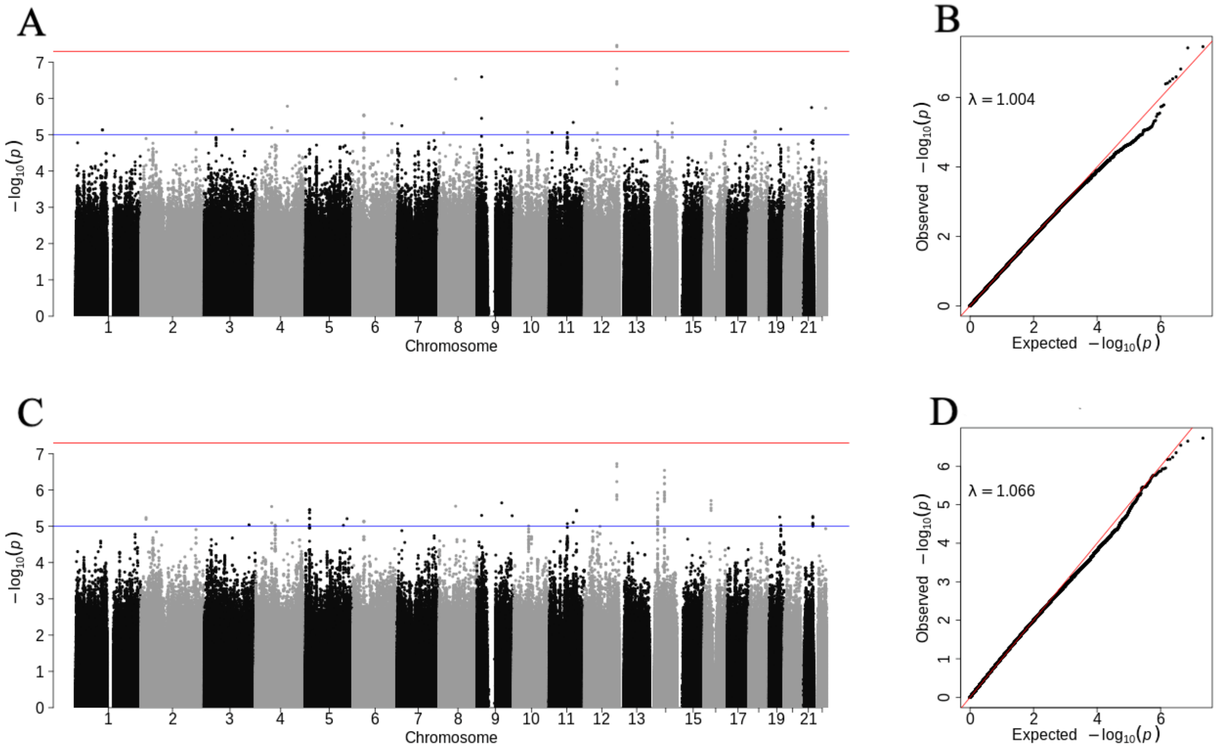

Figure 2: Manhattan plots and Quantile-Quantile plots for the test of interaction effect between 10,079,672 common SNPs and smoking (cigarettes per day) using the reverse test (upper panel) and standard logistic regression test (lower panel). In the Quantile-Quantile plots, λ denotes the genomic inflation factor. Red line: genome-wide significance level (P-value= 5×10^{-8}). Blue line: suggestive level (P-value= 1×10^{-5}).

Table 5: SNPs with P-value $< 1 \times 10^{-6}$ in at least one of the reverse and logistic regression test in the analysis gene-smoking interaction effects in relation to HIV infection (VACS Aferican Americans, n=1484). The smaller P-value between the two tests was highlighted in bold.

chromosome	gene	SNP name	major allele	minor allele	MAF	logistic test	reverse test
8	AC018953.1	rs72649207	C	G	0.08	2.79e-06	**2.88e-07**
9	SH3GL2	rs77236711	G	A	0.05	5.01e-06	**2.54e-07**
12	ZNF664	rs10773059	T	C	0.44	1.36e-06	**3.40e-07**
		rs10744166	T	C	0.46	2.25e-07	**3.71e-08**
		rs10773060	G	A	0.45	1.88e-07	**3.42e-08**
		rs10744167	C	T	0.44	5.87e-07	**1.51e-07**
		rs7315555	G	A	0.45	1.52e-06	**3.84e-07**
		rs7303161	T	C	0.45	1.82e-06	**4.03e-07**
14	SLC35F4	rs10145503	G	A	0.25	**4.47e-07**	4.08e-05
		rs10148287	G	A	0.25	**2.87e-07**	3.09e-05
		rs12432123	G	A	0.27	**6.60e-07**	4.46e-05
		rs7146231	T	C	0.27	**6.74e-07**	4.66e-05

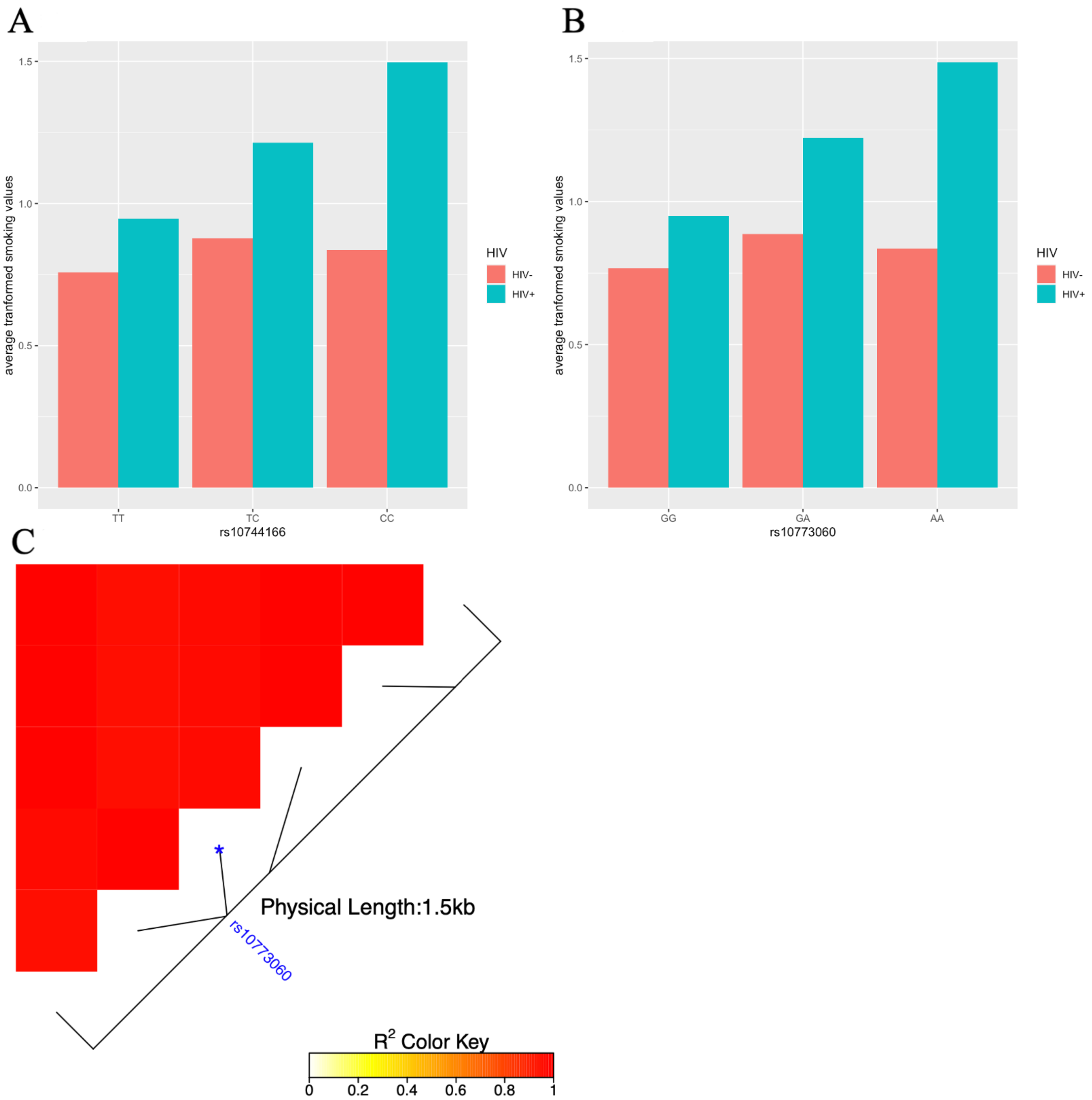

Figure 3: Summary of the interaction effects of rs10744166 and rs10773060 with smoking on HIV infection based on the reverse test. Panel A: average of the transformed smoking values (cigarettes per day) between HIV-infected and -uninfected veterans stratified by the 'TT', 'TC', and 'CC' genotypes of rs10744166. Panel B: average of the transformed smoking values (cigarettes per day) between HIV-infected and -uninfected veterans stratified by the 'GG', 'GA', and 'AA' genotypes of rs10773060. Panel C: Summary of the local linkage disequilibrium among SNPs located at gene *ZNP664* with P-value below 1×10^{-6}, where the SNPs from bottom to top are rs10773059, rs10744166, rs10773060, rs10744167, rs7315555, and rs7303161. The squared allele frequency correlations R^2 for each pair of SNPs were considered to estimate linkage disequilibrium.
