# Supplementary material for: Testing gene–environment interactions in the presence of confounders and mismeasured environmental exposures
Source: G3 (Bethesda). 2021 Jul 16;11(10):jkab236. doi: 10.1093/g3journal/jkab236 (PMC8473983; doi:10.1093/g3journal/jkab236)
Supplement: jkab236_Supplementary_Data [file jkab236_supplementary_data.zip › jkab236-suppl_data/GENETICS-G3-2021-402584-s02.zip!/Supplementary Material.pdf]

Supplementary Material: Testing Gene-Environment Interactions in the Presence of Confounders and Mismeasured Environmental Exposures

Chao Cheng[†,‡], Donna Spiegelman[†,‡], Zuoheng Wang[†], and Molin Wang[*§,††]

[†]Department of Biostatistics, Yale School of Public Health, New Haven, CT, 06510, USA

[‡]Center for Methods in Implementation and Prevention Science, Yale School of Public Health, New Haven, CT, 06510, USA

[§]Departments of Biostatistics and Epidemiology, Harvard T.H. Chan School of Public Health, Boston, MA, 02115, USA

[††]Channing Division of Network Medicine, Department of Medicine, Brigham and Women's Hospital, Harvard Medical School, Boston, MA, 02115, USA

[*]Corresponding to: Departments of Biostatistics and Epidemiology, Harvard T.H. Chan School of Public Health, Boston, MA, 02115, USA. E-mail: stmow@channing.harvard.edu

Supplementary Material Appendix

Supplementary Material Appendix A: Derivation of ROR based on the linear model (2) in the presence of Z-G and Z-D interaction terms

We can separate $h(\boldsymbol{Z}^{**})$ into a summation of the following 3 parts, $\tilde{h}_1(\boldsymbol{Z})$, $\tilde{h}_2(\boldsymbol{Z}G)$ and $\tilde{h}_3(\boldsymbol{Z}D)$, representing the main effect of $\boldsymbol{Z}$, and Z-G and Z-D interaction effects, respectively. From model (2) with $h(\boldsymbol{Z})$ replaced by $h(\boldsymbol{Z}^{**})$, the p.d.f of X conditional on $\boldsymbol{Z} = \boldsymbol{z}$, $D = d$ and $G = g$ is $f(X = x|\boldsymbol{Z} = \boldsymbol{z}, D = d, G = g) = \frac{1}{\sqrt{2\pi}\sigma} \exp\left\{ -\frac{(x-\beta_0-\tilde{h}_1(\boldsymbol{z})-\tilde{h}_2(\boldsymbol{z}g)-\tilde{h}_3(\boldsymbol{z}d)-\beta_g g-\beta_d d-\beta_{gd} gd)^2}{2\sigma^2} \right\}$. Plugging this p.d.f into (9) and noticing that $\tilde{h}_3(\boldsymbol{z}d)$ degenerates to 0 when $d = 0$, we have $\mathrm{OR}(D|X, G = g, \boldsymbol{Z} = \boldsymbol{z}) = \exp(\frac{\tilde{h}_3(\boldsymbol{z})+\beta_d+\beta_{gd} g}{\sigma^2})$ for $g = 0, 1$. It is straightforward to show that $\mathrm{ROR} = \frac{\mathrm{OR}(D|X,G=1,\boldsymbol{Z}=\boldsymbol{z})}{\mathrm{OR}(D|X,G=0,\boldsymbol{Z}=\boldsymbol{z})} = \exp(\beta_{gd}/\sigma^2)$.

Supplementary Material Appendix B: The parametric relationship between the linear and logistic regression models (1) and (3)

We show that the logistic regression model (1) and linear model (3) can hold simultaneously, and that $\alpha_x = \beta_d/\sigma^2$ and $\alpha_{gx} = \beta_{gd}/\sigma^2$ when both the linear and logistic regression models hold under certain conditions to be specified. First, we assume the logistic regression model (1) holds and $X|D, G, \boldsymbol{Z}$ follows a normal distribution with mean $E(X|D, G, \boldsymbol{Z}) = g_1 + g_2 D$ and variance σ^2, where g_1 and g_2 depend on G and $\boldsymbol{Z}$. We now derive the specific forms of g_1 and g_2. According to Bayes' formula, $Pr(D = 1|G, X, \boldsymbol{Z})$ can be written as

$$Pr(D = 1|G, X, \boldsymbol{Z}) = \frac{f(X|D = 1, G, \boldsymbol{Z})Pr(D = 1|G, \boldsymbol{Z})}{f(X|D = 1, G, \boldsymbol{Z})Pr(D = 1|G, \boldsymbol{Z}) + f(X|D = 0, G, \boldsymbol{Z})Pr(D = 0|G, \boldsymbol{Z})},$$

which can be further rewritten as

$$\frac{f(X|D = 0, G, \boldsymbol{Z})}{f(X|D = 1, G, \boldsymbol{Z})} = \frac{Pr(D = 1|G, \boldsymbol{Z})}{Pr(D = 0|G, \boldsymbol{Z})} \times \frac{Pr(D = 0|X, G, \boldsymbol{Z})}{Pr(D = 1|X, G, \boldsymbol{Z})}.$$

This is the equation connecting the logistic and the linear regression models. Plugging in the density functions of $f(X|D, G, \boldsymbol{Z})$ and model (1), and taking logarithm on both sides of this equation, we have

$$
\begin{aligned}
-\frac{(X-g_1)^2}{2\sigma^2}+\frac{(X-g_1-g_2)^2}{2\sigma^2} &= \text{logit}(Pr(D=1|G,\boldsymbol{Z}))-(\alpha_0+m(\boldsymbol{Z})+\alpha_x X+\alpha_g G+\alpha_{gx}GX),\\
-\frac{g_2}{\sigma^2}X+\frac{2(g_1+g_2)h_2}{2\sigma^2} &= -(\alpha_x+\alpha_{gx}G)X+\Big\{\text{logit}(Pr(D=1|G,\boldsymbol{Z}))-(\alpha_0+m(\boldsymbol{Z})+\alpha_g G)\Big\}.
\end{aligned}
$$

Comparing the terms between the left and right hand sides of this equation, we have

$$
\begin{cases}
g_1 = & \frac{\text{logit}(Pr(D=1|G,\boldsymbol{Z}))-(\alpha_0+m(\boldsymbol{Z})+\alpha_g G)}{\alpha_x+\alpha_{gx}G}-\frac{\alpha_x+\alpha_{gx}G}{2}\sigma^2,\\
g_2 = & \alpha_x\sigma^2+\alpha_{gx}\sigma^2 G.
\end{cases}
$$

We rewrite g_1 as $g_1(\boldsymbol{Z}, G)$ since g_1 is a function of G and $\boldsymbol{Z}$. It follows that

$$
E(X|D,G,\boldsymbol{Z}) = g_1(\boldsymbol{Z},G)+\alpha_x\sigma^2 D+\alpha_{gx}\sigma^2 GD. \tag{S1}
$$

Noting that $Pr(D = 1|G, \boldsymbol{Z})$ in $g_1(\boldsymbol{Z}, G)$ has not been pre-specified, (S1) can be rewritten as $E(X|D, G, \boldsymbol{Z}) = \beta_0+g(\boldsymbol{Z}, G)+\beta_g G+\alpha_x\sigma^2 D+\alpha_{gx}\sigma^2 GD$. If G is binary, $g(\boldsymbol{Z}, G)$ can be written as $\tilde{h}(\boldsymbol{Z})+\tilde{\tilde{h}}(\boldsymbol{Z})G$, where $\tilde{h}(\boldsymbol{Z}) = g(\boldsymbol{Z}, 0)$ and $\tilde{\tilde{h}}(\boldsymbol{Z}) = g(\boldsymbol{Z}, 1) - g(\boldsymbol{Z}, 0)$; that is, if G is binary, model (S1) can be written as $E(X|D, G, \boldsymbol{Z}) = \beta_0 + \tilde{h}(\boldsymbol{Z}) + \tilde{\tilde{h}}(\boldsymbol{Z})G + \beta_g G + \alpha_x\sigma^2 D + \alpha_{gx}\sigma^2 GD$. Similarly, if G is ordinal, $g(\boldsymbol{Z}, G)$ can be written as $\tilde{h}(\boldsymbol{Z}) + \tilde{\tilde{h}}(\boldsymbol{Z})G+\tilde{\tilde{\tilde{h}}}(\boldsymbol{Z})G^2$, where $\tilde{h}(\boldsymbol{Z}) = g(\boldsymbol{Z}, 0)$, $\tilde{\tilde{h}}(\boldsymbol{Z}) = 2g(\boldsymbol{Z}, 1) - \frac{g(\boldsymbol{Z},2)-g(\boldsymbol{Z},0)}{2}$, and $\tilde{\tilde{\tilde{h}}}(\boldsymbol{Z}) = \frac{g(\boldsymbol{Z},2)-g(\boldsymbol{Z},0)}{2} - g(\boldsymbol{Z}, 1)$. Comparing the coefficients of terms containing D in expection (S1) and linear model (3), we have that the parametric relationships $\alpha_x = \beta_d/\sigma^2$ and $\alpha_{gx} = \beta_{gd}/\sigma^2$.

In general, the logistic regression model (1) and linear model (2) will not always hold simultaneously. In fact, comparing the terms in (S1) and (2) leads to the following condition for models (1) and (2) to hold simultaneously:

$$
\beta_0 + h(\boldsymbol{Z}) + \beta_g G = g_1(\boldsymbol{Z}, G),
$$

for any $h(\boldsymbol{Z})$ and $g_1(\boldsymbol{Z}, G)$. It follows that

$$
\begin{aligned}
\text{logit}(Pr(D=1|G,\boldsymbol{Z})) = \;& \tfrac{(\alpha_{gx}G+\alpha_x)^2\sigma^2}{2} + (\alpha_{gx}G+\alpha_x)(\beta_0 + h(\boldsymbol{Z}) + \beta_g G) + \alpha_0 + m(\boldsymbol{Z}) + \alpha_g G. \\
= \;& \underbrace{\Big(\alpha_0 + \alpha_x\beta_0 + \frac{\alpha_x^2\sigma^2}{2}\Big)}_{\text{intercept}} + \underbrace{(\alpha_g + \alpha_{gx}\beta_0 + \alpha_x\alpha_{gx}\sigma^2)G}_{G\text{-term}} + \underbrace{(\frac{\alpha_{gx}^2\sigma^2}{2} + \alpha_{gx}\beta_g)G^2}_{G^2\text{-term}} \\
& + \underbrace{\Big(\alpha_x h(\boldsymbol{Z}) + m(\boldsymbol{Z})\Big)}_{\boldsymbol{Z}\text{-term}} + \underbrace{\alpha_{gx}h(\boldsymbol{Z})G}_{\boldsymbol{Z}\text{-}G\text{ term}} \\
& = b_0 + b_1 G + b_2 G^2 + b_z(\boldsymbol{Z}) + b_{zg}(\boldsymbol{Z}, G)
\end{aligned}
\tag{S2}
$$

Therefore, when (S2) holds, model (1) and (2) can hold simultaneously, and the parametric relationships $\alpha_x = \beta_d/\sigma^2$ and $\alpha_{gx} = \beta_{gd}/\sigma^2$ still hold. Since it is unlikely for (S2) to hold, models (1) and (2) typically do not hold simultaneously. However, note that if there is no exposure main effect and no GxE interaction effect ($\alpha_x = \alpha_{gx} = 0$), formula (S2) becomes

$$\text{logit}(Pr(D=1|G,\boldsymbol{Z})) = \beta_0 + h(\boldsymbol{Z}) + \beta_g G.$$

Supplementary Material Appendix C: Difference in Correlation Coefficient

An alternative representation for GxE interactions involves correlation coefficients, $\text{Corr}(X, D|G, \boldsymbol{Z})$, the correlation coefficient between exposure and disease. Using the linear model (2), we have

$$
\begin{aligned}
\text{Corr}(X, D|G, \boldsymbol{Z}) &= \frac{\text{Cov}(X, D|G, \boldsymbol{Z})}{\sqrt{\text{Var}(X|G,\boldsymbol{Z})}\sqrt{\text{Var}(D|G,\boldsymbol{Z})}}, \\
&= \frac{\gamma^*_{xd,g}}{\text{Var}(D|G,\boldsymbol{Z})}\text{Cov}\Big(h(\boldsymbol{Z}) + \beta_g G + \beta_d D + \beta_{gd} GD + \epsilon, D|G,\boldsymbol{Z}\Big), \\
&= \gamma^*_{xd,g}(\beta_d + \beta_{gd} G),
\end{aligned}
$$

where $\gamma^*_{xd,g} = \sqrt{\text{Var}(D|G,\boldsymbol{Z})}/\sqrt{\text{Var}(X|G,\boldsymbol{Z})}$. Note that the above derivation did not use the conditional normality assumption but did require the constant variance assumption. The difference in exposure-disease correlation coefficient between the subgroups of $G = 1$ and $G = 0$, referred as *difference in correlation coefficient* (DCC), can also measure the magnitude of the GxE interaction. If DCC $= 0$, there is no exposure-disease correlation difference across genetic subgroups, and therefore no GxE interaction. If the X-G association is weak, $\text{Var}(X|G = g, \boldsymbol{Z}) \approx \text{Var}(X|\boldsymbol{Z})$ for $g = 0, 1$. Also, if the D-G association is of small magnitude, it

is likely $\text{Var}(D|G = g, \boldsymbol{Z}) \approx \text{Var}(D|\boldsymbol{Z})$ for $g = 0, 1$. Therefore, under the assumptions of weak X-G and D-G associations, it is more likely that $\gamma^*_{xd,g} \approx \gamma^*_{xd} = \sqrt{\text{Var}(D|\boldsymbol{Z})}/\sqrt{\text{Var}(X|\boldsymbol{Z})}$, and $\text{DCC} = \text{Corr}(X, D|G = 1, \boldsymbol{Z}) - \text{Corr}(X, D|G = 0, \boldsymbol{Z}) \approx \gamma^*_{xd}\beta_{gd}$. Since $\gamma^*_{x,d}$ is a constant which is always greater than zero, testing for $H_0 : \text{DCC} = 0$ is also valid through evaluating $H_0 : \beta_{gd} = 0$.

Supplementary Material Appendix D: Data generation procedure for the normally distributed environmental exposure

We generated two confounders, Z_1 and Z_2, where $Z_1 \sim N(0, 1)$ and Z_2 is a binary with $Pr(Z_2 = 1) = 0.3$. Then, given $\boldsymbol{Z} = [Z_1, Z_2]^T$, we simulated $G = \{0, 1, 2\}$ according to a multinomial logistic model with probabilities $Pr(G = g|\boldsymbol{Z}) = \frac{e^{U_g}}{\sum_{j=0}^{2} e^{U_j}}$, $g = 0, 1, 2$, where $U_0 = 1$, $U_1 = \log(0.4) + \log(1.2)Z_1 + \log(1.2)Z_2$ and $U_2 = \log(0.1) + \log(1.5)Z_1 + \log(1.5)Z_2$, leading the marginal probabilities $Pr(G = 1) \approx 0.27$, $Pr(G = 2) \approx 0.07$, $\text{Corr}(Z_1, G) = 0.12$, and $\text{Corr}(Z_2, G) = 0.06$. Next, we generated the exposure D given G and $\boldsymbol{Z}$ by

$$\text{logit}\Big(Pr(D = 1|G, \boldsymbol{Z})\Big) = \frac{(\alpha_x + \alpha_{gx}G)^2}{2} + \Big(\alpha_x + \alpha_{gx}G\Big)\Big(\beta_0 + \beta_1 Z_1 + \beta_2 Z_1^2 + \beta_3 Z_2 + \beta_g G\Big) + \alpha_0 + \alpha_1 Z_1 + \alpha_2 Z_1^2 + \alpha_3 Z_2 + \alpha_g G. \tag{S3}$$

Derivation of distribution (S3) is given in Supplementary Material Appendix B. Finally, given G, Z_1, Z_2 and D, we simulated X based on the linear model (7).

As shown in Supplementary Material Appendix B, the logistic and linear regression models in (7) hold simultaneously, and the relationship $\alpha_x = \beta_d$ and $\alpha_{gx} = \beta_{gd}$ is thus satisfied as well (we omit σ^2 since it was set as 1 in the simulation).

Supplementary Material Appendix E: Data generation procedures in Scenario I that $P(D|G, \boldsymbol{Z})$ follows a simple logistic regression model.

We considered two data generation procedures here.

- **Linear Procedure:** First, generate Z_1, Z_2, and G following the same procedure as described in Supplementary Material Appendix D. Then, generate the outcome, D, by

$$\text{logit}\Big(P(D = 1|G, Z_1, Z_2)\Big) = \alpha_0 + \alpha_1 Z_1 + \alpha_2 Z_1^2 + \alpha_3 Z_2 + \alpha_g G \tag{S4}$$

Finally, simulate the environmental exposure, X, based on the linear model in (7). The logistic regression model in (7) does not necessarily hold, because the $Pr(D|G, \boldsymbol{Z})$ should be (S3) if both the logistic and linear regression models hold, which is different with (S4) that we used to generate D.

- **Logistic Procedure:** The data generation process for Z_1, Z_2, and G was identical to the linear procedure. Then, the exposure, X, conditional on Z_1, Z_2, and G, was simulated by

$$X = E(X|Z_1, Z_2, G, D = 1)Pr(D = 1|Z_1, Z_2, G) + E(X|Z_1, Z_2, G, D = 0)Pr(D = 0|Z_1, Z_2, G) + \epsilon, \quad \text{(S5)}$$

where $\epsilon \sim N(0, 1)$, $E(X|Z_1, Z_2, G, D = 1)$ and $E(X|Z_1, Z_2, G, D = 0)$ refer to the conditional mean in the linear model (7), and $Pr(D = 1|Z_1, Z_2, G)$ is given by (S4). Finally, we generated D using the logistic model in (7). The linear model in (7) typically does not hold anymore. This is because $Pr(D = 1|Z_1, Z_2, G)$ and $Pr(D = 0|Z_1, Z_2, G)$ in (S5) are nonlinear functions of Z_1, Z_2 and G, but linear model in (7) only include the first and second order terms of these variables.

The parameter values in both the linear and logistic procedures are identical to those specified in section “Simulation study". We used the logistic regression test to evaluate $H_0 : \alpha_{gx} = 0$, and applied the reverse test to evaluate $H_0 : \beta_{gd} = 0$. For each simulation replicate, a full cohort including 100,000 subjects was generated, and a case-control study including all cases and an equal number of controls was randomly selected from the full cohort. Type I error rate was derived across 500,000 simulations for each model.

Supplementary Material Appendix F: Data generation procedures in Scenario II for the rectified gaussian distributed environmental exposure

The rectified gaussian distribution (Socci, Lee and Seung, 1998) resets the negative elements of a normal distribution to 0. It is a mixture of a continuous distribution (i.e., a truncated normal distribution with interval $(0, \infty)$) and a degenerate distribution (i.e., constant 0). The population density of a rectified gaussian distribution derived from $N(\mu, \sigma^2)$ is given by

$$f(x; \mu, \sigma^2) = \Phi(-\frac{\mu}{\sigma})\delta_x + \frac{1}{\sqrt{2\pi}\sigma} e^{-\frac{(x-\mu)^2}{2\sigma^2}} \mathbb{I}(x \geq 0),$$

where $\Phi(.)$ is the cumulative density function of standard normal distribution, $\mathbb{I}(.)$ is the indicator function, and δ_x is the Dirac delta function with $\delta_x = +\infty$ if $x = 0$ and $\delta_x = 0$ otherwise. In this simulation study, we let the error term in the linear model follow a rectified gaussian distribution instead of a normal distribution. To generate samples from the rectified gaussian distribution, we first simulated data from $N(3, 5^2)$ and then reset all the negative data to 0. In order to match the mean and variance to that of the standard normal distribution, we normalized the samples drawn from the rectified gaussian distribution by subtracting its mean and then dividing the difference by its empirical standard deviation. A density plot of the rectified gaussian distribution used in our simulation is given in Supplementary Material Figure S1.

Similar to Supplementary Material Appendix E, we considered two data generation procedures.

- **Linear Procedure:** First, generate Z_1, Z_2, and G following the same procedure as described in Supplementary Material Appendix D. Then generate the disease outcome, D, using the the logistic model (S3). Finally, simulate the environmental exposure, X, based on the linear model in (7), where the error term ϵ now follows a rectified gaussian distribution. Note that the logistic regression model in (7) generally does not hold since the conditional distribution $Pr(D|\boldsymbol{Z}, G)$ in (S3) was derived under the condition that the error term is normally distributed.

- **Logistic Procedure:** The data generation process for Z_1, Z_2, and G was identical with the linear procedure. Then, the exposure, X, conditional on Z_1, Z_2, and G, was simulated by

$$X = E(X|Z_1, Z_2, G, D = 1)Pr(D = 1|Z_1, Z_2, G) + E(X|Z_1, Z_2, G, D = 0)Pr(D = 0|Z_1, Z_2, G) + \tilde{\epsilon},$$

  where $\tilde{\epsilon}$ follows the rectified gaussian distribution, $E(X|Z_1, Z_2, G, D = 1)$ and $E(X|Z_1, Z_2, G, D = 0)$ refer to the conditional mean in linear model (7), and $Pr(D = 1|Z_1, Z_2, G)$ is given by (S3). Finally, we generated D using the logistic model in (7). Similar with what we discussed in Supplementary Material Appendix E, the linear model in (7) typically does not hold anymore.

Supplementary Material Appendix G: Time complexity for fitting the linear and logistic regression models.

First look at the linear regression model. The time complexity for the linear regression model is $O(np^2)$, where n is the sample size and p is the number of unknown coefficients (Drineas *et al.* 2010; Iyer, 2015).

Now, we consider the logistic regression model. There are several popular algorithms to fit logistic regression model, such as iteratively reweighted least squares (IRLS), gradient descent algorithm and Newton's method. Here, we only analyze the time complexity of IRLS in obtaining the coefficient estimators, which is also the default algorithm in R software to fit generalized linear models. We introduce the notations that will be used later. Let $\mathbf{\Phi}$ be a $n \times k$ design matrix for the logistic regression, where n is the sample size and k is the number of unknown coefficients. Denote $\boldsymbol{D} = [D_1, \ldots, D_n]^T$ as the response vector, which is a $n \times 1$ matrix. The IRLS is an iterative algorithm; on the t-th iteration, we have

$$\hat{\boldsymbol{\alpha}}^{(t)} = \hat{\boldsymbol{\alpha}}^{(t-1)} + (\mathbf{\Phi}^T \boldsymbol{R}^{(t)} \mathbf{\Phi})^{-1} \mathbf{\Phi}^T \boldsymbol{R}^{(t)} \boldsymbol{Z}^{(t)}, \tag{S6}$$

where $\boldsymbol{R}^{(t)} = diag\Big(\mu_1(1-\mu_1), \ldots, \mu_n(1-\mu_n)\Big)$ and $\boldsymbol{Z}^{(t)} = \mathbf{\Phi}\hat{\boldsymbol{\alpha}}^{(t-1)} - {\boldsymbol{R}^{(t)}}^{-1}(\boldsymbol{\mu} - \boldsymbol{D})$. Here, $\boldsymbol{\mu} = [\mu_1, \ldots, \mu_n]$, where $\mu_i = \frac{\exp(\mathbf{\Phi}_i^T \hat{\boldsymbol{\alpha}}^{(t-1)})}{1+\exp(\mathbf{\Phi}_i^T \hat{\boldsymbol{\alpha}}^{(t-1)})}$ and $\mathbf{\Phi}_i$ is the i-th row of $\mathbf{\Phi}$. The iterations proceed until convergence. In fact, the t-th iteration is fitting a weighted linear regression, with response vector $\boldsymbol{Z}^{(t)}$, design matrix $\mathbf{\Phi}$ and weighted matrix $\boldsymbol{R}^{(t)}$. Therefore, the t-th iteration should have similar computational complexity with the ordinary linear regression, as both are solving a system of normal equations and have a closed form solution. Now, we calculate the time complexity of the t-th iteration to confirm it.

- 1, Obtaining $\boldsymbol{Z}^{(t)}$ has $O(nk)$ complexity. Specifically, $\mathbf{\Phi}\hat{\boldsymbol{\alpha}}^{(t-1)}$ takes $O(nk)$ time, the inverse of the diagonal matrix $\boldsymbol{R}^{(t)}$ takes $O(n)$ time, $\boldsymbol{\mu} - \boldsymbol{D}$ takes $O(n)$ time, multiplication of ${\boldsymbol{R}^{(t)}}^{-1}$ and $(\boldsymbol{\mu} - \boldsymbol{D})$ takes $O(n)$ time, and subtraction ${\boldsymbol{R}^{(t)}}^{-1}(\boldsymbol{\mu} - \boldsymbol{D})$ from $\mathbf{\Phi}\hat{\boldsymbol{\alpha}}^{(t-1)}$ takes $O(n)$ time. Therefore, the Big-O time complexity of this step is $O(nk + 4n) = O(nk)$.

- 2, Obtaining $(\mathbf{\Phi}^T \boldsymbol{R}^{(t)} \mathbf{\Phi})^{-1}$ has $O(nk^2 + k^3)$ complexity. Specifically, $\mathbf{\Phi}^T \boldsymbol{R}^{(t)}$ takes $O(nk)$ time, multiplication of $(\mathbf{\Phi}^T \boldsymbol{R}^{(t)}$ and $\mathbf{\Phi}$ takes $O(nk^2)$ time, and inverse of $\mathbf{\Phi}^T \boldsymbol{R}^{(t)} \mathbf{\Phi}$ takes $O(k^3)$ time. Therefore, the Big-O time complexity of this step $O(nk^2 + k^3 + nk) = O(nk^2 + k^3)$.

- 3, Calculating $\mathbf{\Phi}^T \boldsymbol{R}^{(t)} \boldsymbol{Z}^{(t)}$ has $O(nk)$ complexity, in which $\mathbf{\Phi}^T \boldsymbol{R}^{(t)}$ takes $O(nk)$ time and multiplication of $\mathbf{\Phi}^T \boldsymbol{R}^{(t)}$ and $\boldsymbol{Z}^{(t)}$ takes $O(nk)$ time.

- 4, Multiplication of $(\mathbf{\Phi}^T \boldsymbol{R}^{(t)} \mathbf{\Phi})^{-1}$ and $\mathbf{\Phi}^T \boldsymbol{R}^{(t)} \boldsymbol{Z}^{(t)}$ has $O(k^2)$ complexity.

Combining Steps 1–4, the Big-O time complexity for the t-th iteration is $O(nk^2 + k^3)$. Because $k \leq n$ in general, we have $O(nk^2 + k^3) = O(nk^2)$. It follows that the t-th iteration of the IRLS share same order of time complexity with the linear model, when both have same number of unknown coefficients and sample size.

References

[1] Drineas, P., Mahoney, M. W., Muthukrishnan, S., & Sarlós, T. (2011). Faster least squares approximation. Numerische Mathematik, 117(2), 219-249.

[2] Iyer, K. T. (2015). Computational complexity of data mining algorithms used in fraud detection.

[3] Reinert, D. F., & Allen, J. P. (2007). The alcohol use disorders identification test: an update of research findings. Alcoholism: Clinical and Experimental Research, 31(2), 185-199.

[4] Socci, N. D., Lee, D. D., & Seung, H. S. (1998). The rectified gaussian distribution. In Advances in Neural Information Processing Systems, 350-356.

Supplementary Material Tables and Figures

Table S1: Basic characteristics, African Americans in the Veterans Aging Cohort Study (VACS).

	HIV+ $n = 965$	HIV- $n = 519$	Total $n = 1484$
Age Group			
$<$40 yrs,%	10.9	9.4	10.4
40-44 yrs,%	17.3	17.3	17.3
45-49 yrs,%	27.5	26.4	27.1
50-54 yrs,%	24.1	23.7	24.0
55-59 yrs,%	13.2	11.8	12.7
$\geq$60 yrs,%	7.0	11.4	8.6
Gender, % male	96.8	90.1	94.5
Smoking			
Smoker, %	63.7	57.4	61.5
Cigarettes/day, median	3.8	3.8	3.8
Cigarettes/day, IQR	10.0	10.0	10.0
Alcohol intake			
Drinker, %	90.9	91.5	91.2
AUDIT* score, median	2.3	2.3	2.3
AUDIT* of 8+, %	3.0	3.0	3.0

* The Alcohol Use Disorders Identification Test (AUDIT) reflects the fundamental relationship between people and alcohol intake, see Reinert and Allen (2007) for more details.

Table S2: Type I error rates at 5% significant level of the reverse test and logistic regression test, in Scenario I that $P(D=1|G,\boldsymbol{Z})$ follows a simple logistic regression model and the logistic regression model and the linear model do not hold simultaneously.

$P_0(D=1)$	$\mathrm{OR}(D\|G,X=0)$	$\mathrm{Corr}_0(X,Z_2)$	$\mathrm{Corr}_0(X,G)$	$\mathrm{OR}(D\|,X,G=0)$=1.1				$\mathrm{OR}(D\|X,G=0)$=1.5			
				X		$X^*(\rho=0.25)$		X		$X^*(\rho=0.25)$	
				logistic	reverse	logistic	reverse	logistic	reverse	logistic	reverse
Linear data generation procedure											
0.05	1.1	0.01	0.01	0.050	0.050	0.050	0.050	0.050	0.050	0.050	0.050
			0.2	0.050	0.050	0.050	0.050	0.050	0.050	0.050	0.050
		0.2	0.01	0.050	0.050	0.050	0.050	0.050	0.050	0.050	0.050
			0.2	0.050	0.050	0.050	0.050	0.050	0.050	0.050	0.051
	1.5	0.01	0.01	0.050	0.050	0.049	0.049	0.050	0.050	0.050	0.050
			0.2	0.049	0.050	0.050	0.050	0.050	0.050	0.050	0.050
		0.2	0.01	**0.049**	0.050	0.050	0.050	0.050	0.050	0.050	0.050
			0.2	0.050	0.050	0.050	0.050	0.050	0.050	0.050	0.050
0.2	1.1	0.01	0.01	0.050	0.050	0.050	0.050	0.050	0.051	**0.051**	**0.051**
			0.2	**0.049**	0.049	0.050	0.050	0.050	0.050	0.050	0.050
		0.2	0.01	**0.051**	0.050	0.050	0.050	0.050	0.050	0.050	0.050
			0.2	0.050	0.050	0.050	0.050	0.050	0.050	0.050	0.050
	1.5	0.01	0.01	0.050	0.050	0.050	0.050	0.050	0.050	0.050	0.050
			0.2	0.050	0.050	**0.051**	0.050	0.050	0.050	0.049	0.049
		0.2	0.01	0.050	0.050	0.050	**0.049**	0.050	0.050	0.050	0.050
			0.2	0.050	0.050	0.050	0.050	0.050	0.050	0.050	0.050
0.5	1.1	0.01	0.01	0.050	0.050	0.050	0.050	0.050	0.050	0.050	0.050
			0.2	0.050	0.050	0.050	0.050	0.050	0.050	0.050	0.050
		0.2	0.01	**0.051**	0.050	0.050	0.050	**0.049**	0.050	0.050	0.050
			0.2	0.050	**0.049**	0.050	0.050	0.050	0.050	0.050	0.050
	1.5	0.01	0.01	0.050	0.050	0.050	0.050	0.050	0.050	0.050	0.050
			0.2	0.050	0.050	0.050	0.049	0.050	0.050	0.050	0.050
		0.2	0.01	0.050	0.050	0.050	0.050	0.050	0.050	0.050	0.050
			0.2	0.050	0.050	0.050	0.050	0.050	0.050	0.050	0.050
Logistic data generation procedure											
0.05	1.1	0.01	0.01	0.050	0.050	0.050	0.050	0.050	0.050	0.050	0.050
			0.2	0.050	0.050	0.049	0.050	0.049	0.050	0.050	0.050
		0.2	0.01	0.050	0.050	0.050	0.050	0.050	0.050	0.050	0.050
			0.2	0.050	0.050	0.050	0.050	0.050	0.050	0.050	0.050
	1.5	0.01	0.01	0.050	0.050	0.050	0.050	0.050	**0.049**	0.050	**0.049**
			0.2	0.050	0.050	0.051	0.050	0.050	0.050	0.050	0.050
		0.2	0.01	0.050	0.050	0.050	0.050	0.050	**0.049**	0.050	0.050
			0.2	0.050	0.050	0.050	0.051	0.050	**0.049**	0.050	**0.049**
0.2	1.1	0.01	0.01	0.050	0.050	0.050	0.050	0.050	0.050	0.050	0.050
			0.2	0.050	0.050	0.050	0.050	0.050	0.050	0.050	0.050
		0.2	0.01	0.050	0.050	**0.049**	**0.049**	0.050	0.050	0.050	0.050
			0.2	0.050	0.050	0.050	0.050	0.050	0.050	0.050	0.050
	1.5	0.01	0.01	0.050	0.050	0.050	0.050	0.050	0.049	0.050	0.050
			0.2	0.049	0.049	0.050	0.050	0.050	0.050	0.050	**0.051**
		0.2	0.01	0.050	0.050	0.049	0.050	**0.049**	**0.049**	0.050	0.050
			0.2	0.050	0.050	0.050	0.050	0.050	0.050	0.051	**0.051**
0.5	1.1	0.01	0.01	0.050	0.050	0.049	0.050	0.050	0.050	0.051	0.051
			0.2	0.050	0.050	0.050	0.050	0.050	0.050	0.050	0.050
		0.2	0.01	**0.051**	0.051	0.050	0.050	0.050	0.050	0.050	0.051
			0.2	0.050	0.050	0.050	0.050	0.050	0.050	0.050	0.050
	1.5	0.01	0.01	0.050	0.050	0.050	0.050	0.050	0.051	0.050	0.050
			0.2	0.050	0.050	0.050	0.050	0.050	0.050	0.050	**0.051**
		0.2	0.01	0.050	0.050	0.050	0.050	0.050	0.051	0.050	**0.051**
			0.2	0.050	**0.051**	0.050	0.050	0.051	**0.051**	0.050	0.050

NOTE: In this table, "logistic" and "reverse" represent the logistic regression test and reverse test respectively. $P_0(D=1)$, $\mathrm{Corr}_0(X,Z_2)$, and $\mathrm{Corr}_0(X,G)$ denote $Pr(D=1|Z_1=Z_2=G=D=0)$, $\mathrm{Corr}(X,Z_2|Z_1=G=D=0)$ and $\mathrm{Corr}(X,G|Z_1=Z_2=G=D=0)$ respectively. Here, $\rho=\frac{\mathrm{Var}(X)}{\mathrm{Var}(X^*)}$ denotes the magnitude of the measurement error, where X is the true exposure and X^* is the observed exposure measured with error. The empirical type I error rates were calculated across 500,000 simulations for each scenario, where the empirical type I error rates outside the 95% confidence boundary, i.e., $p\pm1.96\times\sqrt{\frac{p(1-p)}{B}}$, were highlighted in bold. Here, p denotes the significance level (5%) and B denotes number of replication (500,000).

Table S3: Type I error rates at 5% significant level of the reverse test and logistic regression test, in Scenario II that the error term follows a rectified gaussian distribution with mean 0, variance 1 and coefficient of skewness 0.91.

$P_0(D=1)$	$\mathrm{OR}(D\|G,X=0)$	$\mathrm{Corr}_0(X,Z_2)$	$\mathrm{Corr}_0(X,G)$	$\mathrm{OR}(D\|,X,G=0)$=1.1				$\mathrm{OR}(D\|X,G=0)$=1.5			
				X		$X^*(\rho=0.25)$		X		$X^*(\rho=0.25)$	
				logistic	reverse	logistic	reverse	logistic	reverse	logistic	reverse
Linear data generation procedure											
0.05	1.1	0.01	0.01	0.050	0.050	0.050	0.050	**0.054**	0.050	0.050	0.050
			0.2	**0.051**	0.050	0.050	0.050	**0.056**	0.050	0.050	0.050
		0.2	0.01	0.050	0.050	0.050	0.051	**0.054**	0.050	0.050	0.050
			0.2	0.050	0.050	0.050	0.050	**0.055**	**0.049**	0.050	0.050
	1.5	0.01	0.01	**0.051**	0.050	**0.049**	0.050	**0.062**	0.050	0.050	0.050
			0.2	**0.051**	0.050	0.050	0.050	**0.066**	0.050	**0.051**	0.050
		0.2	0.01	**0.051**	0.050	0.050	0.050	**0.062**	0.050	0.050	0.050
			0.2	**0.051**	0.050	0.050	0.050	**0.065**	0.050	0.050	0.050
0.2	1.1	0.01	0.01	0.050	0.050	0.050	0.050	**0.054**	0.050	**0.051**	0.050
			0.2	0.050	**0.049**	0.050	0.050	**0.056**	0.050	0.050	0.050
		0.2	0.01	**0.051**	0.050	0.050	0.050	**0.054**	0.050	0.050	0.050
			0.2	0.050	0.050	0.050	0.050	**0.055**	0.050	0.050	0.050
	1.5	0.01	0.01	**0.052**	0.050	0.050	0.050	**0.063**	0.050	0.050	0.050
			0.2	**0.051**	0.050	**0.051**	0.050	**0.065**	0.050	0.050	0.050
		0.2	0.01	**0.052**	0.050	0.050	**0.049**	**0.061**	0.050	0.050	0.050
			0.2	**0.051**	0.050	0.050	0.050	**0.065**	0.050	**0.051**	0.051
0.5	1.1	0.01	0.01	0.051	0.050	0.050	0.050	**0.054**	0.050	0.050	0.050
			0.2	**0.051**	0.050	0.050	0.051	**0.056**	0.050	0.050	0.050
		0.2	0.01	**0.051**	0.050	0.050	**0.051**	**0.053**	0.050	0.050	0.050
			0.2	0.050	**0.049**	0.050	0.050	**0.055**	0.050	0.050	0.050
	1.5	0.01	0.01	**0.052**	0.051	0.050	0.050	**0.062**	0.050	0.050	0.050
			0.2	**0.052**	0.050	0.050	0.050	**0.066**	0.050	**0.051**	0.050
		0.2	0.01	**0.052**	0.050	0.050	0.050	**0.061**	0.049	0.050	0.050
			0.2	**0.051**	0.050	0.050	0.050	**0.064**	0.050	**0.051**	0.050
Logistic data generation procedure											
0.05	1.1	0.01	0.01	**0.049**	0.050	0.050	0.050	0.051	**0.049**	0.050	0.050
			0.2	0.050	0.050	0.050	0.050	0.050	**0.048**	0.050	**0.049**
		0.2	0.01	0.050	0.050	0.050	0.050	0.050	**0.048**	0.050	0.049
			0.2	0.050	0.049	0.050	0.050	0.050	**0.048**	0.050	**0.049**
	1.5	0.01	0.01	0.050	**0.048**	0.050	0.050	0.050	**0.048**	0.050	**0.049**
			0.2	0.050	**0.049**	0.050	0.050	0.050	**0.049**	0.050	0.050
		0.2	0.01	0.050	**0.049**	0.050	0.050	0.050	**0.048**	0.050	0.050
			0.2	0.050	**0.049**	0.050	0.050	0.050	**0.048**	**0.051**	0.050
0.2	1.1	0.01	0.01	0.050	0.050	0.050	0.050	0.050	**0.049**	0.050	0.050
			0.2	0.050	**0.049**	0.050	0.049	0.050	**0.048**	0.050	0.049
		0.2	0.01	0.050	0.050	0.050	0.050	0.050	**0.048**	0.050	0.050
			0.2	0.050	0.050	0.050	0.050	0.050	**0.048**	0.050	0.049
	1.5	0.01	0.01	0.050	**0.049**	0.050	0.050	0.050	0.050	0.050	0.050
			0.2	0.050	**0.049**	0.050	0.050	0.050	0.050	**0.051**	0.050
		0.2	0.01	0.049	**0.048**	**0.049**	**0.049**	0.050	**0.049**	**0.051**	**0.051**
			0.2	0.050	**0.048**	0.050	0.049	0.050	**0.051**	**0.051**	0.051
0.5	1.1	0.01	0.01	**0.049**	**0.049**	0.050	0.050	0.050	**0.049**	0.050	0.050
			0.2	0.050	0.049	0.050	0.050	0.050	**0.048**	**0.049**	**0.049**
		0.2	0.01	0.050	**0.049**	0.050	0.050	0.050	**0.049**	**0.049**	0.050
			0.2	0.050	**0.049**	0.050	0.050	0.050	**0.048**	0.050	0.050
	1.5	0.01	0.01	0.050	**0.049**	0.050	0.050	0.050	**0.047**	**0.049**	**0.049**
			0.2	**0.049**	**0.048**	0.049	**0.049**	0.050	**0.047**	0.050	**0.049**
		0.2	0.01	0.050	**0.048**	0.050	0.050	0.050	**0.047**	0.050	0.050
			0.2	0.050	**0.049**	0.050	0.050	0.050	**0.047**	0.050	**0.049**

NOTE: In this table, "logistic" and "reverse" represent the logistic regression test and reverse test respectively. $P_0(D=1)$, $\mathrm{Corr}_0(X,Z_2)$, and $\mathrm{Corr}_0(X,G)$ denote $Pr(D=1|Z_1=Z_2=G=D=0)$, $\mathrm{Corr}(X,Z_2|Z_1=G=D=0)$ and $\mathrm{Corr}(X,G|Z_1=Z_2=G=D=0)$ respectively. Here, $\rho=\frac{\mathrm{Var}(X)}{\mathrm{Var}(X^*)}$ denotes the magnitude of the measurement error, where X is the true exposure and X^* is the observed exposure measured with error. The empirical type I error rates were calculated across 500,000 simulations for each scenario, where the empirical type I error rates outside the 95% confidence boundary, i.e., $p \pm 1.96 \times \sqrt{\frac{p(1-p)}{B}}$, were highlighted in bold. Here, p denotes the significance level (5%) and B denotes number of replication (500,000).

Table S4: Type I error rates at 0.01% significant level of the reverse test and logistic regression test, in Scenario II that the error term follows a rectified gaussian distribution with mean 0, variance 1 and coefficient of skewness 0.91.

$P_0(D=1)$	OR$(D\|G,X=0)$	Corr$_0(X,Z_2)$	Corr$_0(X,G)$	OR$(D\|,X,G=0)$=1.1				OR$(D\|X,G=0)$=1.5			
				X		$X^*(\rho=0.25)$		X		$X^*(\rho=0.25)$	
				logistic	reverse	logistic	reverse	logistic	reverse	logistic	reverse
Linear data generation procedure											
0.05	1.1	0.01	0.01	7.8e-05	8.2e-05	7.4e-05	9.8e-05	9.2e-05	9.0e-05	9.2e-05	**1.3e-04**
			0.2	9.2e-05	1.1e-04	9.2e-05	1.1e-04	**1.5e-04**	1.1e-04	9.4e-05	9.2e-05
		0.2	0.01	**6.8e-05**	1.2e-04	8.2e-05	1.2e-04	1.1e-04	8.2e-05	9.6e-05	1.0e-04
			0.2	8.2e-05	1.1e-04	**6.8e-05**	1.1e-04	**1.7e-04**	1.2e-04	**7.0e-05**	8.2e-05
	1.5	0.01	0.01	7.8e-05	8.0e-05	7.6e-05	8.6e-05	**1.7e-04**	1.0e-04	8.8e-05	1.0e-04
			0.2	**7.0e-05**	8.0e-05	**6.8e-05**	1.3e-04	**1.9e-04**	1.2e-04	1.2e-04	1.1e-04
		0.2	0.01	**7.2e-05**	9.2e-05	**6.2e-05**	8.4e-05	**1.5e-04**	9.8e-05	8.6e-05	8.4e-05
			0.2	1.1e-04	1.1e-04	9.4e-05	1.0e-04	**1.8e-04**	1.1e-04	**6.2e-05**	7.8e-05
0.2	1.1	0.01	0.01	8.0e-05	8.4e-05	7.6e-05	1.0e-04	1.2e-04	1.1e-04	7.6e-05	9.0e-05
			0.2	9.0e-05	1.0e-04	8.4e-05	8.6e-05	1.1e-04	1.2e-04	1.0e-04	1.1e-04
		0.2	0.01	9.4e-05	9.8e-05	1.2e-04	1.2e-04	9.4e-05	8.8e-05	9.8e-05	1.2e-04
			0.2	**6.4e-05**	9.4e-05	**7.2e-05**	9.6e-05	1.2e-04	7.8e-05	8.4e-05	8.4e-05
	1.5	0.01	0.01	9.0e-05	1.2e-04	9.6e-05	1.3e-04	**1.5e-04**	9.6e-05	1.2e-04	**1.3e-04**
			0.2	1.0e-04	1.1e-04	8.6e-05	1.0e-04	**1.6e-04**	1.2e-04	**7.0e-05**	1.1e-04
		0.2	0.01	1.1e-04	1.0e-04	7.4e-05	1.0e-04	**1.7e-04**	9.0e-05	9.2e-05	1.0e-04
			0.2	**7.2e-05**	9.6e-05	**6.2e-05**	**7.0e-05**	**1.6e-04**	1.1e-04	9.8e-05	1.1e-04
0.5	1.1	0.01	0.01	7.6e-05	7.6e-05	**5.4e-05**	7.6e-05	8.6e-05	8.4e-05	9.4e-05	9.6e-05
			0.2	1.0e-04	1.0e-04	**7.2e-05**	9.0e-05	1.0e-04	1.0e-04	8.8e-05	1.0e-04
		0.2	0.01	8.0e-05	9.0e-05	7.8e-05	7.6e-05	1.2e-04	9.8e-05	7.8e-05	9.4e-05
			0.2	7.6e-05	1.1e-04	8.8e-05	9.6e-05	1.1e-04	1.2e-04	**5.6e-05**	7.4e-05
	1.5	0.01	0.01	9.8e-05	9.4e-05	7.8e-05	8.4e-05	**1.6e-04**	9.4e-05	8.6e-05	9.6e-05
			0.2	1.2e-04	9.0e-05	**7.2e-05**	9.8e-05	**1.4e-04**	9.6e-05	1.1e-04	1.0e-04
		0.2	0.01	8.2e-05	9.4e-05	9.0e-05	1.1e-04	**1.6e-04**	1.1e-04	8.6e-05	9.6e-05
			0.2	8.2e-05	1.1e-04	**6.0e-05**	8.6e-05	**1.5e-04**	8.8e-05	1.1e-04	1.2e-04
Logistic data generation procedure											
0.05	1.1	0.01	0.01	7.8e-05	9.0e-05	1.1e-04	1.2e-04	7.8e-05	8.8e-05	7.4e-05	8.2e-05
			0.2	8.6e-05	1.1e-04	8.8e-05	8.8e-05	8.8e-05	9.2e-05	8.6e-05	1.0e-04
		0.2	0.01	8.4e-05	1.0e-04	1.1e-04	1.2e-04	1.0e-04	8.6e-05	1.0e-04	1.1e-04
			0.2	8.8e-05	9.4e-05	**5.4e-05**	8.4e-05	8.4e-05	**6.4e-05**	1.2e-04	1.0e-04
	1.5	0.01	0.01	1.1e-04	1.0e-04	9.6e-05	1.1e-04	1.0e-04	9.8e-05	9.0e-05	7.8e-05
			0.2	9.2e-05	8.8e-05	9.6e-05	8.6e-05	8.4e-05	9.8e-05	8.4e-05	9.8e-05
		0.2	0.01	8.6e-05	9.2e-05	9.8e-05	1.1e-04	8.2e-05	1.0e-04	1.1e-04	9.4e-05
			0.2	9.2e-05	**6.2e-05**	9.4e-05	1.1e-04	**6.4e-05**	1.0e-04	9.6e-05	1.0e-04
0.2	1.1	0.01	0.01	9.6e-05	8.4e-05	9.2e-05	1.2e-04	7.6e-05	7.4e-05	9.6e-05	1.0e-04
			0.2	**7.2e-05**	8.6e-05	9.2e-05	**1.3e-04**	7.8e-05	8.8e-05	7.4e-05	8.4e-05
		0.2	0.01	7.8e-05	1.0e-04	1.1e-04	1.2e-04	9.0e-05	8.6e-05	8.4e-05	9.4e-05
			0.2	**7.2e-05**	9.8e-05	9.4e-05	1.1e-04	1.0e-04	7.6e-05	1.1e-04	8.6e-05
	1.5	0.01	0.01	1.0e-04	9.2e-05	7.4e-05	9.6e-05	1.1e-04	9.4e-05	9.6e-05	1.2e-04
			0.2	8.4e-05	**7.2e-05**	9.8e-05	1.2e-04	8.6e-05	8.8e-05	1.2e-04	1.1e-04
		0.2	0.01	**6.2e-05**	**7.0e-05**	8.4e-05	9.6e-05	8.2e-05	7.8e-05	8.0e-05	1.2e-04
			0.2	7.8e-05	**7.0e-05**	7.8e-05	7.8e-05	1.0e-04	1.1e-04	9.6e-05	1.0e-04
0.5	1.1	0.01	0.01	7.8e-05	1.0e-04	**6.4e-05**	8.6e-05	**7.2e-05**	9.0e-05	9.0e-05	1.0e-04
			0.2	8.0e-05	9.6e-05	9.8e-05	1.1e-04	1.1e-04	9.2e-05	**5.6e-05**	**6.6e-05**
		0.2	0.01	**7.0e-05**	8.8e-05	**6.4e-05**	9.2e-05	8.2e-05	1.1e-04	1.2e-04	1.2e-04
			0.2	**6.8e-05**	9.0e-05	9.6e-05	**1.3e-04**	1.1e-04	1.1e-04	9.6e-05	9.4e-05
	1.5	0.01	0.01	7.6e-05	8.4e-05	**7.2e-05**	8.8e-05	**6.8e-05**	8.0e-05	**6.0e-05**	8.0e-05
			0.2	1.1e-04	1.2e-04	9.0e-05	9.0e-05	9.0e-05	**6.8e-05**	7.4e-05	8.4e-05
		0.2	0.01	**5.8e-05**	7.8e-05	8.2e-05	9.6e-05	7.4e-05	7.6e-05	9.0e-05	1.0e-04
			0.2	8.0e-05	9.0e-05	**7.0e-05**	8.8e-05	8.0e-05	**5.8e-05**	8.6e-05	9.8e-05

NOTE: In this table, "logistic" and "reverse" represent the logistic regression test and reverse test respectively. $P_0(D=1)$, Corr$_0(X,Z_2)$, and Corr$_0(X,G)$ denote $Pr(D=1|Z_1=Z_2=G=D=0)$, Corr$(X,Z_2|Z_1=G=D=0)$ and Corr$(X,G|Z_1=Z_2=G=D=0)$ respectively. Here, $\rho=\frac{\mathrm{Var}(X)}{\mathrm{Var}(X^*)}$ denotes the magnitude of the measurement error, where X is the true exposure and X^* is the observed exposure measured with error. The empirical type I error rates were calculated across 500,000 simulations for each scenario, where the empirical type I error rates outside the 95% confidence boundary, i.e., $p\pm 1.96\times\sqrt{\frac{p(1-p)}{B}}$, were highlighted in bold. Here, p denotes the significance level (0.01%) and B denotes number of replication (500,000).

Table S5: Rejection rates under significance levels at 0.01% and 5%, when the logistic regression model and the linear model hold simultaneously. Here, ROR was specified as 1.5.

$P_0(D=1)$	$\text{OR}(D\|G,X=0)$	$\text{Corr}_0(X,Z_2)$	$\text{Corr}_0(X,G)$	$\text{OR}(D\|,X,G=0)$=1.1				$\text{OR}(D\|X,G=0)$=1.5			
				X		$X^*(\rho=0.25)$		X		$X^*(\rho=0.25)$	
				logistic	reverse	logistic	reverse	logistic	reverse	logistic	reverse
0.01% significance level											
0.05	1.1	0.01	0.01	96.1	**98.6**	16.4	**20.3**	91.1	**98.8**	14.3	**20.4**
			0.2	97.8	**98.8**	**21.8**	21.5	93.1	**98.8**	17.6	**21.2**
		0.2	0.01	97.2	**98.7**	19.2	**20.6**	92.1	**98.8**	16.1	**20.7**
			0.2	98.3	**98.9**	**24.3**	21.2	93.3	**98.7**	18.6	**20.6**
	1.5	0.01	0.01	96.3	**98.8**	16.2	**20.9**	90.5	**98.7**	13.8	**20.6**
			0.2	97.1	**98.8**	19.5	**21.0**	89.7	**98.3**	14.1	**19.0**
		0.2	0.01	97.1	**98.9**	18.5	**21.0**	90.9	**98.7**	15.0	**20.2**
			0.2	97.3	**98.7**	**21.7**	20.6	89.3	**98.0**	15.0	**18.3**
0.2	1.1	0.01	0.01	94.9	**97.8**	14.1	**17.4**	87.7	**97.7**	11.6	**17.2**
			0.2	95.8	**97.5**	**16.7**	16.7	86.0	**96.4**	12.0	**15.0**
		0.2	0.01	95.9	**97.8**	16.3	**17.4**	87.8	**97.4**	12.6	**16.7**
			0.2	96.2	**97.3**	**18.6**	16.5	86.0	**95.9**	12.4	**14.3**
	1.5	0.01	0.01	93.3	**97.2**	12.7	**16.5**	83.1	**96.6**	10.0	**15.4**
			0.2	92.6	**96.2**	13.4	**14.7**	77.9	**93.8**	8.5	**12.2**
		0.2	0.01	93.9	**97.1**	13.8	**15.7**	83.4	**95.9**	10.0	**13.9**
			0.2	92.9	**95.8**	14.0	**14.0**	76.6	**92.7**	8.6	**11.3**
0.5	1.1	0.01	0.01	92.3	**96.2**	11.6	**14.4**	80.6	**95.1**	9.0	**13.5**
			0.2	91.4	**94.3**	11.8	**12.1**	73.9	**90.7**	7.5	**10.0**
		0.2	0.01	92.8	**95.8**	12.8	**14.0**	80.7	**94.4**	9.4	**12.7**
			0.2	91.3	**93.7**	**12.9**	11.9	73.3	**89.5**	7.6	**9.4**
	1.5	0.01	0.01	86.9	**93.5**	8.7	**11.5**	71.4	**91.0**	6.4	**10.5**
			0.2	82.9	**89.6**	8.0	**9.3**	60.9	**83.6**	4.7	**7.2**
		0.2	0.01	87.3	**92.6**	9.3	**11.2**	71.1	**89.9**	6.7	**9.7**
			0.2	83.2	**88.5**	8.7	**9.1**	59.0	**81.8**	4.8	**6.9**
5% significance level											
0.05	1.1	0.01	0.01	100.0	**100.0**	84.8	**86.1**	100.0	**100.0**	82.9	**86.4**
			0.2	100.0	**100.0**	**89.1**	87.1	100.0	**100.0**	86.6	**87.1**
		0.2	0.01	100.0	**100.0**	**87.3**	86.6	100.0	**100.0**	85.2	**86.8**
			0.2	**100.0**	100.0	**90.8**	87.2	100.0	**100.0**	**87.8**	86.7
	1.5	0.01	0.01	100.0	**100.0**	84.7	**87.1**	100.0	**100.0**	82.2	**86.7**
			0.2	100.0	**100.0**	**87.8**	87.0	100.0	**100.0**	83.5	**85.6**
		0.2	0.01	100.0	**100.0**	86.6	**86.7**	100.0	**100.0**	83.5	**86.3**
			0.2	100.0	**100.0**	**89.3**	86.6	100.0	**100.0**	83.8	**84.5**
0.2	1.1	0.01	0.01	100.0	**100.0**	82.6	**84.1**	99.9	**100.0**	80.4	**83.6**
			0.2	**100.0**	100.0	**85.9**	83.5	99.9	**100.0**	81.1	**81.2**
		0.2	0.01	100.0	**100.0**	**84.8**	83.9	99.9	**100.0**	81.5	**82.9**
			0.2	100.0	**100.0**	**87.5**	83.0	99.9	**100.0**	**81.5**	80.0
	1.5	0.01	0.01	100.0	**100.0**	80.6	**82.9**	99.9	**100.0**	76.7	**81.4**
			0.2	100.0	**100.0**	**81.9**	80.4	99.8	**100.0**	75.6	**77.7**
		0.2	0.01	100.0	**100.0**	**82.7**	82.6	99.9	**100.0**	77.7	**80.4**
			0.2	100.0	**100.0**	**83.6**	80.2	99.8	**100.0**	76.0	**76.6**
0.5	1.1	0.01	0.01	100.0	**100.0**	79.8	**80.9**	99.9	**100.0**	76.3	**79.5**
			0.2	100.0	**100.0**	**80.8**	78.2	99.8	**99.9**	74.1	**74.6**
		0.2	0.01	100.0	**100.0**	**81.7**	80.4	99.9	**100.0**	77.2	**78.4**
			0.2	**100.0**	100.0	**82.6**	77.6	99.7	**99.9**	**74.5**	73.0
	1.5	0.01	0.01	99.9	**100.0**	75.1	**77.6**	99.6	**99.9**	70.4	**74.7**
			0.2	99.9	**99.9**	**74.4**	73.2	99.3	**99.8**	65.9	**68.4**
		0.2	0.01	100.0	**100.0**	**76.3**	76.1	99.7	**99.9**	71.0	**73.6**
			0.2	99.9	**99.9**	**76.1**	72.4	99.2	**99.8**	66.2	**66.7**

NOTE: In this table, "logistic" and "reverse" represent the logistic regression test and reverse test respectively. $P_0(D=1)$, $\text{Corr}_0(X,Z_2)$, and $\text{Corr}_0(X,G)$ denote $Pr(D=1|Z_1=Z_2=G=D=0)$, $\text{Corr}(X,Z_2|Z_1=G=D=0)$ and $\text{Corr}(X,G|Z_1=Z_2=G=D=0)$ respectively. Here, $\rho=\frac{\text{Var}(X)}{\text{Var}(X^*)}$ denotes the magnitude of the measurement error, where X is the true exposure and X^* is the observed exposure measured with error. The rejection rates were calculate the average rejections across 50,000 simulations for each scenario. The higher rejection rate between the two tests was highlighted in bold.

Table S6: Relative power of the reverse test against logistic regression test, in Scenario I that $P(D=1|G,\boldsymbol{Z})$ follows a simple logistic regression model and the logistic regression model and the linear model do not hold simultaneously. Here, ROR was specified as 1.5.

$P_0(D=1)$	$\mathrm{OR}(D\|G,X=0)$	$\mathrm{Corr}_0(X,Z_2)$	$\mathrm{Corr}_0(X,G)$	χ^2 ratio							
				$\mathrm{OR}(D\|X,G=0)$=1.1				$\mathrm{OR}(D\|X,G=0)$=1.5			
				$\rho=1$	$\rho=0.75$	$\rho=0.5$	$\rho=0.25$	$\rho=1$	$\rho=0.75$	$\rho=0.5$	$\rho=0.25$
Linear data generation procedure											
0.05	1.1	0.01	0.01	1.25	1.20	1.14	1.08	1.48	1.36	1.25	1.14
			0.2	1.21	1.13	1.06	0.99	1.43	1.30	1.18	1.07
		0.2	0.01	1.22	1.15	1.08	1.02	1.45	1.32	1.20	1.09
			0.2	1.18	1.09	1.01	0.94	1.41	1.27	1.14	1.02
	1.5	0.01	0.01	1.27	1.21	1.16	1.10	1.49	1.38	1.27	1.16
			0.2	1.23	1.16	1.09	1.02	1.46	1.34	1.22	1.11
		0.2	0.01	1.24	1.17	1.10	1.04	1.46	1.34	1.23	1.12
			0.2	1.21	1.12	1.04	0.97	1.44	1.31	1.18	1.07
0.2	1.1	0.01	0.01	1.24	1.19	1.13	1.07	1.46	1.35	1.24	1.13
			0.2	1.19	1.12	1.05	0.99	1.42	1.29	1.17	1.06
		0.2	0.01	1.21	1.14	1.08	1.02	1.44	1.31	1.20	1.08
			0.2	1.17	1.09	1.01	0.94	1.39	1.26	1.14	1.02
	1.5	0.01	0.01	1.25	1.20	1.15	1.09	1.47	1.36	1.25	1.15
			0.2	1.22	1.15	1.08	1.02	1.44	1.32	1.20	1.10
		0.2	0.01	1.22	1.16	1.10	1.04	1.44	1.32	1.21	1.11
			0.2	1.19	1.12	1.04	0.97	1.42	1.29	1.17	1.06
0.5	1.1	0.01	0.01	1.23	1.18	1.12	1.07	1.45	1.34	1.23	1.12
			0.2	1.19	1.12	1.05	0.99	1.41	1.28	1.17	1.06
		0.2	0.01	1.20	1.14	1.07	1.02	1.43	1.31	1.19	1.08
			0.2	1.16	1.08	1.01	0.94	1.38	1.26	1.14	1.03
	1.5	0.01	0.01	1.24	1.19	1.14	1.08	1.45	1.34	1.24	1.14
			0.2	1.21	1.14	1.08	1.02	1.42	1.30	1.19	1.09
		0.2	0.01	1.21	1.15	1.09	1.03	1.42	1.31	1.20	1.10
			0.2	1.18	1.11	1.04	0.98	1.40	1.27	1.16	1.06
Logistic data generation procedure											
0.05	1.1	0.01	0.01	1.12	1.10	1.07	1.04	1.05	1.04	1.04	1.03
			0.2	1.04	1.01	0.97	0.94	0.94	0.93	0.92	0.93
		0.2	0.01	1.08	1.04	1.01	0.98	1.01	0.99	0.98	0.97
			0.2	1.01	0.96	0.92	0.89	0.92	0.90	0.88	0.88
	1.5	0.01	0.01	1.10	1.08	1.06	1.05	0.99	1.00	1.02	1.04
			0.2	1.04	1.01	0.98	0.96	0.95	0.95	0.95	0.97
		0.2	0.01	1.06	1.03	1.01	0.98	0.96	0.96	0.97	0.98
			0.2	1.01	0.97	0.93	0.91	0.95	0.94	0.93	0.94
0.2	1.1	0.01	0.01	1.05	1.05	1.04	1.03	0.95	0.97	1.00	1.02
			0.2	1.01	0.98	0.96	0.93	0.96	0.95	0.94	0.95
		0.2	0.01	1.02	1.00	0.98	0.97	0.95	0.95	0.96	0.97
			0.2	0.99	0.95	0.91	0.88	0.97	0.94	0.92	0.92
	1.5	0.01	0.01	1.07	1.06	1.06	1.05	0.99	1.01	1.04	1.06
			0.2	1.06	1.03	1.00	0.97	1.05	1.04	1.02	1.02
		0.2	0.01	1.05	1.03	1.01	0.99	0.99	1.00	1.01	1.02
			0.2	1.04	1.00	0.96	0.93	1.05	1.03	1.00	0.98
0.5	1.1	0.01	0.01	1.06	1.05	1.04	1.03	1.03	1.04	1.04	1.03
			0.2	1.05	1.01	0.98	0.95	1.09	1.05	1.01	0.98
		0.2	0.01	1.04	1.02	0.99	0.97	1.04	1.02	1.00	0.99
			0.2	1.04	0.99	0.94	0.90	1.09	1.04	0.99	0.95
	1.5	0.01	0.01	1.11	1.09	1.08	1.06	1.12	1.11	1.10	1.08
			0.2	1.11	1.07	1.03	0.99	1.17	1.13	1.08	1.04
		0.2	0.01	1.09	1.06	1.03	1.01	1.12	1.09	1.06	1.03
			0.2	1.09	1.04	0.99	0.95	1.16	1.10	1.05	1.00

NOTE: $P_0(D=1)$, $\mathrm{Corr}_0(X,Z_2)$, and $\mathrm{Corr}_0(X,G)$ denote $Pr(D=1|Z_1=Z_2=G=D=0)$, $\mathrm{Corr}(X,Z_2|Z_1=G=D=0)$ and $\mathrm{Corr}(X,G|Z_1=Z_2=G=D=0)$ respectively. Here, $\rho=\frac{\mathrm{Var}(X)}{\mathrm{Var}(X^*)}$ denotes the magnitude of the measurement error, where X is the true exposure and X^* is the observed exposure measured with error. The relative power was calculated by $\frac{\bar{\chi}^2_{\mathrm{rev}}}{\bar{\chi}^2_{\mathrm{log}}}$ where $\bar{\chi}^2_{\mathrm{rev}}$ and $\bar{\chi}^2_{\mathrm{rev}}$ are the average χ^2 statistics for the logistic regression test and reverse test based on 500,000 simulation repetitions for each scenario.

Table S7: Relative power of the reverse test against logistic regression test, in Scenario II that the error term follows a rectified gaussian distribution with mean 0, variance 1 and coefficient of skewness 0.91. Here, ROR was specified as 1.5.

$P_0(D=1)$	$\mathrm{OR}(D\|G,X=0)$	$\mathrm{Corr}_0(X,Z_2)$	$\mathrm{Corr}_0(X,G)$	χ^2 ratio							
				$\mathrm{OR}(D\|X,G=0)$=1.1				$\mathrm{OR}(D\|X,G=0)$=1.5			
				$\rho=1$	$\rho=0.75$	$\rho=0.5$	$\rho=0.25$	$\rho=1$	$\rho=0.75$	$\rho=0.5$	$\rho=0.25$
Linear data generation procedure											
0.05	1.1	0.01	0.01	1.19	1.16	1.12	1.08	1.23	1.22	1.19	1.12
			0.2	1.11	1.08	1.03	0.98	1.08	1.10	1.09	1.04
		0.2	0.01	1.15	1.11	1.07	1.02	1.19	1.17	1.14	1.07
			0.2	1.08	1.04	0.99	0.93	1.06	1.07	1.05	1.00
	1.5	0.01	0.01	1.14	1.14	1.13	1.09	1.11	1.15	1.17	1.14
			0.2	1.09	1.08	1.05	1.01	1.01	1.07	1.09	1.08
		0.2	0.01	1.11	1.10	1.07	1.03	1.09	1.12	1.12	1.09
			0.2	1.06	1.04	1.01	0.96	1.00	1.05	1.06	1.04
0.2	1.1	0.01	0.01	1.18	1.15	1.11	1.07	1.23	1.21	1.18	1.11
			0.2	1.11	1.07	1.03	0.98	1.09	1.10	1.09	1.04
		0.2	0.01	1.14	1.10	1.06	1.01	1.19	1.17	1.13	1.07
			0.2	1.08	1.04	0.98	0.93	1.07	1.08	1.05	1.00
	1.5	0.01	0.01	1.14	1.14	1.11	1.08	1.11	1.15	1.16	1.13
			0.2	1.09	1.08	1.04	1.00	1.02	1.08	1.09	1.07
		0.2	0.01	1.11	1.10	1.07	1.03	1.09	1.12	1.12	1.08
			0.2	1.07	1.05	1.01	0.96	1.02	1.05	1.06	1.03
0.5	1.1	0.01	0.01	1.17	1.14	1.10	1.06	1.22	1.21	1.17	1.11
			0.2	1.10	1.07	1.02	0.98	1.10	1.11	1.09	1.04
		0.2	0.01	1.14	1.10	1.05	1.01	1.19	1.18	1.13	1.07
			0.2	1.08	1.04	0.99	0.93	1.08	1.08	1.06	1.01
	1.5	0.01	0.01	1.14	1.13	1.11	1.07	1.11	1.15	1.15	1.12
			0.2	1.10	1.08	1.05	1.01	1.03	1.08	1.09	1.07
		0.2	0.01	1.11	1.09	1.06	1.03	1.09	1.12	1.12	1.08
			0.2	1.07	1.05	1.01	0.97	1.02	1.06	1.06	1.03
Logistic data generation procedure											
0.05	1.1	0.01	0.01	1.36	1.28	1.19	1.09	1.21	1.16	1.11	1.06
			0.2	1.23	1.16	1.08	0.99	0.96	0.96	0.96	0.95
		0.2	0.01	1.30	1.21	1.13	1.04	1.12	1.08	1.05	1.00
			0.2	1.17	1.10	1.02	0.94	0.89	0.90	0.90	0.89
	1.5	0.01	0.01	1.26	1.21	1.16	1.09	1.01	1.03	1.04	1.04
			0.2	1.14	1.10	1.05	0.99	0.82	0.87	0.92	0.95
		0.2	0.01	1.20	1.15	1.09	1.02	0.94	0.96	0.98	0.98
			0.2	1.09	1.04	0.99	0.93	0.78	0.83	0.87	0.90
0.2	1.1	0.01	0.01	1.12	1.11	1.08	1.05	0.90	0.95	1.00	1.03
			0.2	1.03	1.01	0.98	0.95	0.76	0.83	0.89	0.93
		0.2	0.01	1.07	1.05	1.02	0.99	0.85	0.90	0.94	0.97
			0.2	0.99	0.96	0.93	0.89	0.74	0.80	0.85	0.88
	1.5	0.01	0.01	1.07	1.08	1.07	1.06	0.79	0.90	1.00	1.06
			0.2	1.01	1.01	0.99	0.97	0.73	0.83	0.92	0.98
		0.2	0.01	1.02	1.03	1.02	1.00	0.76	0.86	0.94	1.00
			0.2	0.98	0.97	0.94	0.92	0.71	0.81	0.88	0.94
0.5	1.1	0.01	0.01	0.97	0.99	1.01	1.02	0.75	0.86	0.95	1.01
			0.2	0.92	0.93	0.92	0.92	0.69	0.79	0.86	0.91
		0.2	0.01	0.94	0.95	0.95	0.95	0.73	0.83	0.90	0.95
			0.2	0.90	0.89	0.88	0.87	0.68	0.76	0.82	0.86
	1.5	0.01	0.01	0.97	1.01	1.03	1.05	0.73	0.88	0.99	1.06
			0.2	0.95	0.96	0.96	0.96	0.69	0.81	0.91	0.96
		0.2	0.01	0.94	0.96	0.98	0.98	0.71	0.83	0.93	0.99
			0.2	0.92	0.92	0.92	0.91	0.67	0.78	0.86	0.91

NOTE: $P_0(D=1)$, $\mathrm{Corr}_0(X,Z_2)$, and $\mathrm{Corr}_0(X,G)$ denote $Pr(D=1|Z_1=Z_2=G=D=0)$, $\mathrm{Corr}(X,Z_2|Z_1=G=D=0)$ and $\mathrm{Corr}(X,G|Z_1=Z_2=G=D=0)$ respectively. Here, $\rho=\frac{\mathrm{Var}(X)}{\mathrm{Var}(X^*)}$ denotes the magnitude of the measurement error, where X is the true exposure and X^* is the observed exposure measured with error. The relative power was calculated by $\frac{\bar{\chi}^2_{\mathrm{rev}}}{\bar{\chi}^2_{\mathrm{log}}}$ where $\bar{\chi}^2_{\mathrm{rev}}$ and $\bar{\chi}^2_{\mathrm{rev}}$ are the average χ^2 statistics for the logistic regression test and reverse test based on 500,000 simulation repetitions for each scenario.

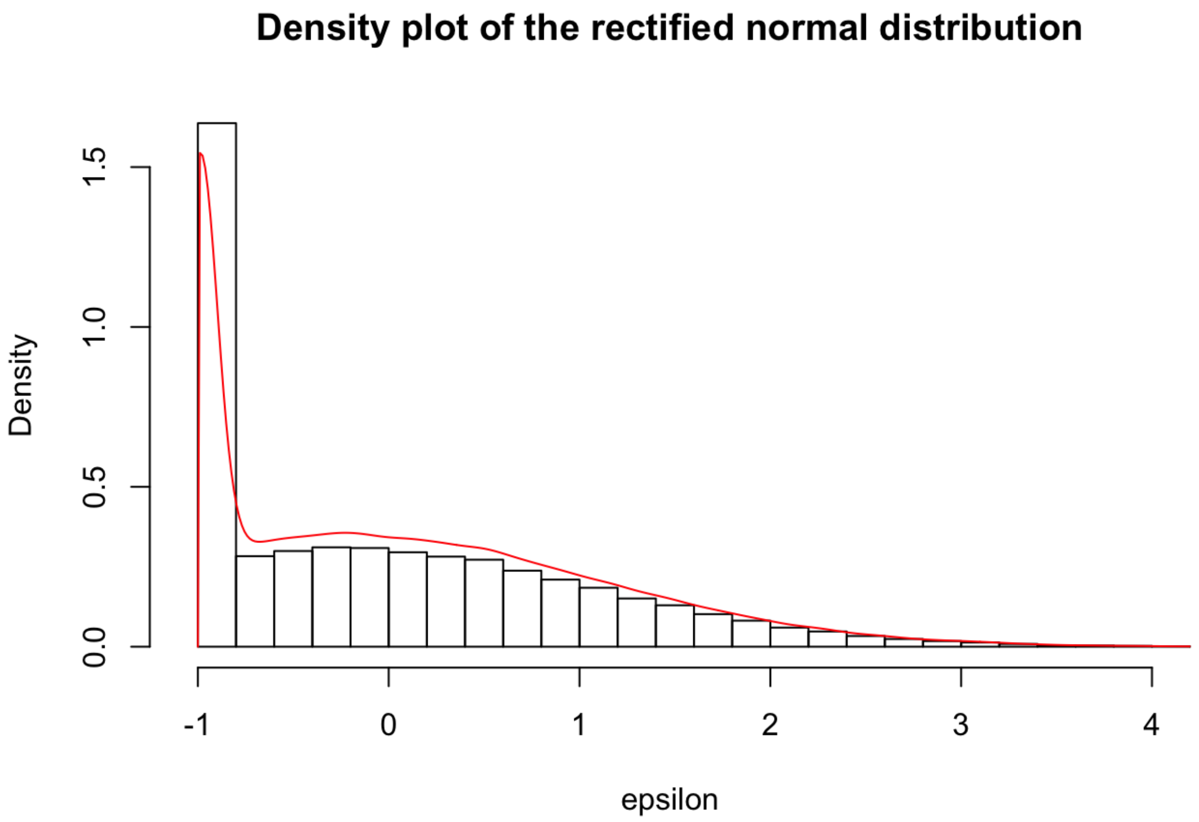

Figure S1: Density plot of the rectified gaussian distribution used in the simulation to generate ϵ in the linear regression model, where a histogram of 500,000 samples from this distribution was also added in this plot. The coefficients of skewness and kurtosis were 0.91 and 3.25, respectively.

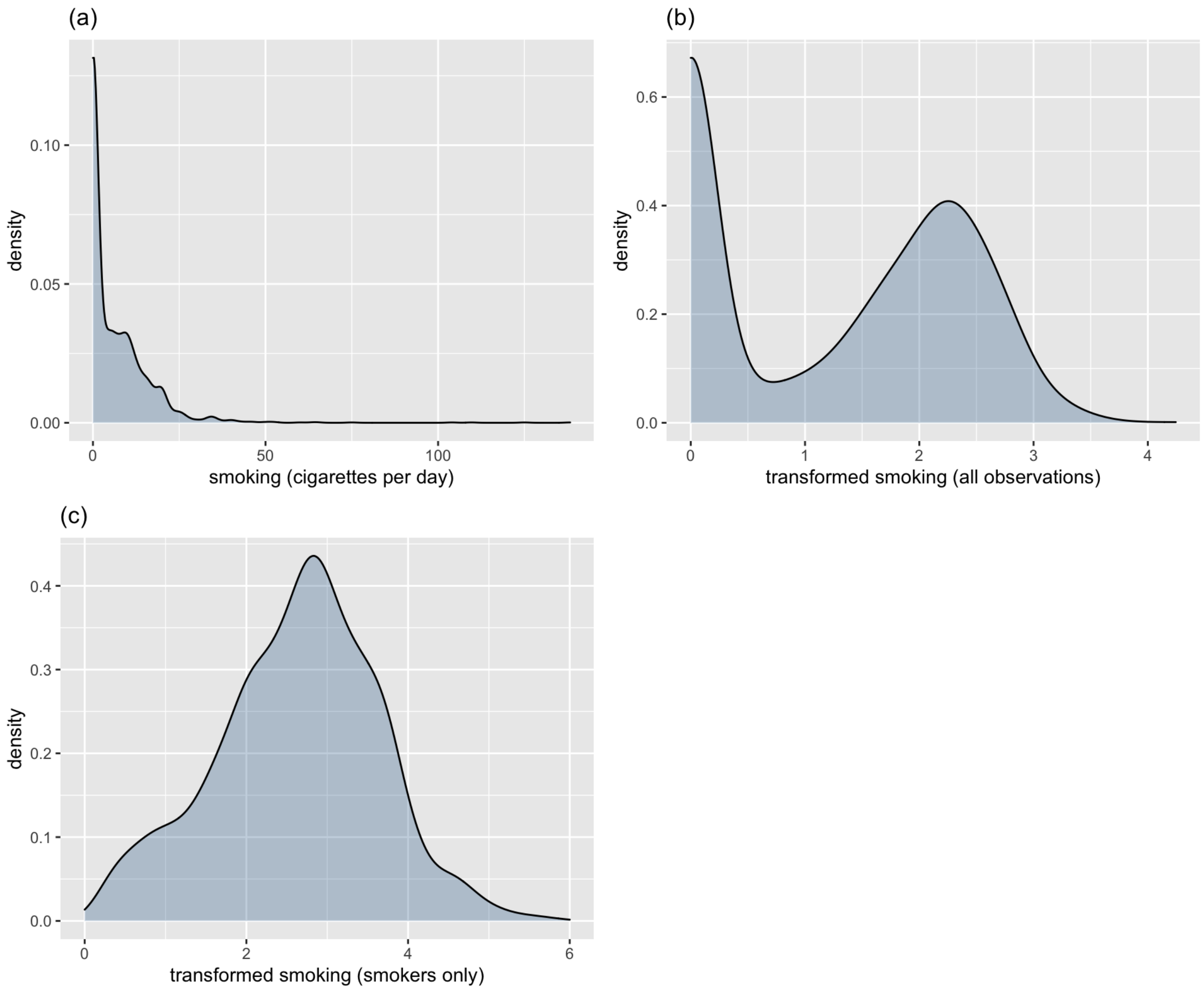

Figure S2: Density plots for the original and transformed smoking values. In parts (b) and (c), we used a Yeo-Johnson transformation for the smoking values based on all the participants and the smokers, respectively. The transformed smoking values are given by $T(x) = \frac{(x+1)^{\gamma}-1}{\gamma}$, where x and $T(x)$ are the original and transformed smoking values. γ is a tuning parameter obtained by conducting a grid searching to maximize the log-likelihood for the model $T(X) \sim N(\mu_x, \sigma_x^2)$, which equals to -0.074 and 0.104 for parts (b) and (c), respectively.

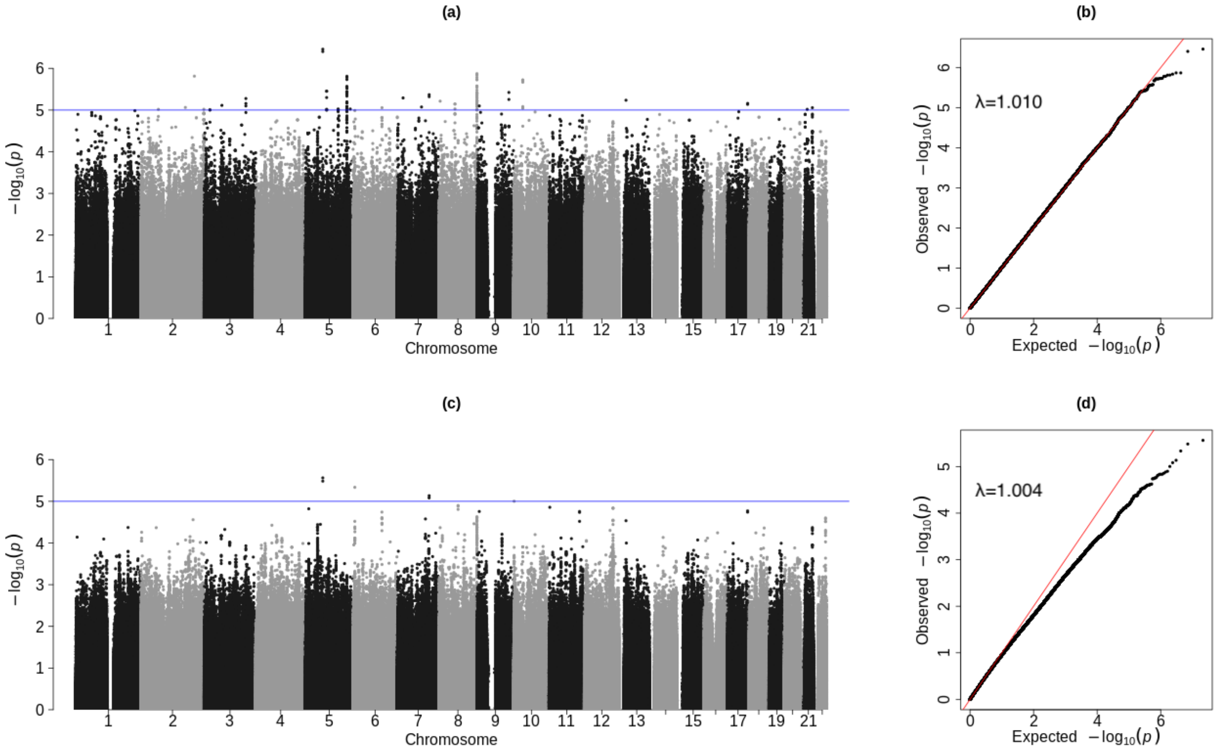

Figure S3: Manhattan plots and Quantile-Quantile plots for the test of gene-smoking interaction effect based on the smoker subgroup using the reverse test (upper panel) and standard logistic regression test (lower panel). In the Quantile-Quantile plots, λ denotes the genomic inflation factor. Blue line: suggestive level (P-value$= 1 \times 10^{-5}$).
